# Reducing Akt2 in retinal pigment epithelial cells causes a compensatory increase in Akt1 and attenuates diabetic retinopathy

**DOI:** 10.1038/s41467-022-33773-0

**Published:** 2022-10-13

**Authors:** Haitao Liu, Nadezda A. Stepicheva, Sayan Ghosh, Peng Shang, Olivia Chowdhury, Rachel A. Daley, Meysam Yazdankhah, Urvi Gupta, Stacey L. Hose, Mallika Valapala, Christopher Scott Fitting, Anastasia Strizhakova, Yang Shan, Derrick Feenstra, José-Alain Sahel, Ashwath Jayagopal, James T. Handa, J. Samuel Zigler, Patrice E. Fort, Akrit Sodhi, Debasish Sinha

**Affiliations:** 1 Department of Ophthalmology, University of Pittsburgh School of Medicine, Pittsburgh, PA USA; 2 School of Optometry, Indiana University, Bloomington, IN USA; 3 Kellogg Eye Center, University of Michigan School of Medicine, Ann Arbor, MI USA; 4 Pharma Research and Early Development, Roche Innovation Center Basel, F. Hoffmann-La Roche, Ltd., Basel, Switzerland; 5 Institut de la Vision, INSERM, CNRS, Sorbonne Université, Paris, France; 6 Opus Genetics, Durham, NC USA; 7 The Wilmer Eye Institute, The Johns Hopkins University School of Medicine, Baltimore, MD USA; 8 Present Address: Doheny Eye Institute, Pasadena, CA USA; 9 Present Address: Neural Stem Cell Institute, Rensselaer, NY USA

**Keywords:** Diabetes, Retinal diseases

## Abstract

The retinal pigment epithelium (RPE) plays an important role in the development of diabetic retinopathy (DR), a leading cause of blindness worldwide. Here we set out to explore the role of Akt2 signaling—integral to both RPE homeostasis and glucose metabolism—to DR. Using human tissue and genetically manipulated mice (including RPE-specific conditional knockout (cKO) and knock-in (KI) mice), we investigate whether Akts in the RPE influences DR in models of diabetic eye disease. We found that Akt1 and Akt2 activities were reciprocally regulated in the RPE of DR donor tissue and diabetic mice. *Akt2* cKO attenuated diabetes-induced retinal abnormalities through a compensatory upregulation of phospho-Akt1 leading to an inhibition of vascular injury, inflammatory cytokine release, and infiltration of immune cells mediated by the GSK3β/NF-κB signaling pathway; overexpression of Akt2 has no effect. We propose that targeting Akt1 activity in the RPE may be a novel therapy for treating DR.

## Abstract (web summary)

Retinal pigment epithelium (RPE) plays an important role in diabetic retinopathy (DR) progression. Here, the authors show AKT1 and AKT2 are reciprocally regulated in the RPE of DR donor tissue and diabetic mice, reducing Akt2 in RPE causing a compensatory increase in Akt1 and attenuating DR.

## Introduction

Diabetic retinopathy (DR) is the most common microvascular complication of diabetes, and is the leading cause of blindness among working-age adults in the developed world^1–4^. DR can be clinically classified into two stages: non-proliferative and proliferative^5^. The prevailing hypothesis is that the proliferative phase is secondary to the capillary degeneration that occurs during the progression of the non-proliferative stage^6,7^. Therapy that halts capillary degeneration in the early stages of the disease may be beneficial in delaying the progression to proliferative DR and severe irreversible vision loss^8^.

Many retinal cell types are affected in DR, such as intense loss of inner retinal neurons^9^, dysfunction of Müller cells^10^ and astrocytes^11,12^, and activation of microglia^13^. The interaction between inner retinal cells and retinal vascular lesions^14,15^, as well as the influence of photoreceptors (inflammatory signals)^16,17^, retinal ganglion cells (angiogenic factors)^18^, astrocytes (glucose metabolism and mitochondrial function)^12^, and microglia (immune response)^13^ on retinal vascular abnormalities in diabetes have been extensively studied. These findings have greatly improved our appreciation for the multiple signaling pathways and myriad of cell types in the retina that contribute to the pathogenesis of DR.

In contrast to the role of the neurosensory retinal cells, the contribution of the retinal pigment epithelium (RPE) in early DR remains less clear. The RPE is a monolayer of polarized multifunctional pigmented cells that form the outer blood-retina barrier (BRB) and is crucial for maintenance of retinal function^19–21^. Recent studies suggest that the RPE may also contribute to the development of DR^22,23^. Loss of insulin receptor-mediated signaling in the RPE reduced both the levels of reactive oxygen species and the expression of pro-inflammatory cytokines in the retinas of diabetic mice, providing evidence that the RPE could be involved in the development of DR^24^. Indeed, treatment with the visual cycle inhibitor retinylamine (Ret-NH2), which selectively inhibits RPE65 in the RPE, prevents diabetes-induced retinal capillary degeneration^23^. However, how the RPE affects retinal blood vessels in diabetes and how diabetes-induced changes in the RPE trigger infiltration and/or activation of immune cells in the retina remain unclear.

To narrow down the signaling pathways within the RPE that might contribute to the progression of DR, we focused on Akt signaling since it is integral to both RPE homeostasis and glucose metabolism^25–27^. Akt has three isoforms, Akt1, Akt2 and Akt3 that are encoded by three separate genes^28^. Akt1 and Akt3 are the main isoforms in the retina and their activity is affected by diabetes^29,30^, Akt2 is also reported to be expressed in the retina, which suggests functional redundancy^31^. However, it has been demonstrated that in photoreceptors, the neuroprotective role of Akt2 could not be compensated by either Akt1 or Akt3, which is in contrast to the functional overlap and the compensation observed in whole-body knockouts^32–34^. Akt1 is an important regulator of cell survival and protein synthesis, and homozygous disruption results in extensive growth retardation^35^. Because Akt3 is predominantly expressed in the brain, whole-body *Akt3* knockout mice have a small brain size^36^. Importantly, Akt2 is the isoform that has been most studied in diabetes because it acts downstream of insulin receptor (IR) signaling^32,37^. Notably, while Akt2 expression is generally lower than that of Akt1, it is expressed at high levels in insulin-responsive tissues, and plays an important role in maintaining glucose metabolism^38^. Indeed, a family with loss of Akt2 function due to a missense mutation displays severe insulin resistance and diabetes, just as *Akt2* global knockout mice display a severe type-II diabetes phenotype^38,39^. However, no retina phenotype was reported in this study. Furthermore, Akt2 is activated in the retinas of mice following sorbitol-induced hyperosmotic stress, which mimics aspects of diabetic hyperglycemia^40^. However, a contribution for Akt2 expression in the RPE to the development of DR has not been demonstrated.

Here, we show Akt2 and Akt1 activities are reciprocally regulated in the RPE of DR donor tissue and diabetic mice. RPE-specific knockout of *Akt2* attenuates diabetes-induced retinal molecular alterations and vascular lesions. Further, overexpressing Akt1 in RPE inhibits diabetes-induced retinal abnormalities and loss of Akt1 function in retina/RPE accelerates retinal vascular damage in diabetic mice. Thus, this work provides a foundation for targeting Akt1 as a promising therapeutic approach for the treatment of DR.

## Results

### The diabetes-induced impairment of retinal function is rescued in RPE-specific *Akt2* cKO mice

To address the role of Akt2 signaling in DR, RPE-specific *Akt2* cKO mice were generated (Supplementary Fig. 1a). The changes in the Akt2 level were validated by western blotting, confirming that both phospho- and total-Akt2 levels were decreased in the RPE from *Akt2* cKO mice compared to *Akt2*^fl/fl^ controls (Supplementary Fig. 1b). *Akt2* is not completely knocked out in *Akt2* cKO mice due to the mosaic expression of the Best1 gene (the Cre gene utilizes the Best1 promoter) in the RPE^41^, resulting in heterogenous loss of the floxed allele. Accordingly, immunofluorescence studies for Cre performed on *Akt2* cKO RPE flatmounts demonstrated the mosaic expression of Cre protein in RPE cells (Supplementary Fig. 1c). Diabetes was induced by intraperitoneal (IP) injection of streptozotocin (STZ); the successful induction of diabetes was confirmed by an increase in non-fasting blood glucose (>275 mg/dL) levels and the inability of the mice to gain body weight (Supplementary Table 1)^42^. In addition, hemoglobin A1c (HbA1c) was measured to assess the severity of hyperglycemia over time (HbA1c is typically 3.0% in nondiabetic mice and between 7.5 and 8.6% in diabetic mice; Supplementary Table 1)^42^. The severity of diabetes in the *Akt2* cKO mice was similar to *Akt2*^fl/fl^ controls. Blood glucose, HbA1c, and body weights were similar among the nondiabetic groups.

**Fig. 1:**
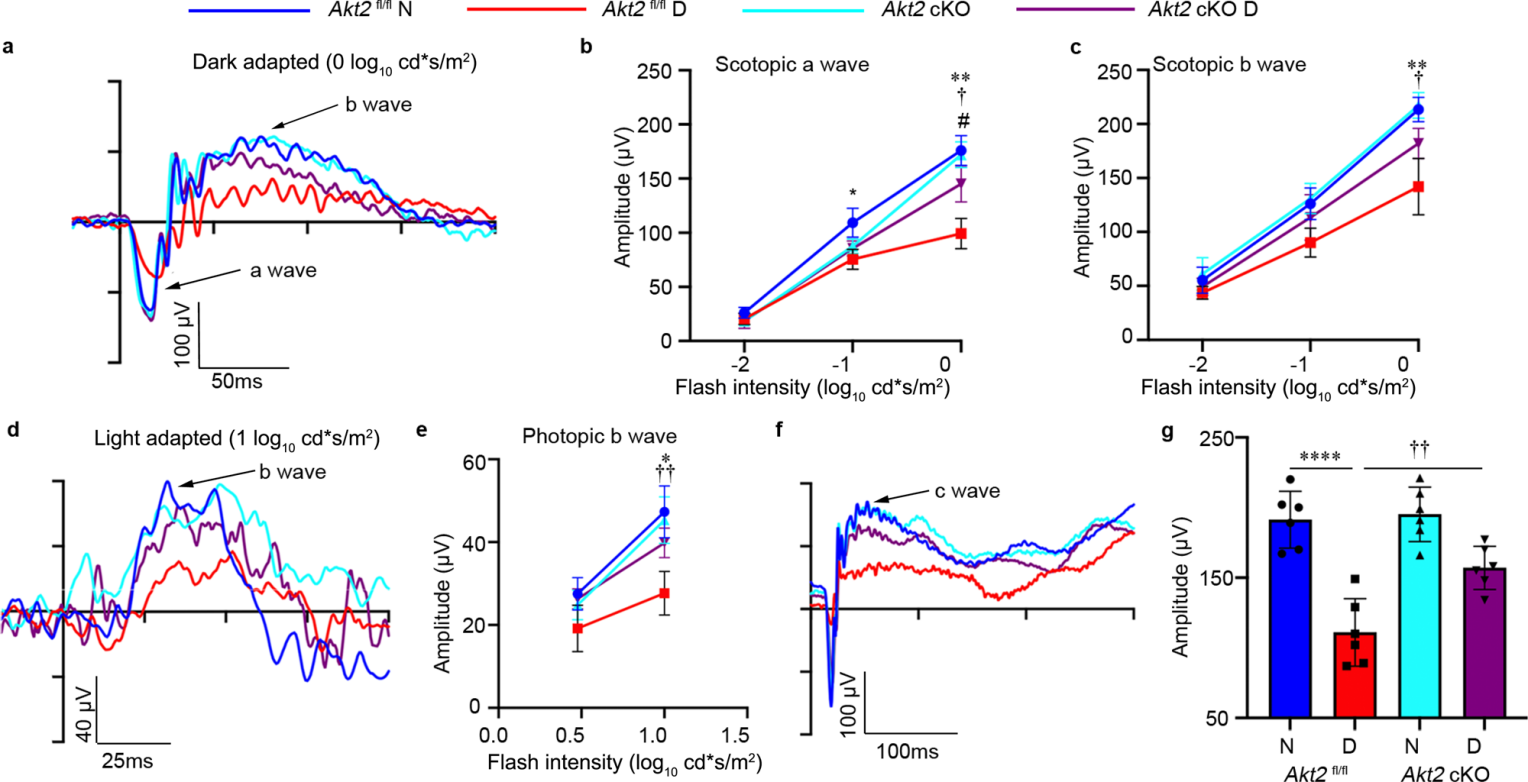
Electroretinography (ERG) suggests that RPE-specific *Akt2* cKO partially rescues diabetes-induced disruption of retinal function (4 month duration of diabetes). **a** Representative scotopic ERG a- and b-waveforms, showing response to a 0 log_10_ cd·s/m^2^ stimulus luminance after overnight dark adaptation. Scotopic (**b**) a-wave and (**c**) b-wave amplitudes were decreased in diabetic *Akt2*^fl/fl^ mice compared to nondiabetic controls. These changes were partially rescued in RPE-specific *Akt2* cKO diabetic mice. **d** Representative photopic ERG waveforms response to a 1 log_10_ cd·s/m^2^ stimulus luminance after light adaptation. **e** Induction of diabetes decreased the photopic b-wave amplitude in diabetic *Akt2*^fl/fl^ mice, which was rescued in RPE-specific *Akt2* cKO diabetic mice. **f** Representative ERG c-waveforms. (g) Diabetes decreased the c-wave amplitude in *Akt2*^fl/fl^ mice compared to nondiabetic animals, which was partially mitigated in *Akt2* cKO diabetic mice. In (**b**, **c**, **e**, **g**), *n* = 6 mice for each group, the data are expressed as mean ± SD. *p < 0.05; **p < 0.01; ****p < 0.0001 shows changes between diabetic *Akt2*^fl/fl^ and *Akt2*^fl/fl^ nondiabetic control. ^†^*p* < 0.05, ^††^*p* < 0.01 represents changes between diabetic *Akt2* cKO and diabetic *Akt2*^fl/fl^ mice. ^#^*p* < 0.05 represents changes of diabetic *Akt2* cKO versus nondiabetic *Akt2* cKO mice. Statistical test used in (**b**, **c**, **e**) is Two-way ANOVA followed by Tukey’s multiple comparisons test, and in (**g**) is One-way ANOVA followed by a Tukey’s post hoc test. Exact *p* values are: **b** At −1 log_10_ cd*s/m^2^, *p* = 0.0116 (*Akt2* ^fl/fl^ D vs. *Akt2* ^fl/fl^ N); At 0 log_10_ cd*s/m^2^, *p* = 0.0017 (*Akt2* ^fl/fl^ D vs. *Akt2* ^fl/fl^ N), *p* = 0.0357 (*Akt2* cKO D vs. *Akt2* ^fl/fl^ D), *p* = 0.0244 (*Akt2* cKO D vs. *Akt2* cKO N). **c** *p* = 0.0065 (*Akt2* ^fl/fl^ D vs. *Akt2* ^fl/fl^ N), *p* = 0.037 (*Akt2* cKO D vs. *Akt2* ^fl/fl^ D). **e** *p* = 0.0247 (*Akt2* ^fl/fl^ D vs. *Akt2* ^fl/fl^ N), *p* = 0.0046 (*Akt2* cKO D vs. *Akt2* ^fl/fl^ D). **g** *p* < 0.0001 (*Akt2* ^fl/fl^ D vs. *Akt2* ^fl/fl^ N), *p* = 0.0039 (*Akt2* cKO D vs. *Akt2* ^fl/fl^ D). N nondiabetic, D diabetic, cKO conditional knockout.

It has previously been reported that degenerative changes in the retina are early events in the development of DR^43^. Therefore, we tested retinal function by electroretinography (ERG), which allows for effective assessment of photoreceptor activity (a-wave), Müller and bipolar cell activity (b-wave), and RPE function (c-wave)^44^. With prolonged diabetes (4 months after induction) the amplitudes of dark-adapted scotopic a- and b-waves (Fig. 1a–c), as well as the light-adapted photopic b-wave were reduced in *Akt2*^fl/fl^ mice compared to nondiabetic controls at high flash levels (Fig. 1d, e). Importantly, the c-wave amplitude in diabetic *Akt2*^fl/fl^ mice compared to nondiabetic animals was also decreased (Fig. 1f, g), indicating that the retinal and RPE cells are severely affected. Notably, RPE-specific *Akt2* cKO reversed the diabetes-induced impairment of the retina and RPE function. We also found that scotopic and photopic ERGs are similar in *Akt2* cKO and *Akt2*^fl/fl^ diabetic mice (Fig. 1a–c) at low flash levels, suggesting that cones may be primarily affected. We next investigated cone and rod morphology under normal and diabetic conditions (Supplementary Fig. 2a) and observed that cones (stained with opsin antibody; red) are disorganized in *Akt2*^fl/fl^ diabetic mice. However, *Akt2* cKO normalized cone morphology in diabetic animals, as evident from the parallel orientation and highly organized structure of the cone photoreceptor outer and inner segments, consistent with a previous publication^45^. Interestingly, rod cell (stained with rhodopsin; green) morphology was similar between *Akt2*^fl/fl^ and *Akt2* cKO nondiabetic and diabetic mouse retinas (at a 2 month duration of diabetes). Since cones constitute ~3% of the photoreceptor population in mice^46^, gradual loss of both rods and cones would result in a greater percentage loss of cones than rods at early time points and hence loss of cone function would be identifiable prior to rod dysfunction, as reported here. Collectively, these results suggest that the RPE is not just passively affected by diabetes but also directly contributes to changes in the neural retina, and that Akt2 signaling in the RPE might play a central role. This is consistent with prior reports implicating the RPE in the development of diabetic retinopathy^23,24^. We therefore set out to further delineate the molecular mechanism whereby Ak2 signaling in the RPE helps maintain retinal vascular homeostasis in diabetes.

### Loss of Akt2 function in the RPE prevents increased levels of inflammatory proteins, leukostasis, and production of ROS in the diabetic retina

**Fig. 2:**
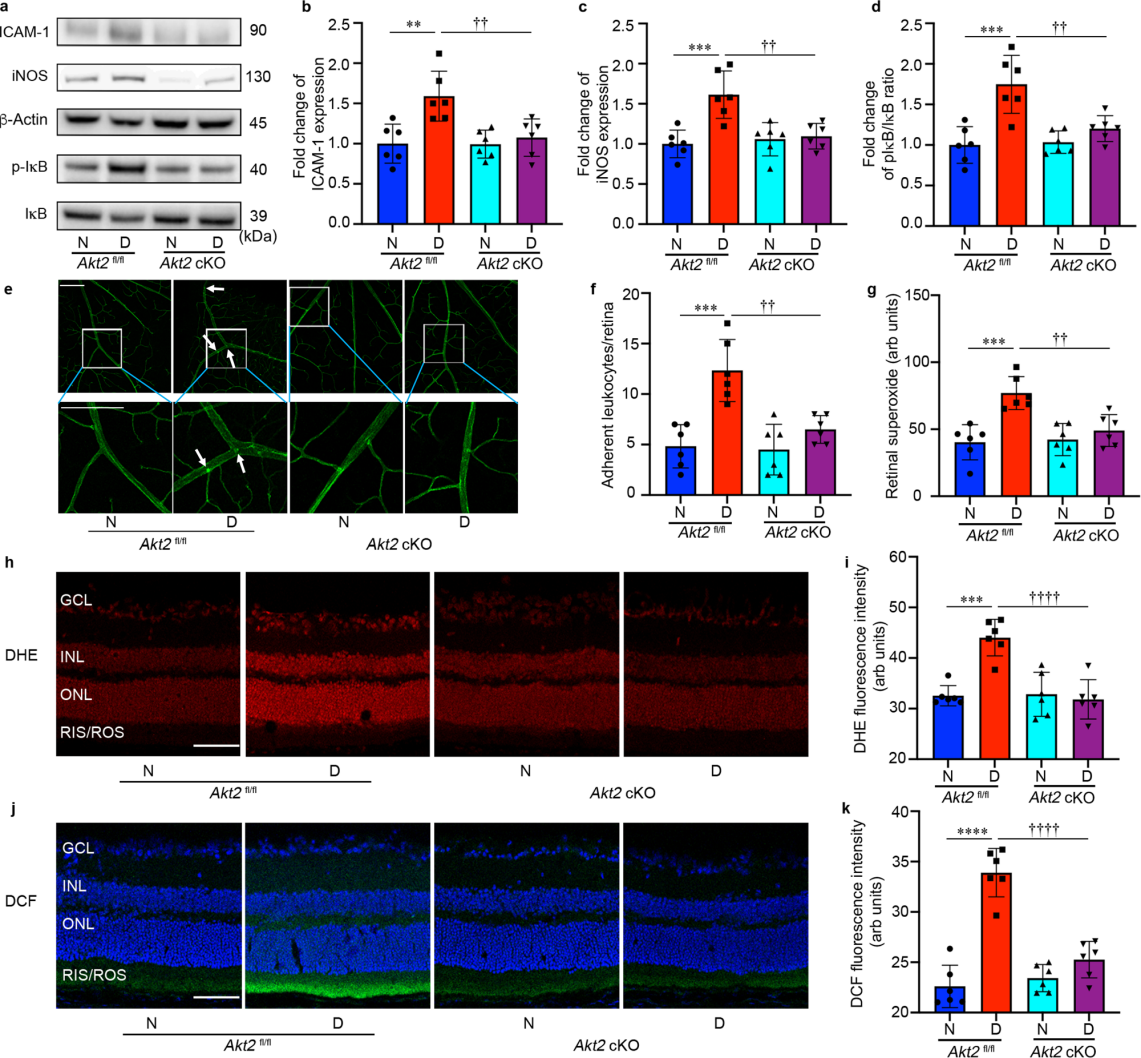
RPE-specific *Akt2* cKO inhibits the diabetes-induced increases in inflammatory proteins, leukostasis, and generation of reactive oxygen species in the mouse retina after 2 months of diabetes. **a** Representative immunoblots and densitometry graphs demonstrating the diabetes-induced increases of (**b**) ICAM-1, (**c**) iNOS, and (**d**) the ratio of pIκB/IκB were inhibited in the retina of RPE-specific *Akt2* cKO diabetic mice. **e** Representative images and (**f**) quantification of attached leukocytes in the retina. Arrows indicate leukocytes adherent to the retinal blood vessels. Diabetes increased the number of adherent leukocytes in the *Akt2*^fl/fl^ diabetic retina compared to nondiabetic *Akt2*^fl/fl^ mice; this number was markedly reduced in *Akt2* cKO diabetic mice, Scale bar: 100 µm. **g** Retinal superoxide was measured using lucigenin; diabetes increased retinal production of superoxide in *Akt2*^fl/fl^ mice. These levels were attenuated in *Akt2* cKO diabetic mice. **h** Representative images and (**i**) quantification of DHE-stained (red) retinal sections showing the levels of ROS in each group. The DHE stain was primarily localized in the nuclear layers, Scale bar: 100 µm. The intensity of red fluorescence was quantified at the INL and ONL as they represent the majority of red staining in the retina. The diabetes-induced increase of ROS in *Akt2*^fl/fl^ retina was not observed in diabetic *Akt2* cKO mice. **j** Representative images and (**k**) quantification of DCF-stained retinal sections showing ROS accumulated in the inner and outer segments of photoreceptors. The blue stain is DAPI. The diabetic *Akt2* cKO mice displayed significantly low retinal ROS levels compared to the diabetic *Akt2*^fl/fl^ mice. Scale bar: 100 µm. Data are expressed as mean ± SD. **p < 0.01; ***p < 0.001; ****p < 0.0001 show changes versus *Akt2*^fl/fl^ nondiabetic (N) controls. ^††^*p* < 0.01, and ^††††^*p* < 0.0001 show changes versus *Akt2*^fl/fl^ diabetic (D) mice. Statistical test used in (**b**, **c**, **d**, **f**, **g**, **i**, **k**) is One-way ANOVA followed by a Tukey’s post hoc test. *n* = 6 mice for each group. Exact *p* values are: **b** *p* = 0.0024 (*Akt2* ^fl/fl^ D vs. *Akt2* ^fl/fl^ N), *p* = 0.0081 (*Akt2* cKO D vs. *Akt2* ^fl/fl^ D). **c** *p* = 0.0004 (*Akt2* ^fl/fl^ D vs. *Akt2* ^fl/fl^ N), *p* = 0.0024 (*Akt2* cKO D vs. *Akt2* ^fl/fl^ D). **d** *p* = 0.0001 (*Akt2* ^fl/fl^ D vs. *Akt2* ^fl/fl^ N), *p* = 0.0036 (*Akt2* cKO D vs. *Akt2* ^fl/fl^ D). **f** *p* = 0.0001 (*Akt2* ^fl/fl^ D vs. *Akt2* ^fl/fl^ N), *p* = 0.0019 (*Akt2* cKO D vs. *Akt2* ^fl/fl^ D). **g** *p* = 0.0003 (*Akt2* ^fl/fl^ D vs. *Akt2* ^fl/fl^ N), *p* = 0.0043 (*Akt2* cKO D vs. *Akt2* ^fl/fl^ D). **i** *p* = 0.0001 (*Akt2* ^fl/fl^ D vs. *Akt2* ^fl/fl^ N), *p* < 0.0001 (*Akt2* cKO D vs. *Akt2* ^fl/fl^ D). **k** *p* < 0.0001 (*Akt2* ^fl/fl^ D vs. *Akt2* ^fl/fl^ N, *Akt2* cKO D vs. *Akt2* ^fl/fl^ D). cKO conditional knockout, GCL ganglion cell layer, INL inner nuclear layer, ONL outer nuclear layer, RIS/ROS rod inner/outer segment, DHE dihydroethidium, DCF dichlorofluorescein. Source Data is provided in the Source Data file.

Retinal inflammation, leukostasis and oxidative stress have all been implicated in early DR pathogenesis^47–49^. We previously reported that Akt2 regulates inflammation in the RPE cells^50^. To determine if Akt2 in RPE cells influences inflammatory proteins, leukostasis and the production of ROS in the diabetic retina, RPE-specific *Akt2* cKO mice, along with *Akt2*^fl/fl^ controls, were analyzed after 2 months of diabetes. The diabetes-induced increases in the level of inflammatory proteins iNOS and ICAM-1, and in the ratio of pIκB/IκB in the neural retina were inhibited in *Akt2* cKO diabetic mice compared to *Akt2*^fl/fl^ diabetic controls (Fig. 2a–d). This inhibition suggests that decreasing Akt2 specifically in the RPE protects against diabetes-induced inflammatory changes in the neural retina. ICAM-1 is known to mediate the attachment of leukocytes to retinal endothelial cells, thus promoting leukostasis, an important pathogenic event in the development of DR^51^. Interestingly, we observed fewer leukocytes attached to the retinal vasculature of RPE-specific *Akt2* cKO diabetic mice compared to *Akt2*^fl/fl^ diabetic mice (Fig. 2e, f). Taken together, these data suggest that Akt2 in the RPE promotes the diabetes-induced increase of neural retinal inflammation that mediates leukostasis within the retinal vasculature through ICAM-1.

Diabetes-induced increase of retinal superoxide and leukostasis were reported to be inhibited in iNOS-deficient mice, suggesting that ROS production is linked to inflammation^52^. Moreover, antioxidant therapies such as vitamin C and E, or overexpression of superoxide dismutase 1 (SOD1) in diabetic mice inhibit capillary degeneration, indicating that oxidative stress is a critical pathogenic factor in microvascular injury in the diabetic retina^53,54^. Thus, we examined retinal ROS production in these mouse models. ROS generated in the retina were assessed using freshly isolated retinas incubated with lucigenin (which reacts with superoxide), and by staining cryosections of the retinas with dihydroethidium (DHE, which reacts with superoxide) and 2′,7′ dichlorodihydrofluorescein diacetate (DCF, which reacts with ROS). Superoxide production was markedly attenuated in RPE-specific *Akt2* cKO diabetic mice (Fig. 2g). DHE and DCF staining enabled us to visualize the localization of ROS within the retina. DHE turns red in the presence of superoxide and intercalates within DNA thereby staining nuclei. We found that diabetes-induced superoxide is mainly generated in the inner and outer nuclear layers (INL/ONL) of the retina (Fig. 2h). The red fluorescence intensity was lower in the RPE-specific *Akt2* cKO diabetic mice compared to *Akt2*^fl/fl^ diabetic controls (Fig. 2i). DCF turns green when reacting with ROS (Fig. 2); this staining revealed an increase in ROS levels in the photoreceptor inner/outer segments of diabetic *Akt2*^fl/fl^ controls, but not in diabetic *Akt2* cKO mice (Fig. 2k). Collectively, these data suggest that retinal production of ROS, one of the important contributors to DR, may be caused by activation of iNOS by Akt2 in RPE in the diabetic retina.

### Deleting Akt2 in the RPE inhibits diabetes-induced infiltration of leukocytes in the retina/RPE

**Fig. 3:**
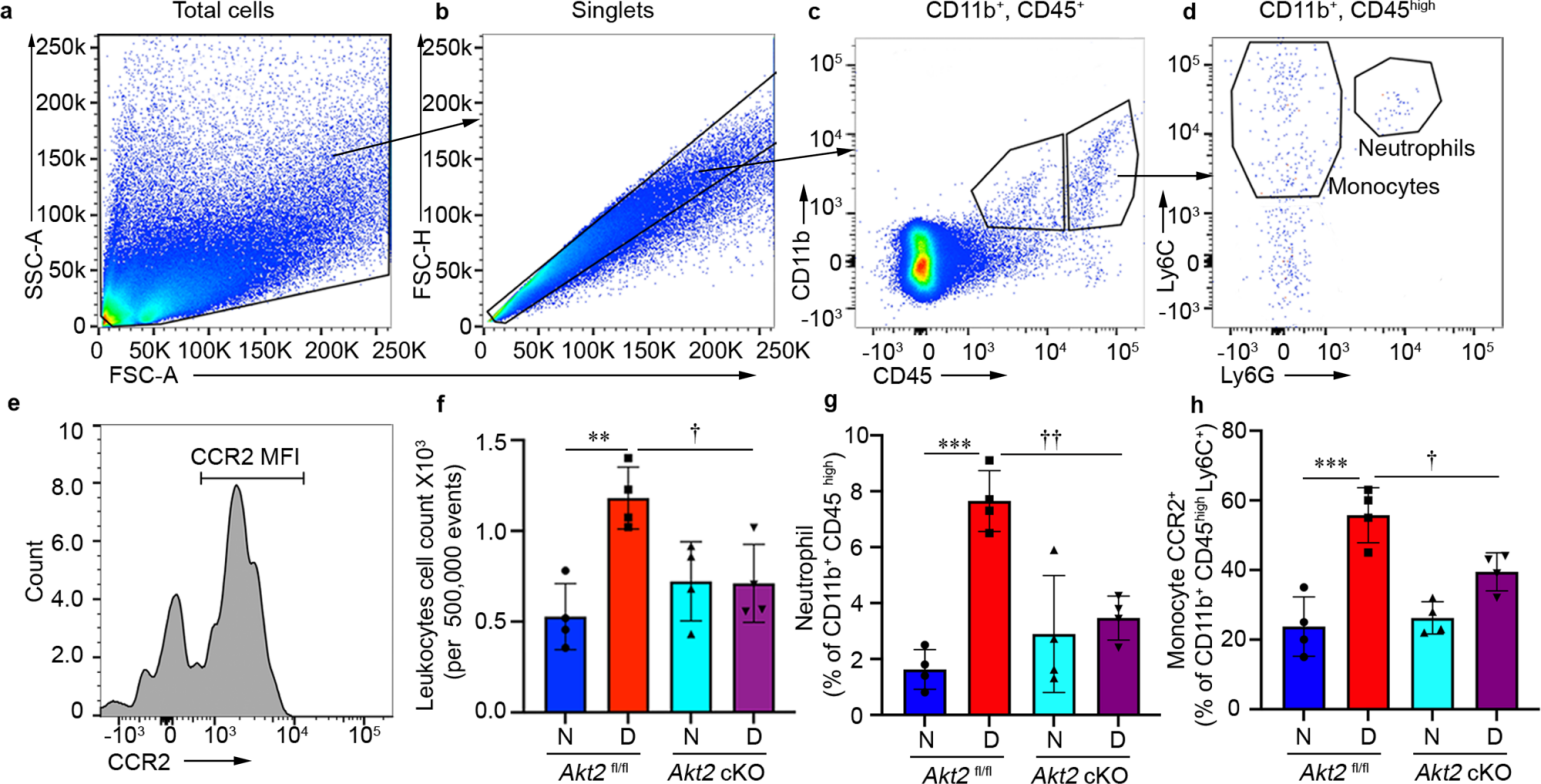
Flow cytometry to identify the population of leukocytes infiltrating the RPE and retina isolated from diabetic (2 months of diabetes) and age-matched nondiabetic *Akt2*^fl/fl^ and *Akt2* cKO mice. **a** FSC-A versus SSC-A dot plots were gated to eliminate debris. **b** Single cells were selected on FSC-A versus FSC-H. **c** Representative dot plots were gated on CD11b^+^ and CD45^+^ cells. **d** Ly6C, Ly6G and (**e**) CCR2 antibodies were used to define the neutrophils and monocytes gated on CD11b^+^CD45^high^ cells. **f** The absolute number of infiltrating leukocytes (CD11b^+^CD45^high^) was increased in *Akt2*^fl/fl^ diabetic mice. However, this was inhibited in RPE-specific *Akt2* cKO diabetic mice. **g** The neutrophil and (**h**) monocyte populations were increased in the retinas of *Akt2*^fl/fl^ diabetic mice compared to nondiabetic controls. Interestingly, *Akt2* cKO mice demonstrated noticeable decline in the diabetes-induced increase in retinal neutrophil and monocyte infiltration. In (**f**–**h**), data are shown as Mean ± SD. N = 4 animals per group. **p < 0.01 and ***p < 0.001 denotes changes versus *Akt2*^fl/fl^ nondiabetic group. ^†^*p* < 0.05, and ^††^*p* < 0.01, shows changes versus *Akt2*^fl/fl^ diabetic mice. Statistical test used in (**f**–**h**) is One-way ANOVA followed by a Tukey’s post hoc test. Exact p values are: **f** *p* = 0.0026 (*Akt2* ^fl/fl^ D vs. *Akt2* ^fl/fl^ N), *p* = 0.025 (*Akt2* cKO D vs. *Akt2* ^fl/fl^ D) **g** *p* = 0.0001 (*Akt2* ^fl/fl^ D vs. *Akt2* ^fl/fl^ N), *p* = 0.0031 (*Akt2* cKO D vs. *Akt2* ^fl/fl^ D). **h** *p* = 0.0001 (*Akt2* ^fl/fl^ D vs. *Akt2* ^fl/fl^ N), *p* = 0.025 (*Akt2* cKO D vs. *Akt2* ^fl/fl^ D).

Previous studies showed that recruitment of inflammatory cells plays an important role in diabetic eye disease^49,55^. Indeed, leukocytes isolated from diabetic patients and mice have been shown to induce the death of retinal endothelial cells^56,57^. Moreover, retinal capillary degeneration in diabetic animals is inhibited if ICAM-1 is absent or Neutrophil Inhibitory Factor (NIF) is overexpressed^58,59^. Further studies also showed that neutrophil elastase secreted by neutrophils contribute to retinal vascular leakage in DR^60^. To determine if the protective effects observed in *Akt2* cKO mice could be due to reduced infiltration of leukocytes into the retina, we performed a quantitative analysis of the leukocytes in retinas from these mice. To this end, we isolated the retina and RPE from diabetic (2 months of diabetes) and age-matched nondiabetic mice. Each retina/RPE preparation was digested to make a cell suspension, followed by immunostaining for flow cytometry to determine the extent of infiltration of various immune cells. The gating strategy is shown in Fig. 3a–e. As expected, diabetes increased the infiltration of leukocytes (CD11b^+^CD45^high^) in the retina of *Akt2*^fl/fl^ mice compared to nondiabetic controls. However, *Akt2* cKO diabetic mice had noticeably fewer infiltrating leukocytes in the retina/RPE compared to *Akt2*^fl/fl^ diabetic mice (Fig. 3f). We also observed that subsets within the infiltrating leukocyte population, specifically neutrophils (CD11b^+^CD45^high^Ly6C^high^Ly6G^+^) and monocytes (CD11b^+^CD45^high^Ly6C^High^CCR2^+^), were increased in the *Akt2*^fl/fl^ diabetic mice. These diabetes-induced increases were not observed in the *Akt2* cKO diabetic mice (Fig. 3g, h).

### Knockout of *Akt2* in the RPE prevents diabetes-induced retinal capillary degeneration and vascular leakage

**Fig. 4:**
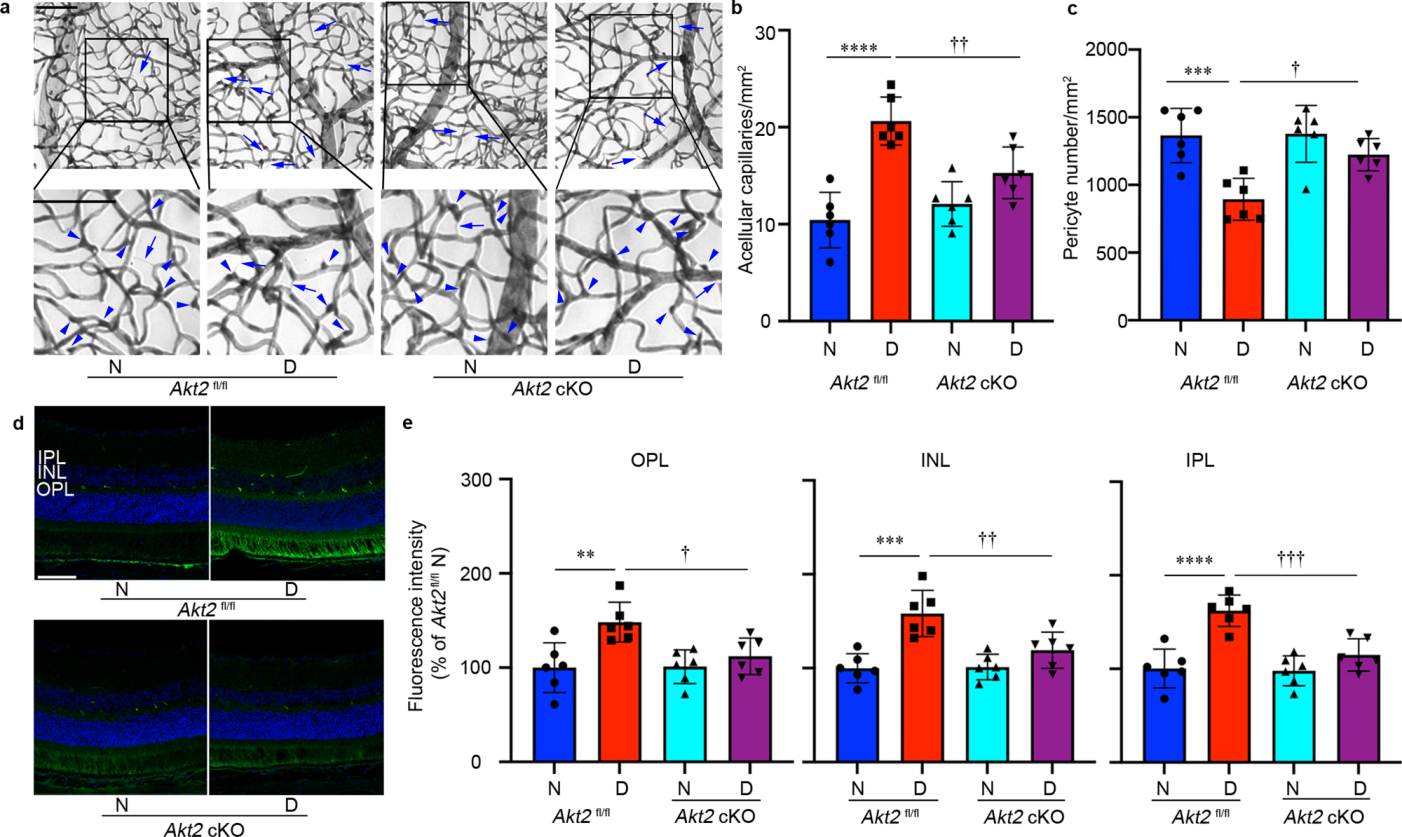
RPE-specific *Akt2* cKO inhibits the development of diabetes-induced retinal vascular lesions. **a** Representative micrographs of retinal vessels from diabetic mice (8 months of diabetes) and age-matched nondiabetic mice. Arrows indicate degenerated capillaries and arrowheads indicate capillary pericytes. Scale bar: 100 µm. **b** Diabetes increased the number of degenerated capillaries and (**c**) decreased the number of retinal capillary pericytes in diabetic *Akt2*^fl/fl^ mice compared to nondiabetic animals. RPE-specific *Akt2* cKO partially rescued this degeneration. **d** Representative micrographs of retinal sections after mice were intravenously injected with FITC-albumin. Scale bar: 100 µm. **e** Average fluorescence intensity was quantified from a large area of INL, IPL, and OPL, excluding obvious microvessels. RPE-specific *Akt2* cKO reduced the diabetes-induced accumulation of FITC-BSA in the OPL, INL, and IPL of mouse retina compared to diabetic *Akt2*^fl/fl^ mice. In (**b**, **c**, **e**), *n* = 6 mice for each group, the data are expressed as mean ± SD. **p < 0.01; ***p < 0.001; ****p < 0.0001 versus *Akt2*^fl/fl^ nondiabetic control (N). ^†^*p* < 0.05; ^††^*p* < 0.01; ^†††^*p* < 0.0001 versus *Akt2*^fl/fl^ diabetic mice (D). Statistical test used in (**b**, **c**, **e**) is One-way ANOVA followed by a Tukey’s post hoc test. Exact *p* values are: **b** *p* < 0.0001 (*Akt2* ^fl/fl^ D vs. *Akt2* ^fl/fl^ N), *p* = 0.0096 (*Akt2* cKO D vs. *Akt2* ^fl/fl^ D). **c** *p* = 0.0008 (*Akt2* ^fl/fl^ D vs. *Akt2* ^fl/fl^ N), *p* = 0.0187 (*Akt2* cKO D vs. *Akt2* ^fl/fl^ D). **e** OPL, *p* = 0.0046 (*Akt2* ^fl/fl^ D vs. *Akt2* ^fl/fl^ N), *p* = 0.0388 (*Akt2* cKO D vs. *Akt2* ^fl/fl^ D); INL, *p* = 0.0002 (*Akt2* ^fl/fl^ D vs. *Akt2* ^fl/fl^ N), *p* = 0.0087 (*Akt2* cKO D vs. *Akt2* ^fl/fl^ D). IPL, *p* < 0.0001 (*Akt2* ^fl/fl^ D vs. *Akt2* ^fl/fl^ N), *p* = 0.0009 (*Akt2* cKO D vs. *Akt2* ^fl/fl^ D). cKO conditional knockout, IPL inner plexiform layer, INL inner nuclear layer, OPL outer plexiform layer.

Collectively, these data demonstrate that knockout of *Akt2* in the RPE reduces inflammatory cell infiltration, leukostasis, and degeneration of the retina. To determine if these changes were sufficient to protect against retinal vascular lesions in DR, we examined the retinas of *Akt2*^fl/fl^ and *Akt2* cKO mice that had been diabetic for several months and manifested retinal vascular changes. The duration of diabetes was prolonged to 8 months because retinal capillary degeneration and vascular leakage becomes prominent by this stage. We observed an increase in acellular capillaries (Fig. 4a, arrows) and a decrease in capillary pericytes (Fig. 4a, arrowheads), both hallmarks of early-stage DR, in the retinas of *Akt2*^fl/fl^ diabetic mice compared to nondiabetic controls. However, these diabetes-induced defects were strikingly reduced in the retinas of *Akt2* cKO diabetic mice (Fig. 4b, c).

To evaluate retinal vascular leakage, mice were injected with fluorescein isothiocyanate-labeled bovine serum albumin (FITC-BSA) through the tail vein, and eyes were then fixed and sectioned. The amount of FITC-BSA that leaked from the circulating blood into the neural retina was measured on retinal cryosections in the IPL, INL, and OPL, excluding obvious microvessels (Fig. 4d). These three retinal layers were selected for measurement because their capillary plexuses are interconnected in the retina^23^. In 10 month-old mice, the amount of albumin that leaked in the neural retina was higher in *Akt2*^fl/fl^ diabetic mice compared to nondiabetic controls, while *Akt2* cKO lowered such diabetes-induced leakage (Fig. 4d, e). Of note, although clinical evidence in diabetic patients indicates vascular leakage can contribute to retinal thickening and edema, the thickness of the retina and of the ONL was not different in diabetic and age-matched nondiabetic mice used in this study (Supplementary Fig. 2). These data suggest that modulating Akt2 signaling within the RPE may play a pivotal role in both retinal capillary degeneration and vascular leakage in diabetes.

### Generation and characterization of a DR phenotype in *Akt2* KI (knockin) mice with RPE-specific overexpression *of* Akt2

Since loss of Akt2 function in the RPE protected against diabetes-induced retinopathy, we examined whether increased Akt2 would have the reverse effect by generating RPE-specific *Akt2* KI mice and inducing diabetes with STZ (Supplementary Fig. 3a). Diabetes was confirmed by a significant increase in the levels of non-fasting blood glucose (>275 mg/dL)^42^, and reduced increase in body weight (Supplementary Table 1)^42^. The severity of diabetes in the KI group and WT controls was not different. In nondiabetic groups, the blood glucose, HbA1c, and body weights were similar (Supplementary Table 1). We confirmed by western blotting that levels of both phospho- and total-Akt2 were higher in the RPE from *Akt2* KI mice compared to WT control mice (Supplementary Fig. 3b). Only a subset (about 60–70%) of RPE cells express the *Best1* gene in the wild type and *Akt2* KI mice (Supplementary Fig. 3c).

**Fig. 5:**
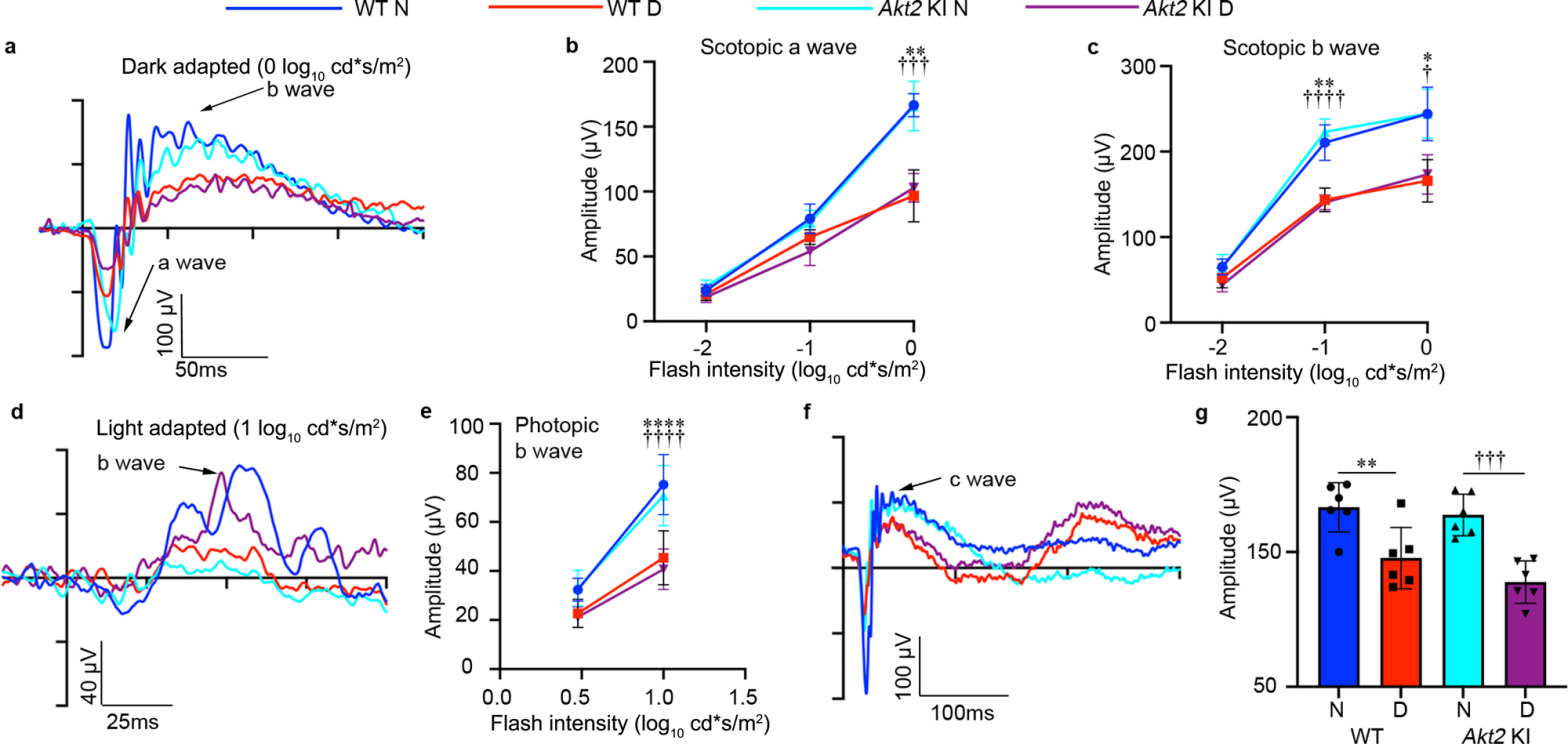
ERG suggests that RPE-specific *Akt2* KI does not impact the diabetes-induced disruption of retinal function (4 month duration of diabetes). **a** Representative scotopic ERG a- and b- waveforms, showing response to a 0 log_10_ cd·s/m^2^ stimulus luminance after overnight dark adaptation. Scotopic (**b**) a-wave and (**c**) b-wave amplitudes were decreased in both WT and *Akt2* KI diabetic mice compared to appropriate nondiabetic controls. *Akt2* overexpression in the RPE (*Akt2* KI) did not exhibit any protective effect on diabetic-induced ERG abnormalities. **d** Representative photopic ERG b-waveforms response to a 1 log_10_ cd·s/m^2^ stimulus luminance after light adaptation. **e** RPE-specific *Akt2* KI had no protective effect on the diabetes-induced detrimental changes in the photopic b-wave. **f** Representative ERG c-waveforms. **g** c-wave amplitude is decreased in WT and *Akt2* KI diabetic mice compared to appropriate nondiabetic controls. There was no significant change in the c-wave between diabetic WT and *Akt2* KI groups. In (**b**, **c**, **e**, **g**), *n* = 6 mice for each group, the data are expressed as mean ± SD. *p < 0.05; **p < 0.01; ***p < 0.001; ****p < 0.0001 denotes changes with respect to nondiabetic WT control. ^†^*p* < 0.05; ^†††^*p* < 0.001; ^††††^*p* < 0.0001 denotes changes versus nondiabetic *Akt2* KI mice. Statistical test used in (**b**, **c**, **e**) is Two-way ANOVA followed by Tukey’s multiple comparisons test. Test used in (**g**) is One-way ANOVA followed by a Tukey’s post hoc test. Exact *p* values are: **b** *p* = 0.0030 (WT D vs. WT N), *p* = 0.0003 (*Akt2* KI D vs. *Akt2* KI N). **c** At 0 log_10_ cd*s/m^2^ *p* = 0.0255 (WT D vs. WT N), *p* = 0.0147 (*Akt2* KI D vs. *Akt2* KI N); At −1 log_10_ cd*s/m^2^ *p* = 0.0020 (WT D vs. WT N), *p* < 0.0001 (*Akt2* KI D vs. *Akt2* KI N). **e** *p* < 0.0001 (WT D vs. WT N, *Akt2* KI D vs. *Akt2* KI N). **g** *p* = 0.0095 (WT D vs. WT N), *p* = 0.0006 (*Akt2* KI D vs. WT N), *p* = 0.0007 (*Akt2* KI D vs. *Akt2* KI N). N nondiabetic, D diabetic, WT wild type, KI knock-in.

**Fig. 6:**
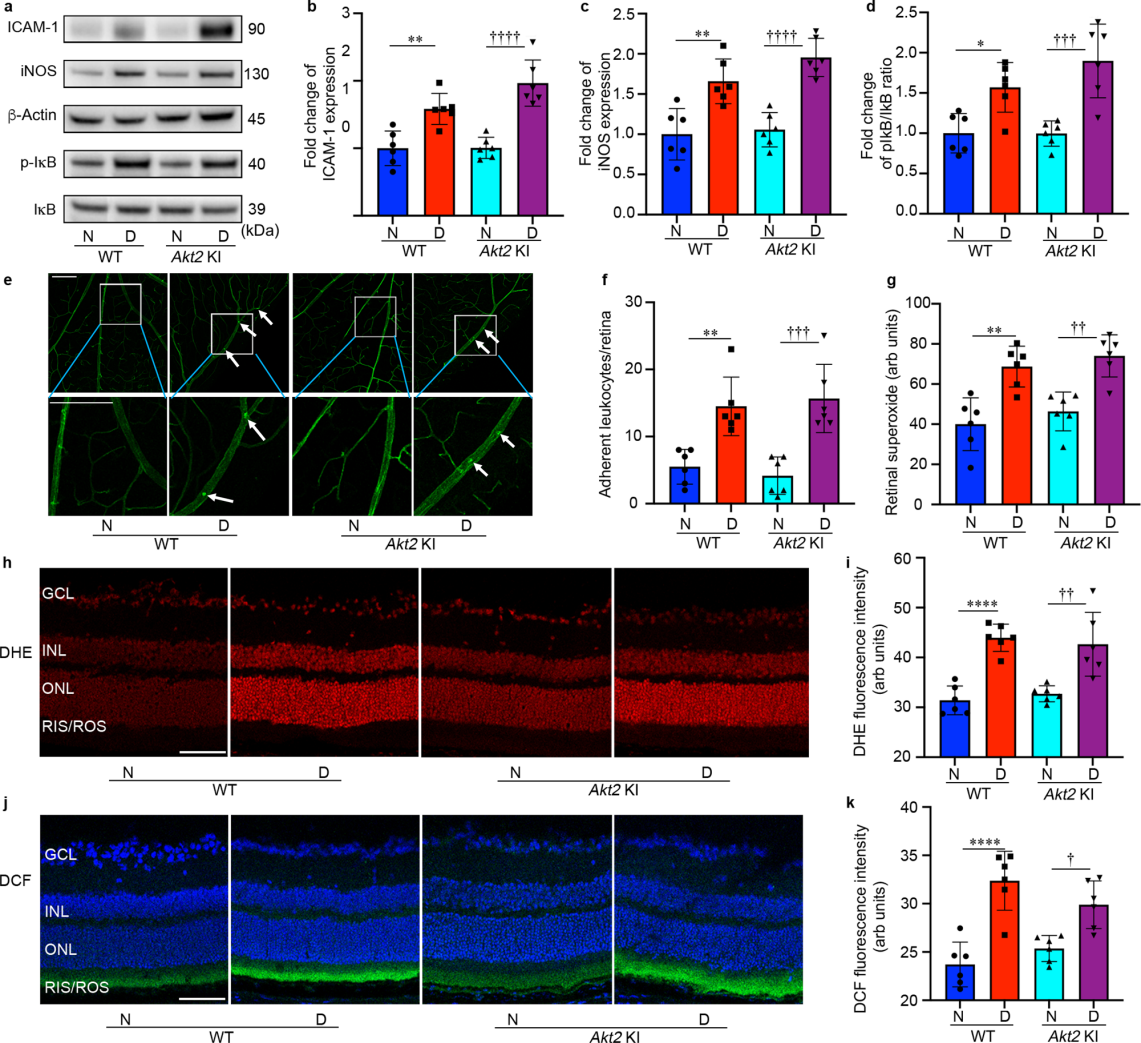
RPE-specific *Akt2* KI does not influence the diabetes-induced increase in pro-inflammatory protein level, leukostasis, or production of reactive oxygen species in mice after 2 months of diabetes. **a** Representative immunoblots and densitometry graphs showing that the diabetes-induced increase in the levels of pro-inflammatory markers (**b**) ICAM-1, (**c**) iNOS, and (**d**) the ratio of pIκB/ IκB were not significantly decreased in *Akt2* KI diabetic mice compared to WT diabetic mice. This result is in contrast to that found with *Akt2* cKO mice (**e**) Representative images and (**f**) quantification of retinal leukostasis from each group. Arrows indicate leukocytes adherent to the retinal blood vessels. Diabetes increased the number of adherent leukocytes in the retina of both WT and RPE-specific *Akt2* KI mice; Scale bar: 100 µm. **g** Retinal superoxide was measured using the lucigenin method. Diabetes increased retinal superoxide production in both diabetic WT and *Akt2* KI mice. **h** Representative images and (**i**) quantification of dihydroethidium (DHE)-stained (red) ROS. Scale bar: 100 µm. Red fluorescence intensity (DHE stain) was quantified at the INL and ONL together as they represent the majority of staining in the retina. Diabetes increased the production of retinal ROS in both WT and *Akt2* KI mice. **j** Representative images and (k) quantification of dichlorofluorescein (DCF) stained ROS. The DCF stain is localized primarily in the inner and outer segments of photoreceptors. The blue (nuclear) stain is DAPI. Diabetes induced an increase of ROS in the retina of both diabetic WT and *Akt2* KI mice compared to appropriate nondiabetic controls. Scale bars: 100 µm. In (**b**–**d**, **f**, **g**, **i**, **k**), *n* = 6 mice for each group, data are represented as mean ± SD. *p < 0.05, **p < 0.01, ***p < 0.001, and ****p < 0.0001 represent changes versus WT N. ^†^*p* < 0.05, ^††^*p* < 0.01, ^†††^*p* < 0.001, and ^††††^*p* < 0.0001 denotes changes versus *Akt2* KI N. Statistical test used in this study is One-way ANOVA followed by a Tukey’s post hoc test. Exact *p* values are: **b** *p* = 0.004 (WT D vs. WT N), *p* < 0.0001 (*Akt2* KI D vs. WT N, *Akt2* KI D vs. *Akt2* KI N). **c** *p* = 0.0019 (WT D vs. WT N), *p* < 0.0001 (*Akt2* KI D vs. WT N, *Akt2* KI D vs. *Akt2* KI N). **d** *p* = 0.0231 (WT D vs. WT N), *p* = 0.0004 (*Akt2* KI D vs. WT N), *p* = 0.0004 (*Akt2* KI D vs. *Akt2* KI N). **f** *p* = 0.0033 (WT D vs. WT N), *p* = 0.001 (*Akt2* KI D vs. WT N), *p* = 0.0003 (*Akt2* KI D vs. *Akt2* KI N). **g** *p* = 0.001 (WT D vs. WT N), *p* = 0.0002 (*Akt2* KI D vs. WT N), *p* = 0.0015 (*Akt2* KI D vs. *Akt2* KI N). **i** *p* < 0.0001 (WT D vs. WT N), *p* = 0.0003 (*Akt2* KI D vs. WT N), *p* = 0.0012 (*Akt2* KI D vs. *Akt2* KI N). **k** *p* < 0.0001 (WT D vs. WT N), *p* = 0.0011 (*Akt2* KI D vs. WT N), *p* = 0.017 (*Akt2* KI D vs. *Akt2* KI N). WT wild type, N nondiabetic, D diabetic, KI knock-in, GCL ganglion cell layer, INL inner nuclear layer, ONL outer nuclear layer, RIS/ROS rod inner/outer segment. Source Data is provided in the Source Data file.

**Fig. 7:**
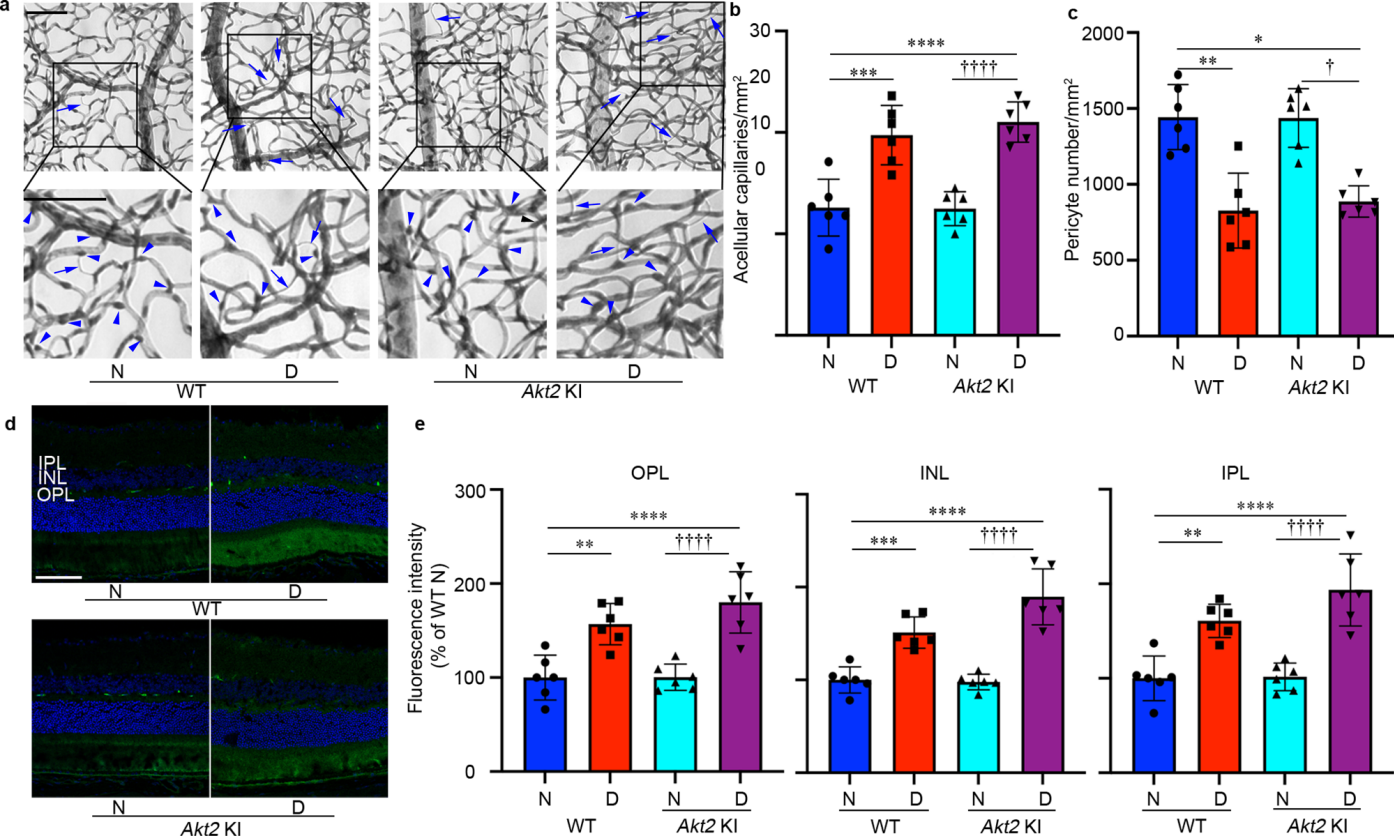
RPE-specific *Akt2* KI does not influence the development of diabetes-induced retinal vascular lesions. **a** Representative micrographs of retinal vessels from diabetic mice (8 months of diabetes) and age-matched nondiabetic mice. Scale bar: 100 µm. Arrows indicate degenerated capillaries and arrowheads indicate capillary pericytes. **b** Diabetes increased the number of degenerated capillaries and (**c**) decreased the number of retinal capillary pericytes in diabetic WT mice compared to nondiabetic animals. There was no significant difference in the numbers of acellular capillaries and pericytes in the retina between diabetic WT and diabetic *Akt2* KI mice. **d** Representative micrographs of retinal sections from each group after mice were intravenously injected with FITC-albumin. Scale bar: 100 µm. **e** Average fluorescence intensity was quantified from a large area of INL, IPL, and OPL, excluding obvious microvessels. Diabetes-induced accumulation of FITC-BSA in these retinal layers was higher in WT and *Akt2* KI diabetic mice (8 months of diabetes) compared to age-matched nondiabetic controls. There was no difference in diabetes-induced retinal vascular leakage between diabetic WT and diabetic *Akt2* KI mice. In (**b**, **c**, **e**), *n* = 6 mice for each group, the data are expressed as mean ± SD. *p < 0.05, **p < 0.01, ***p < 0.001, and ****p < 0.0001 shows changes versus WT nondiabetic control. ^†^*p* < 0.05 and ^††††^*p* < 0.0001 shows changes versus *Akt2* KI nondiabetic mice. Statistical test used in this study is One-way ANOVA followed by a Tukey’s post hoc test. Exact *p* values are: **b** *p* = 0.0003 (WT D vs. WT N), *p* < 0.0001 (*Akt2* KI D vs. WT N and *Akt2* KI D vs. *Akt2* KI N). **c** *p* = 0.0001 (WT D vs. WT N), *p* = 0.0005 (*Akt2* KI D vs. WT N), *p* = 0.0005 (*Akt2* KI D vs. *Akt2* KI N). **e** OPL, *p* = 0.0029 (WT D vs. WT N), *p* < 0.0001 (*Akt2* KI D vs. WT N), *p* < 0.0001 (*Akt2* KI D vs. *Akt2* KI N); INL, *p* = 0.0009 (WT D vs. WT N), *p* < 0.0001 (*Akt2* KI D vs. WT N, *Akt2* KI D vs. *Akt2* KI N); IPL, *p* = 0.0024 (WT D vs. WT N), *p* < 0.0001 (*Akt2* KI D vs. WT N, *Akt2* KI D vs. *Akt2* KI N). N nondiabetic, D diabetic, WT wild type, KI knock-in, IPL inner plexiform layer, INL inner nuclear layer, OPL outer plexiform layer.

Surprisingly, we observed no significant change in the c-wave (RPE function) between *Akt2* KI and WT diabetic mice (Fig. 5a–g), suggesting that RPE-specific overexpression of Akt2 had no effect on the diabetes-induced impairment of retinal function. In addition, the expression of inflammatory markers and leukostasis (Fig. 6a–f) and the levels of ROS production (Fig. 6g–k), were not different between KI and WT diabetic mice. Accordingly, the number of retinal acellular capillaries and capillary pericytes, the levels of retinal vascular leakage (Fig. 7a–e), as well as the infiltration of leukocytes (Supplementary Fig. 4) into the retina were similar in *Akt2* KI and WT diabetic mice.

### *Akt2* cKO promotes upregulation of Akt1 and prevents the diabetes-induced increased protein levels of ICAM-1 and iNOS in the RPE by mediating the GSK3β/NF-κB signaling pathway

**Fig. 8:**
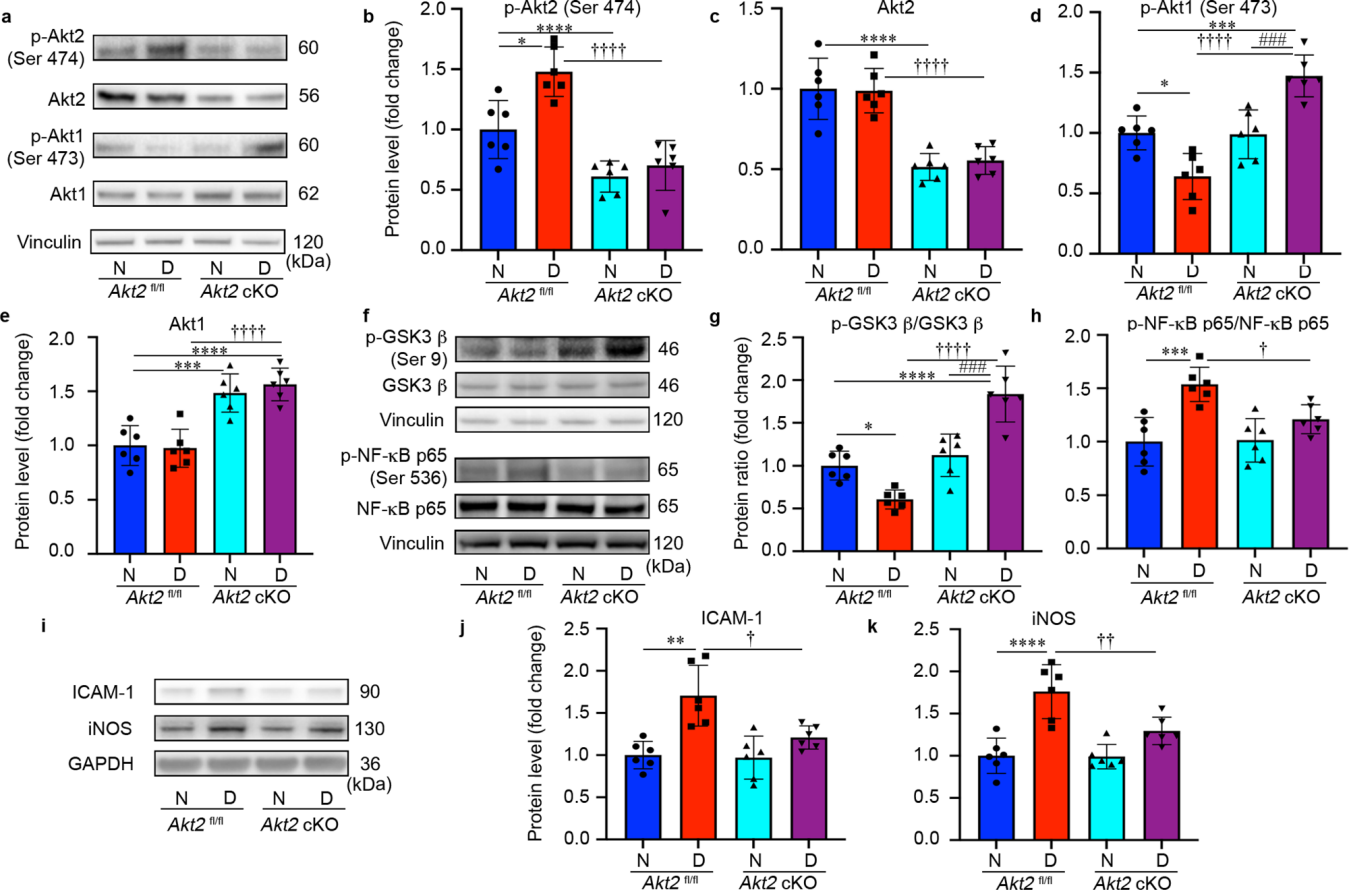
*Akt2* cKO promotes upregulation of Akt1 and inhibits the diabetes-induced inflammatory response through GSK3β/NF-κB signaling in the RPE cells (2 months of diabetes). **a** Representative immunoblots and quantification of (**b**) phospho-*Akt2*, (**c**) total-*Akt2*, (**d**) phospho-Akt1 and (**e**) total Akt1 in RPE lysates obtained from diabetic and nondiabetic *Akt2*^fl/fl^ and cKO mice. Diabetes increased the level of phospho-*Akt2* but did not affect the levels of total *Akt2* in *Akt2*^fl/fl^ RPE cells compared to the nondiabetic control. RPE-specific *Akt2* cKO reduced the levels of both phospho- and total-*Akt2* in the RPE; induction of diabetes did not further affect the levels of either phospho- or total *Akt2* in *Akt2* cKO RPE. Induction of diabetes in *Akt2*^fl/fl^ mice decreased the level of phospho-Akt1 but did not affect the levels of total Akt1 in the RPE cells compared to nondiabetic control. *Akt2* cKO increased the levels of phospho-Akt1 in diabetic *Akt2* cKO mice, but not in nondiabetic cKO mice compared to diabetic or nondiabetic *Akt2*^fl/fl^ mice, respectively. The total Akt1 in the RPE was upregulated in both nondiabetic and diabetic *Akt2* cKO mice compared to *Akt2*^fl/fl^ mice. **f** Representative immunoblots. **g** The ratio of phospho-GSK3β/total GSK3β was lower in diabetic *Akt2*^fl/fl^ mice, relative to nondiabetic control mice. However, it was increased in diabetic *Akt2* cKO mice. **h** In addition, *Akt2* cKO rescued the diabetes-induced increase in the ratio of p-NF-κB p65/total NF-κB p65. **i** Representative immunoblots. Diabetes induction increased the protein level of (**j**) ICAM-1 and (**k**) iNOS in *Akt2*^fl/fl^ mice, whereas in diabetic *Akt2* cKO mice this diabetes-induced increase in inflammatory protein levels was significantly rescued. In (**b**–**e**, **g**, **h**, **j**, **k**), *n* = 6 mice per group, the data are expressed as mean ± SD. *p < 0.05, **p < 0.01, ***p < 0.001, and ****p < 0.0001 denotes changes versus *Akt2*^fl/fl^ nondiabetic (N) control. ^†^*p* < 0.05, ^††^*p* < 0.01, ^†††^*p* < 0.001, and ^††††^*p* < 0.0001 denotes changes with respect to *Akt2*^fl/fl^ diabetic (D) mice. ^###^*p* < 0.001 and ^####^*p* < 0.0001 shows changes versus nondiabetic *Akt2* cKO group. Statistical test used in this study is One-way ANOVA followed by a Tukey’s post hoc test. Exact *p* values are: **b** *p* = 0.025 (*Akt2* ^fl/fl^ D vs. *Akt2* ^fl/fl^ N), *p* < 0.0001 (*Akt2* cKO N vs. *Akt2* ^fl/fl^ N*, Akt2* cKO D vs. *Akt2* ^fl/fl^ D). **c** *p* < 0.0001 (*Akt2* cKO N vs. *Akt2* ^fl/fl^ N, *Akt2* cKO D vs. *Akt2* ^fl/fl^ D). **d** *p* = 0.0109 (*Akt2* ^fl/fl^ D vs. *Akt2* ^fl/fl^ N), *p* = 0.0009 (*Akt2* cKO D vs. *Akt2* ^fl/fl^ N), *p* < 0.0001 (*Akt2* cKO D vs. *Akt2* ^fl/fl^ D), *p* = 0.0007 (*Akt2* cKO D vs. *Akt2* cKO N). **e** *p* = 0.0005 (*Akt2* cKO N vs. *Akt2* ^fl/fl^ N), *p* < 0.0001 (*Akt2* cKO D vs. *Akt2* ^fl/fl^ N). *p* < 0.0001 (*Akt2* cKO D vs. *Akt2* ^fl/fl^ D). **g** *p* = 0.0326 (*Akt2* ^fl/fl^ D vs. *Akt2* ^fl/fl^ N), *p* = 0.0001 (*Akt2* cKO D vs. *Akt2* cKO N), *p* < 0.0001 for the rest. **h** *p* = 0.0003 (*Akt2* ^fl/fl^ D vs. *Akt2* ^fl/fl^ N), *p* = 0.0283 (*Akt2* cKO D vs. *Akt2* ^fl/fl^ D). **j** *p* = 0.0004 (*Akt2* ^fl/fl^ D vs. *Akt2* ^fl/fl^ N), *p* = 0.011 (*Akt2* cKO D vs. *Akt2* ^fl/fl^ D). **k** *p* < 0.0001 (*Akt2* ^fl/fl^ D vs. *Akt2* ^fl/fl^ N), *p* = 0.0076 (*Akt2* cKO D vs. *Akt2* ^fl/fl^ D). Source Data is provided in the Source Data file.

To investigate further how the lack of Akt2 in the RPE protects the retina from diabetes-induced abnormalities, we focused on Akt1 and its downstream targets. We first analyzed the activity of Akt2 by evaluating the ratio of phospho-Akt2/total Akt2. We found that specific activity of Akt2 is increased while that of Akt1 is decreased in *Akt2*^fl/fl^ diabetic mice compared to *Akt2*^fl/fl^ nondiabetic controls. We also found that the induction of diabetes increased the level of phospho-Akt2 (active Akt2), but not total-Akt2 in *Akt2*^fl/fl^ diabetic mice compared to *Akt2*^fl/fl^ nondiabetic controls (Fig. 8a–c). As expected, *Akt2* cKO inhibited the diabetes-induced increase of phospho-Akt2 in the RPE (Fig. 8b). In contrast, the level of phospho-Akt1 was lower in the RPE of *Akt2*^fl/fl^ diabetic mice, while total Akt1 remained the same. Interestingly, both phospho- and total Akt1 were higher in *Akt2* cKO diabetic mice compared to *Akt2*^fl/fl^ nondiabetic mice (Fig. 8a, d, e). However, in *Akt2* cKO nondiabetic mice only total Akt1, and not the phospho-Akt1, was upregulated, compared to *Akt2*^fl/fl^ nondiabetic controls (Fig. 8a, d, e). These results suggest that diabetes is regulating phospho-Akt1 and Akt2 in a reciprocal manner.

To examine whether the diabetes-induced increase in the expression of pro-inflammatory proteins in the RPE is influenced by Akt1 downregulation, we evaluated the level of Akt1 downstream targets. Induction of diabetes decreased the ratio of phospho-GSK3β/GSK3β, and increased the proportion of phospho-NF-κB p65/NF-κB p65 in the RPE of *Akt2*^fl/fl^ diabetic mice compared to *Akt2*^fl/fl^ nondiabetic controls (Fig. 8f). *Akt2* cKO inhibited such diabetes-induced changes in the RPE, as supported by the comparison of *Akt2* cKO diabetic mice with *Akt2*^fl/fl^ diabetic controls (Fig. 8g, h).

It has been reported that GSK3β signaling is involved in the regulation of inflammation and leukocyte infiltration^61^. Thus, it is possible that *Akt2* knockout in the RPE has a protective effect against diabetes through upregulated Akt1, further inhibiting a GSK3β/NF-κB-regulated inflammatory response. To test this hypothesis, we looked at the level of downstream inflammatory proteins, ICAM-1 and iNOS in the RPE after 2 months of diabetes. Similar to the retina (Fig. 2b, c), the diabetes-induced increase in ICAM-1 and iNOS protein was inhibited in the RPE from *Akt2* cKO diabetic mice compared to the controls (Fig. 8i–k). This suggests that inhibition of diabetes-induced retinal molecular modifications and pathological changes in the RPE of *Akt2* cKO mice may be due to the upregulation of Akt1 and the downstream inhibition of the GSK3β/NF-κB regulated inflammatory response.

Both phospho- and total-Akt2 were increased in *Akt2* KI mice (both nondiabetic and diabetic) compared to WT nondiabetic controls. Similar to *Akt2*^fl/fl^ RPE, diabetes increased the level of phospho-Akt2, but not total Akt2, in WT mice (Supplementary Fig. 5a–c). Diabetes also reduced phospho-Akt1, but not total Akt1, in both WT diabetic and *Akt2* KI diabetic mice compared to corresponding nondiabetic controls, suggesting that *Akt2* KI had no effect on the diabetes-induced downregulation of phospho-Akt1 in the RPE (Supplementary Fig. 5d, e). Moreover, *Akt2* KI neither altered the phosphorylation of GSK3β or NF-κB (Supplementary Fig. 5f, g) nor affected the level of downstream inflammatory proteins in the RPE of *Akt2* KI diabetic mice (Supplementary Fig. 5h, i).

The inverse correlation between Akt2 and Akt1 was also confirmed by in vitro studies using *AKT2* siRNA. The protein levels of phospho- and total-AKT2 and AKT1 were assayed by western blotting in human fetal RPE (fRPE) cells cultured in presence of high glucose (25 mM) medium with or without *AKT2* siRNA (Supplementary Fig. 6a, b). Downregulation of AKT2 in fRPE cells markedly increased the ratio of phospho-AKT1/AKT1 without causing any significant change in the upstream regulators of AKT such as the ratios of phospho-PDK1/PDK1 and phospho-PI3K/PI3K (Supplementary Fig. 6b).

### Diabetes-induced activation of vasoactive mediators in retina/RPE is ameliorated by Akt2 knockdown and exacerbated by Akt1 inhibition

**Fig. 9:**
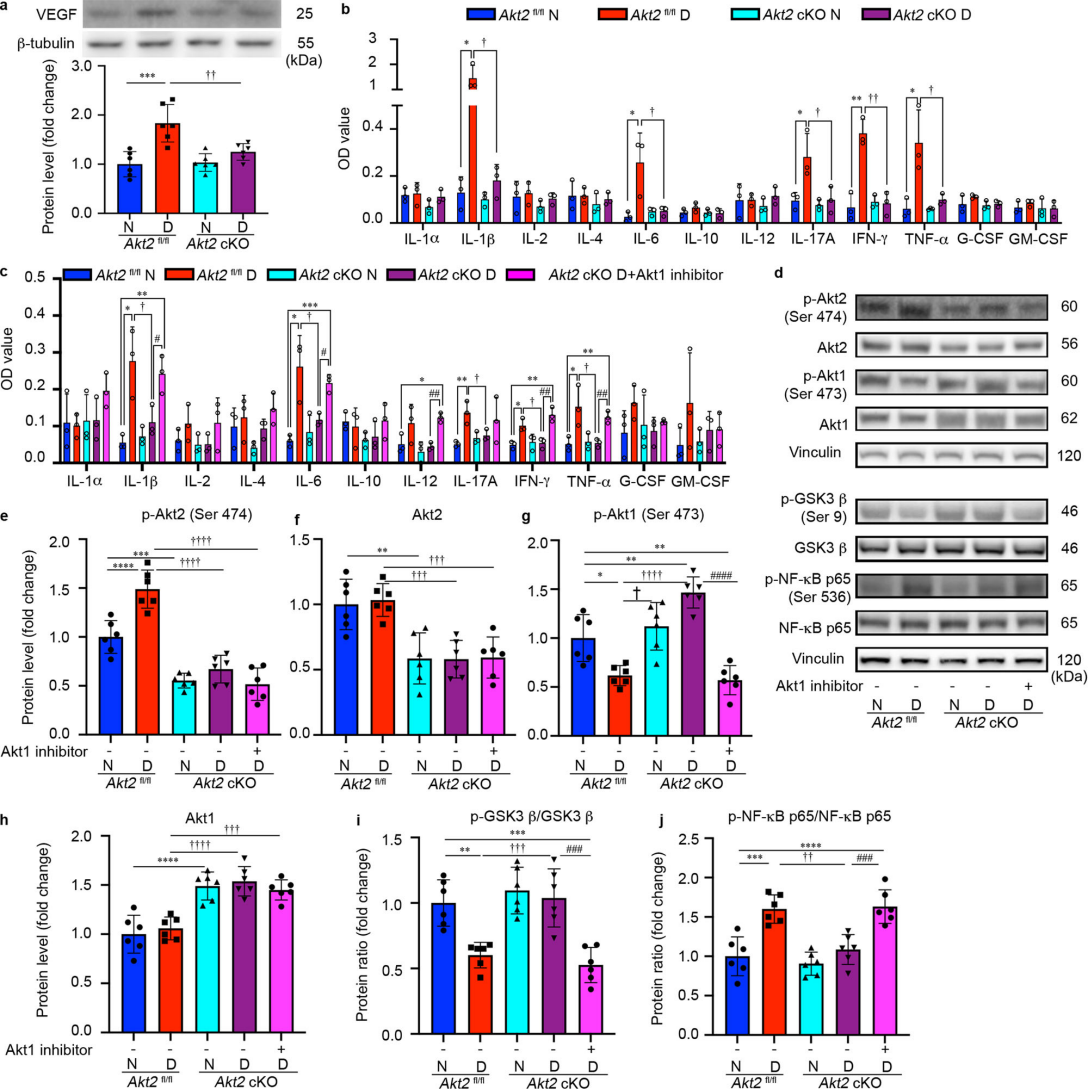
Inflammatory cytokines are elevated in the retina/RPE of *Akt2*^fl/fl^ diabetic mice, an effect inhibited in *Akt2* cKO mice; however inhibition of Akt1 reverses this protective effect. **a** Diabetes-induced increase of retinal VEGF was inhibited in *Akt2* cKO mice. **b** Diabetes increased the RPE/retinal expression of inflammatory cytokines, including IL-1β, IL-6, IL-17A, IFN-γ and TNF-α, in *Akt2*^fl/fl^ mice compared to *Akt2*^fl/fl^ nondiabetic animals. *Akt2* cKO inhibits diabetes-induced elevation of these inflammatory cytokines. **c** RPE explants were obtained from *Akt2*^fl/fl^ and *Akt2* cKO nondiabetic and diabetic mice and cultured in 5 mM and 25 mM glucose medium, respectively, for 48 h. The spent medium was collected and ELISA assays performed. As expected, *Akt2* cKO inhibited the release of inflammatory cytokines caused by diabetes, including IL-1β, IL-6, IL-12, IL-17A, IFN-γ and TNF-α. Such inhibitory effects were abolished if an Akt1 inhibitor was added to the RPE explants (isolated from *Akt2* cKO diabetic mice) culture. **d** RPE explant culture was collected for western blot analysis. **e**–**j** *Akt2* cKO increased the level of phospho-Akt1 in the RPE from diabetic mice and inhibited the diabetes-induced reduction in the ratio of p-GSK3β/total GSK3β, as well as the diabetes-induced elevation in the ratio of p-NF-κB p65/total NF-κB p65. The presence of the Akt1 inhibitor in the *Akt2* cKO diabetic mouse RPE explants significantly reversed this protective effect for the levels of p-Akt1, p-GSK3β/total GSK3β and p-NF-κB p65/total NF-κB p65. In (**a**–**c**, **e**–**j**), data are shown as Mean ± SD. *p < 0.05, **p < 0.01, ***p < 0.001, and ***p < 0.0001 versus the *Akt2*^fl/fl^ nondiabetic mice. ^†^*p* < 0.05, ^††^*p* < 0.01, ^†††^*p* < 0.001, and ^††††^*p* < 0.0001 versus *Akt2*^fl/fl^ diabetic mice. ^#^*p* < 0.05, ^##^*p* < 0.01, ^###^*p* < 0.001, and ^###^*p* < 0.0001 versus *Akt2* cKO diabetic group. Statistical tests used in (**a**, **e**–**j**) is One-way ANOVA followed by a Tukey’s post hoc test, *n* = 6 mice for each group. Statistical test used in (**b**, **c**) is two tailed, unpaired *t*-test, *n* = 6 animals; 3 samples in each group; each sample was composed of 2 animals. Exact *p* values are: **a** *p* = 0.0001 (*Akt2* ^fl/fl^ D vs. *Akt2* ^fl/fl^ N), *p* = 0.0049 (*Akt2* cKO D vs. *Akt2* ^fl/fl^ D), **b** IL-1β: *p* = 0.0342, 95% CI 0.4993 to 2.146, *R*^2^ = 0.8326 (*Akt2* ^fl/fl^ D vs. *Akt2* ^fl/fl^ N), *p* = 0.0128, 95% CI −2.095 to −0.4477, *R*^2.^ = 0.8212 (*Akt2* cKO D vs. *Akt2* ^fl/fl^ D); IL-6: *p* = 0.0352, 95% CI 0.026 to 0.4347, *R*^2^ = 0.7101 (*Akt2* ^fl/fl^ D vs. *Akt2* ^fl/fl^ N), *p* = 0.0483, 95% CI −0.4111 to −0.002517, *R*^2^ = 0.6639 (*Akt2* cKO D vs. *Akt2* ^fl/fl^ D); IL-17A: *p* = 0.0388, 95% CI 0.01557 to 0.3558, *R*^2^ = 0.6966 (*Akt2* ^fl/fl^ D vs. *Akt2* ^fl/fl^ N), *p* = 0.0374, 95% CI −0.3671 to −0.01823, *R*^2^ = 0.706 (*Akt2* cKO D vs. *Akt2* ^fl/fl^ D); IFN-γ: *p* = 0.0028, 95% CI 0.1819 to 0.4488, *R*^2^ = 0.915 (*Akt2* ^fl/fl^ D vs. *Akt2* ^fl/fl^ N), *p* = 0.0026, 95% CI −0.4227 to −0.1746, *R*^2^ = 0.9178 (*Akt2* cKO D vs. *Akt2* ^fl/fl^ D); TNF-α: *p* = 0.0291, 95% CI 0.04679 to 0.5145, *R*^2^ = 0.7351 (*Akt2* ^fl/fl^ D vs. *Akt2* ^fl/fl^ N), *p* = 0.0418, 95% CI −4694 to −0.01459, *R*^2^ = 0.6858 (*Akt2* cKO D vs. *Akt2* ^fl/fl^ D). **c** IL-1β: *p* = 0.0154, 95% CI 0.06971 to 0.373, *R*^2^ = 0.8042 (*Akt2* ^fl/fl^ D vs. *Akt2* ^fl/fl^ N), *p* = 0.048, 95% CI −0.3297 to −0.002349, *R*^2^ = 0.6684 (*Akt2* cKO D vs. *Akt2* ^fl/fl^ D), *p* = 0.0274, 95% CI 0.2393 to 0.2387, *R*^2^ = 0.7424 (*Akt2* cKO D + Akt1 inhibitor vs. *Akt2* cKO D), *p* = 0.0042, 95% CI 0.09867 to 0.2747, *R*^2^ = 0.8966 (*Akt2* cKO D + Akt1 inhibitor vs. *Akt2* ^fl/fl^ N); IL-6: *p* = 0.0154, 95% CI 0.06357 to 0.3391, *R*^2^ = 0.8045 (*Akt2* ^fl/fl^ D vs. *Akt2* ^fl/fl^ N), *p* = 0.044, 95% CI −0.2824 to −0.006241, *R*^2^ = 0.678 (*Akt2* cKO D vs. *Akt2* ^fl/fl^ D), *p* = 0.041, 95% CI 0.05295 to 0.1464, *R*^2^ = 0.8977 (*Akt2* cKO D + Akt1 inhibitor vs. *Akt2* cKO D), *p* = 0.007, 95% CI 0.1109 to 0.2024, *R*^2^ = 0.9576 (*Akt2* cKO D + Akt1 inhibitor vs. *Akt2* ^fl/fl^ N); IL-12: *p* = 0.0122, 95% CI 0.02614 to 0.1185, *R*^2^ = 0.8254 (*Akt2* cKO D + Akt1 inhibitor vs. *Akt2* ^fl/fl^ N), *p* = 0.0012, 95% CI 0.05197 to 0.1054, *R*^2^ = 0.9436 (*Akt2* cKO D + Akt1 inhibitor vs. *Akt2* cKO D); IL-17A: *p* = 0.0097, 95% CI 0.03451 to 0.1368, *R*^2^ = 0.8439 (*Akt2* ^fl/fl^ D vs. *Akt2* ^fl/fl^ N), *p* = 0.0358, 95% CI −0.1173 to −0.006687, *R*^2^ = 0.7077 (*Akt2* cKO D vs. *Akt2* ^fl/fl^ D); IFN-γ: *p* = 0.0114, 95% CI 0.0195 to 0.08512, *R*^2^ = 0.8308 (*Akt2* ^fl/fl^ D vs. *Akt2* ^fl/fl^ N), *p* = 0.0227, 95% CI −0.08379 to −0.01087, *R*^2^ = 0.7646 (*Akt2* cKO D vs. *Akt2* ^fl/fl^ D), *p* = 0.0055, 95% CI 0.03739 to 0.1153, *R*^2^ = 0.881 (*Akt2* cKO D + Akt1 inhibitor vs. *Akt2* cKO D), *p* = 0.0031, 95% CI 0.0458 to 0.1169, *R*^2^ = 0.9099 (*Akt2* cKO D + Akt1 inhibitor vs. *Akt2* ^fl/fl^ N); TNF-α: *p* = 0.0404, 95% CI 0.00717 to 0.1955, *R* ^2^ = 0.6909 (*Akt2* ^fl/fl^ D vs. *Akt2* ^fl/fl^ N), *p* = 0.0392, 95% CI −0.1894 to −0.007913, *R*^2^ = 0.6949 (*Akt2* cKO D vs. *Akt2* ^fl/fl^ D), *p* = 0.0038, 95% CI 0.03767 to 0.1017, *R*^2^ = 0.9014 (*Akt2* cKO D + Akt1 inhibitor vs. *Akt2* cKO D), *p* = 0.0078, 95% CI 0.03166 to 0.113, *R*^2^ = 0.8591 (*Akt2* cKO D + Akt1 inhibitor vs. *Akt2* ^fl/fl^ N). **e** *p* = 0.0003 (*Akt2* cKO N vs. *Akt2* ^fl/fl^ N), *p* < 0.0001 for the rest. **f** *p* = 0.0018 (*Akt2* cKO N vs. *Akt2* ^fl/fl^ N), *p* = 0.0016 (*Akt2* cKO D vs. *Akt2* ^fl/fl^ D), *p* = 0.0009 (*Akt2* cKO D + Akt1 inhibitor vs. *Akt2* ^fl/fl^ D). **g** *p* = 0.0124 (*Akt2* ^fl/fl^ D vs. *Akt2* ^fl/fl^ N), *p* = 0.0019 (*Akt2* cKO D vs. *Akt2* ^fl/fl^ N), *p* = 0.0043 (*Akt2* cKO D + Akt1 inhibitor vs. *Akt2* ^fl/fl^ N), *p* = 0.018 (*Akt2* cKO N vs. *Akt2* ^fl/fl^ D), *p* < 0.0001 for the rest. **h** p = 0.0007 (*Akt2* cKO D + Akt1 inhibitor vs. *Akt2* ^fl/fl^ N), *p* < 0.0001 for the rest. **i** *p* = 0.003 (*Akt2* ^fl/fl^ D vs. *Akt2* ^fl/fl^ N), *p* = 0.0004 (*Akt2* cKO D + Akt1 inhibitor vs. *Akt2* ^fl/fl^ N, *Akt2* cKO D vs. *Akt2* ^fl/fl^ D), *p* = 0.0002 (*Akt2* cKO D + Akt1 inhibitor vs. *Akt2* cKO D). **j** *p* = 0.0002 (*Akt2* ^fl/fl^ D vs. *Akt2* ^fl/fl^ N), *p* < 0.0001 (*Akt2* cKO D + Akt1 inhibitor vs. *Akt2* ^fl/fl^ N), *p* = 0.0012 (*Akt2* cKO D vs. *Akt2* ^fl/fl^ D), *p* = 0.0006 (*Akt2* cKO D + Akt1 inhibitor vs. *Akt2* cKO D). Source Data is provided in the Source Data file.

VEGF is a key vascular mediator in the pathogenesis of diabetic eye disease and the target of current therapies for patients with vision-threatening disease^5^. We therefore examined whether Akt2 expression in RPE cells influenced the level of VEGF in diabetic animals. The expression of VEGF in the retina was elevated in *Akt2*^fl/fl^ diabetic mice, but not in *Akt2* cKO diabetic mice (Fig. 9a). To examine whether Akt2 in RPE could influence more broadly the expression of vasoactive mediators and cytokines generated in the retina and RPE, we evaluated inflammatory cytokines by ELISA after 2 months of diabetes. The retinas and RPE collected from *Akt2*^fl/fl^ diabetic mice had higher IL-1β, IL-6, IL-17A, IFN-γ and TNF-α levels than the *Akt2*^fl/fl^ nondiabetic animals. This diabetes-induced increase of pro-inflammatory cytokines in the retina and RPE was essentially absent in the *Akt2* cKO mice (Fig. 9b). These results were corroborated in RPE explant cultures (Fig. 9c). We also observed that the inhibition of inflammatory cytokines in the RPE explants from *Akt2* cKO diabetic mice was largely abolished by adding an Akt1 inhibitor to the culture medium for 48 h (Fig. 9c). These data further support the hypothesis that knockout of *Akt2* in the RPE inhibits diabetes-induced inflammatory responses through the upregulation of Akt1.

To further interrogate the relationship between Akt2 and Akt1 in the diabetic RPE, Akt isoforms and their downstream target proteins were analyzed by western blots on extracts from cultured RPE flat-mounts (Fig. 9d). RPE flatmounts from diabetic (2 months of diabetes) and age-matched nondiabetic mice were cultured in 25 mM and 5 mM D-glucose medium, respectively. We found that in the *Akt2*^fl/fl^ samples, diabetes increased the level of phospho-Akt2 (but not total Akt2) and increased the ratio of phospho-NF-κB p65/total NF-κB p65, while decreasing phospho-Akt1 and the proportion of phospho-GSK3β/total GSK3β as compared to *Akt2*^fl/fl^ nondiabetic controls (Fig. 9e–j). *Akt2* cKO rescued these diabetes-induced molecular alterations. Importantly, a 48-h incubation of diabetic *Akt2* cKO RPE flatmounts with an Akt1 inhibitor reversed the protective effect of *Akt2* knockout in the RPE by increasing the ratio of phospho-NF-κB/NF-κB and decreasing the level of both phospho-Akt1 and the ratio of phospho-GSK3β/ GSK3β back to levels comparable to those seen in the diabetic *Akt2*^fl/fl^ RPE (Fig. 9e–j). These data suggest that decreasing Akt2 in the RPE inhibits diabetes-induced retinal abnormalities in mice, through the upregulation of Akt1, and, in turn inhibition of the GSK3β/NF-κB-regulated inflammatory response.

### Increased Akt1 signaling in the RPE prevents increased levels of inflammatory proteins and superoxide production in the diabetic retina; loss of Akt1 activity accelerates diabetes-induced retina vascular damage

These results suggest that the protective effect observed in RPE-specific *Akt2* cKO mice is due to the upregulation of Akt1. We therefore speculated that increasing Akt1 activity specifically in the RPE may serve as an effective intervention to treat DR. To test this, we overexpressed Akt1 in RPE by subretinal injection (SRi) of AAV2-hRPE-GFP-P2A-mAkt1 (once per month) in WT diabetic mice when diabetes induction was confirmed in these mice 7 days post STZ injection. After 2 months of treatment, we found decreased expression of the inflammatory proteins, iNOS and ICAM-1, as well as the ratio of pIκB/IκB in the retina of diabetic mice overexpressing Akt1 in their RPE, compared to diabetic mice treated with the control vector, AAV2-hRPE-EGFP (Supplementary Fig. 7a–f). Retinal superoxide production was also markedly reduced in these RPE-specific Akt1 overexpressing diabetic mice (Supplementary Fig. 7g). These data suggest that increasing Akt1 function specifically in the RPE protects against diabetes-induced inflammatory changes and oxidative stress in the retina.

To further confirm that decreased Akt1 activity in RPE is detrimental in DR, we administered an Akt1 inhibitor to diabetic mice by intravitreal injection (ITVi) or SRi. After 4 months of treatment (once per month), when mice were diabetic for 4 months (6 months of age), we evaluated retinal vascular damage, and found that diabetes had not produced significant retinal capillary degeneration or pericyte loss. However, in the presence of the Akt1 inhibitor in the retina and RPE, such vascular changes were exacerbated as evident from increased number of acellular capillaries and loss of pericytes in the retina (Supplementary Fig. 7h–j). This suggests that modulation of Akt1 in the retina/RPE may play an important role in diabetes-induced early retinal molecular alterations such as inflammation and oxidative stress and pathological changes such as capillary degeneration and pericyte loss.

### Diabetes-induced ROS regulates Akt2, but not Akt1 in the RPE through PI3K/PDK1 signaling

To explore how diabetes triggers reciprocal regulation of Akt1 and Akt2 in RPE, we investigated the upstream regulators of Akt and found that diabetes increased the ratio of p-PDK1/PDK1 and the ratio of p-PI3K/PI3K in RPE of 2 months diabetic mice compared with age-matched nondiabetic wild type controls. This correlated with the elevated ratio of p-Akt2/Akt2 (Supplementary Fig. 8a–c). We reported here that diabetes increases retinal production of ROS, which in turn, have been reported to activate Akt in RPE cells^62^. To confirm whether elevated p-Akt2/Akt2 ratio in the RPE is caused by ROS via the PI3K/PDK1 signaling pathway in diabetic animals, we isolated and cultured RPE explants from wild type diabetic and nondiabetic mice, with or without an antioxidant (N-acetylcysteine; NAC) or PI3K inhibitor (LY294002) for 24 h. Both NAC and LY294002 treatments lowered the phospho-Akt2/Akt2, phospho-PDK1/PDK1 and phospho-PI3K/PI3K ratios in the RPE explant lysates from diabetic mice in comparison to the untreated RPE-explants from diabetic mice. However, there was no significant change in phospho-Akt1/Akt1 after NAC and LY294002 treatments in comparison to control untreated RPE explants from diabetic mice (Supplementary Fig. 8d–h). These results suggest that the hyperglycemia-induced increase in Akt2 phosphorylation in the RPE is possibly regulated by ROS through the PI3K/PDK1 signaling, while the decreased Akt1 activity is not regulated by ROS and is independent of PI3K/PDK1 signaling.

### High glucose increased Akt2 phosphorylation and diminished Akt1 activity in the RPE by PI3K/PDK1 dependent and independent pathways

Consistent with our findings in the RPE of diabetic mice, reciprocal regulation of AKT1 and AKT2 phosphorylation in the RPE was observed in human diabetic retinopathy donor cadaver samples, compared to nondiabetic controls (Supplementary Fig. 9a, b). Basic characteristics of human RPE cadaver tissue donors are provided in Supplementary Table 2. To confirm these findings, human fRPE cells were cultured in low (5 mM) and high (25 mM) glucose medium for 1, 2 and 4 days. We found that after 1 day of high glucose exposure there was a substantial increase in Akt1 activity, as evident from the elevated ratio of p-AKT1/AKT1, compared to low glucose conditions. However, AKT1 activity declined after 2 days in high glucose culture conditions, and returned to near control levels by 4 days (Supplementary Fig. 9c, d). On the other hand, the high glucose treatment induced a sustained increase in AKT2 activity (ratio of phospho-AKT2/AKT2) as seen after 1, 2 and 4 days of incubation (Supplementary Fig. 9e). The change in AKT2 activity is coincident with increased phospho-PDK1/PDK1 and phospho-PI3K/PI3K ratios after cells were grown in a high glucose medium (25 mM D-glucose) for 2 and 4 days, suggesting that high glucose-induced elevation of AKT2 activity may be modulated via the PI3K/PDK1 signaling pathway, similar to our observations in the RPE cells of WT diabetic mice. These data confirm that AKT1 and AKT2 activities function inversely in the human RPE and particularly in DR donor cadaver RPE, and that high glucose may serve to increase AKT2 activity through PI3K/PDK1 signaling. We also found high glucose-induced alterations in phospho- and total AKT2, PDK1, and PI3K in human fRPE cells maintain their significance when compared to the osmotic controls (source data). Thus, the effects observed in this study are high glucose specific. The fPRE cells cultured in high glucose condition did not show decreased AKT1 activity as seen in the RPE of human DR donors. This suggest that the decreased AKT1 activity in RPE from human diabetic DR donors, similar to diabetic mice may be regulated by factors other than high glucose, and seemingly independent of PI3K/PKD1 signaling.

Previous studies have reported increased cellular (specifically mitochondrial) ceramide in RPE cells derived from diabetic patients^63^. An important ceramide metabolism dysregulation in the diabetic retina is the shift in the spectrum of sphingolipid forms from protective very long chain (VLC) ceramides (C ≥ 26) to pro-inflammatory and proapoptotic short-chain (SC) ceramides (C ≤ 24) which are mainly produced from sphingomyelins by acid sphingomyelinase (ASM)^64^. Of interest, ceramide has been shown to inhibit Akt1 in RPE independent of the PI3K signaling pathway^65^. To determine whether ceramide plays a role in the reduction in Akt1 activity, human fRPE cells were treated with C6-ceramide (H6524 Sigma-Aldrich) at concentrations of 2, 10, and 50 μM respectively under high glucose conditions (25 mM) for 4 days. We found that ceramide reduced Akt1 activity as indicated by reduced p-Akt1/Akt1 ratio at all three concentrations tested (Supplementary Fig. 9h, i). AKT2 activity was also decreased upon ceramide treatment but only at the higher concentrations, compared to cells without ceramide treatment (Supplementary Fig. 9j). There were no significant differences in the ratios of phospho-PDK1/PDK1 and phospho-PI3K/PI3K between ceramide-treated and untreated fRPE cells. These data suggest that the decreased AKT1 activity in the RPE observed in diabetes may be caused by ceramide, independent of PI3K/PKD1 signaling. To confirm the effect of ceramide in inhibiting AKT1 activity in RPE under high glucose conditions, we incubated human fRPE cells in high glucose medium with or without ASM inhibitor (desipramine; 2 μM). Our results showed elevated ASM mRNA levels in human fRPE cells cultured in 25 mM D-glucose medium for 4 days (Supplementary Fig. 9m), which is consistent with previous findings showing highly upregulated ASM expression in the diabetic retina^64,66^. We further found that high glucose significantly increased ceramide accumulation in the RPE cells, which was diminished by ASM inhibitor (Supplementary Fig. 9n, o). ASM-mediated decrease in endogenous ceramide was also found to be associated with increased phospho-AKT1/AKT1 ratio under high glucose conditions after 4 days (Supplementary Fig. 9q). However, the ratio of phospho-AKT2/AKT2 (Supplementary Fig. 9r), phospho-PDK1/PDK1 (Supplementary Fig. 9s) and phospho-PI3K/PI3K (Supplementary Fig. 9t) remained higher level in high glucose conditions without showing any significant difference between ASM inhibitor treated or untreated groups. We postulate that reducing ceramide by ASM inhibitor does affect AKT1 activity, at least under the specific concentration (desipramine, 2 μM) used in this study. Together with the C6-ceramide treatment study, these data suggest that the decreased AKT1 activity in the RPE observed in both diabetic mice and DR patients may be caused by increased short-chain ceramide which is possibly dependent on ASM activity, but independent of PI3K/PKD1 signaling.

## Discussion

DR is a multifactorial disease with limited therapeutic options for patients^5^. The few treatments that exist are aimed at preventing the progression of advanced-stage disease^5^. As a consequence, DR remains a tremendous burden on the quality of life of the diabetic population. Identifying new pathways that contribute to disease progression, particularly in early DR, is therefore crucial for improving vision in diabetic patients in the future.

Hyperglycemia is arguably the most important risk factor in the development of DR^67^. Nonetheless, strict control of glucose levels does not prevent disease progression in all patients^68^. Many patients have sustained disease progression even after glucose normalization, perhaps due to metabolic memory^69,70^. Anti-VEGF and laser photocoagulation treatments have been used as an essential clinical treatment for DR patients^5^. However, both can be associated with either unwanted side effects and/or inadequate response in a majority of patients^5^. Other contributors to DR pathogenesis include inflammation and oxidative stress^71^. Anti-inflammatory therapies such as high-dose aspirin treatment and germline deletion of iNOS inhibit retinal capillary degeneration in diabetic animals^52,72,73^. Likewise, antioxidant therapies such as vitamin C or vitamin E treatment and overexpression of superoxide dismutase have been shown to inhibit capillary degeneration in DR animal models^53,54^. While these therapies are being currently explored for the treatment of DR, to date no comprehensive solution to prevent/treat early DR exists. Thus, finding novel therapeutic approaches remains critically important.

The three Akt isoforms, Akt1, Akt2, Akt3 are functionally distinct. Amongst these, Akt2 has been shown to play a role in diabetes^38^. In particular, it has been reported that *Akt2* whole-body knockout mice display a severe type-II diabetes phenotype and that human patients with mutations in Akt2 show severe insulin resistance and develop diabetes^38,39^. In the retina, Akt2 was reported to be activated under sorbitol-induced hyperosmotic stress conditions, implicating Akt2 in DR pathogenesis, since elevated sorbitol in diabetic retinas is known to potentiate the pathogenesis of DR^40,74^. We report here that in RPE cells from both DR eyes and diabetic mice, that Akt2 activity is increased and that this increase is driven by ROS through PI3K/PDK1 signaling. Knocking out *Akt2* specifically in the RPE inhibited the diabetes-induced increases in the level of retinal inflammatory proteins, production of ROS, vascular leakage and retinal capillary degeneration. However, overexpression of Akt2 did not have the reverse effect, suggesting an indirect mechanism whereby loss of Akt2 expression in RPE influences DR progression.

Interestingly, the level of phospho-Akt1 was found to be decreased in the retinas of STZ-induced diabetic mice, supporting the involvement of Akt1 signaling in DR pathogenesis^75^. We found phospho-Akt1 is also decreased in RPE from human DR donors and experimental diabetic mice; such reduction in Akt1 activity was independent from PI3K/PDK1 signaling but dependent on the accumulation of ceramide in the RPE. Akt isoforms are known to have compensatory and complementary roles in diabetes^76^. Here, we demonstrate that knocking out *Akt2* specifically in the RPE upregulates total *Akt1* in both nondiabetic and diabetic RPE, while phospho-Akt1 was only upregulated in diabetic RPE, suggesting the involvement of a signaling cascade that regulates the phosphorylation status of Akt1. In line with these observations, it has previously been reported that knockdown of Akt1 promotes Akt2 upregulation in human lens epithelial cells, suggesting a similar compensatory interplay between these two isoforms in the lens^33^. Conversely, mice lacking Akt2 showed stress-induced photoreceptor degeneration that was not complemented by Akt1^32^. Collectively, these studies demonstrate the complex relationship between Akt isoforms in different tissues in ocular disease.

The mechanism underlying the compensation between the two Akt isoforms requires further investigation. However, our studies suggest that this compensation could play a critical role for the development of early DR. The induction of diabetes increased the levels of retinal inflammatory proteins and ROS production, ultimately leading to retinal capillary degeneration and vascular leakage in both WT and *Akt2*^fl/fl^ mice. RPE-specific *Akt2* cKO successfully inhibited these diabetes-induced retinal abnormalities, supporting a role for the RPE in the development of DR and suggesting that Akt signaling in the RPE may be involved in maintaining retinal homeostasis. Since inflammation plays a detrimental role in the progression of DR^77^, and Akt1 is known to regulate inflammation^61^, we propose that the protective effect observed in the RPE-specific *Akt2* cKO diabetic mice could be mediated by regulation of inflammation by the compensatory increase in Akt1.

**Fig. 10:**
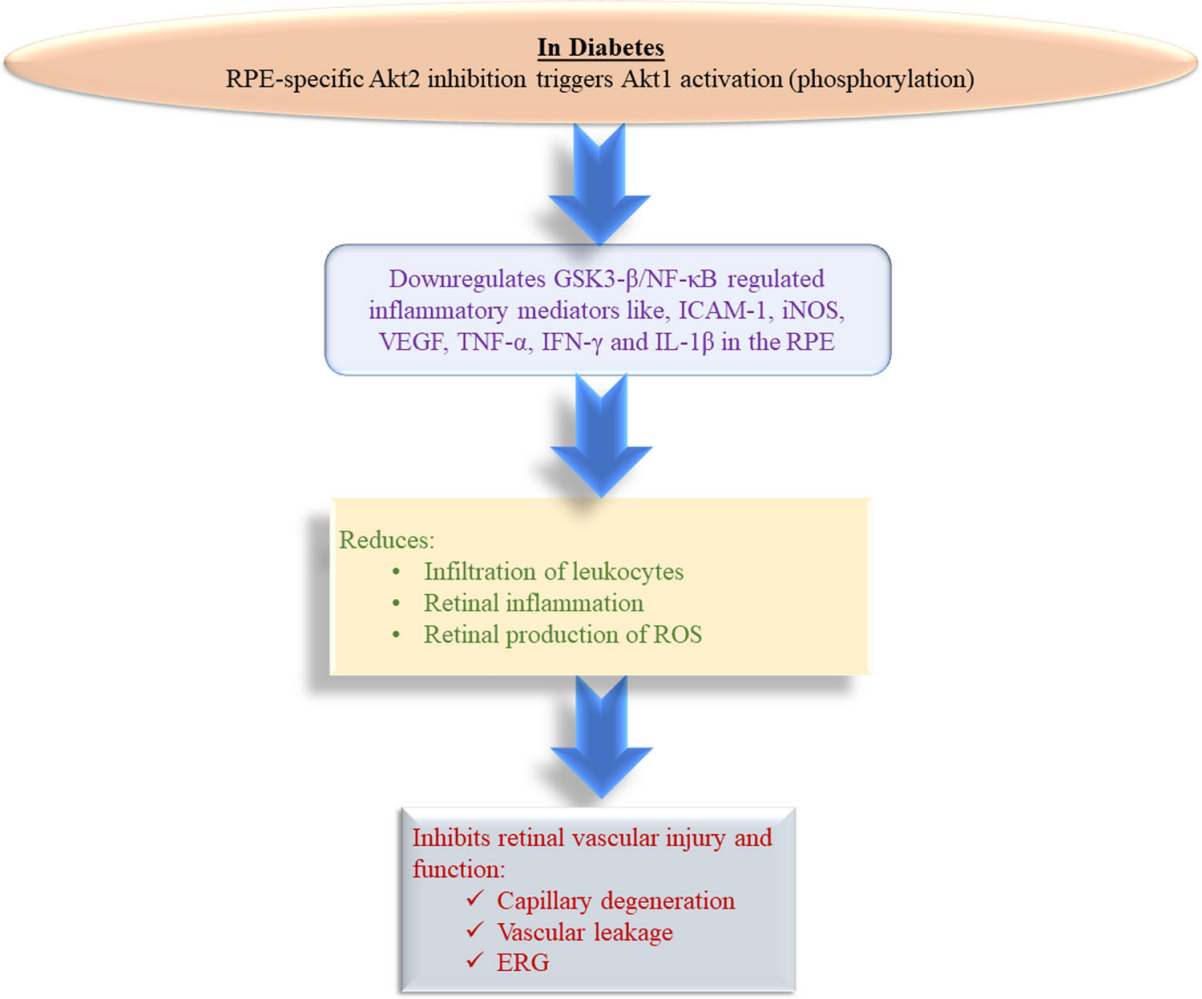
Schematic. RPE-specific *Akt2* cKO reduced the diabetes-mediated induction of the inflammatory protein level, infiltration of leukocytes and production of ROS in the retina after a 2 month duration of diabetes. *Akt2* cKO also showed reduction in retinal capillary degeneration and vascular leakage even after an 8 month duration of diabetes. Such beneficial effects were indicative of the compensatory upregulation of Akt1 in the diabetic *Akt2* cKO RPE cells, which downregulated GSK3β/NF-κB mediated inflammatory molecules like ICAM-1, iNOS, VEGF, TNF-α, IFN-γ and IL-1β in cKO RPE cells even after diabetes induction.

Akt1 is a multifunctional kinase that can phosphorylate multiple targets^78^. We have found that phospho-GSK3β, a critical Akt1 downstream effector known to regulate inflammatory responses, including immune cell infiltration and activation^61^, was also increased concomitantly in the RPE. Importantly, GSK3β is known to control activation of NF-κB, which has been described as a promising target in the management of vascular complications of diabetes, including DR^61,79^. Our data support the premise that *Akt2* cKO in the RPE has a protective effect against diabetes-induced retinal damage, including infiltration and activation of immune cells, by mediating the Akt1/GSK3β/NF-κB signaling axis. The premise is further supported by the observation that *Akt2* KI showed no protective effect in the diabetic retina because the overexpression of Akt2 did not affect the level of phospho-Akt1 in the RPE. Our studies further suggest that targeting Akt1 in RPE could be an effective approach to treat DR. Herein we show that increasing Akt1 activity in retina/RPE attenuated diabetes-induced retinal molecular alterations and that reduction of Akt1 activity in RPE accelerated retinal pathological vascular damage in diabetic mice. Collectively, our results expose the complex interplay between Akt isoforms and a compensatory mechanism in Akt signaling in the RPE during the progression of DR and identify a pathway that could be manipulated to develop new therapies for the treatment of diabetic eye disease (Fig. 10).

## Methods

### Animals

Wild type C57BL/6J mice were purchased from The Jackson Laboratory (Stock number: 000664, Bar Harbor, ME). RPE-specific *Akt2* KI mice were engineered by Cyagen (Santa Clara, CA). Briefly, the strategy is based on the insertion of the T2A sequence followed by the *Akt2* coding sequence (CDS) between the last exon and the 3′ untranslated region (3′UTR) of the mouse *Best1* gene. The T2A sequence enables the co-expression of both sequential genes (in this case, *Best1* and *Akt2*) from the same Best1 promoter, which is specific to the RPE. The Neo^r^ cassette flanked with self-deletion anchors (SDA) was inserted in the intron area between exons 11 and 12 of the mouse *Best1* to be deleted in germ cells. RPE-specific *Akt2* cKO mice were generated by mating the *Akt2*^fl/fl^ mice (The Jackson Laboratory, Stock number: 026475) with *Best1/Cre* mice (The Jackson Laboratory, Stock number: 017557) followed by cross-mating the progeny to generate the Cre-expressing mice homozygous for the *Akt2*^fl/fl^. The *Akt2*^fl/fl^ mice possess loxP sites flanking exons 4 and 5 of *Akt2*; the *Best1/Cre* mice express Cre recombinase under the control of the Best1 promoter. Thus, mating these two strains results in progeny, where exons 4 and 5 of *Akt2* are excised by Cre recombinase controlled by the Best1 promoter, making the resulting Akt2 protein dysfunctional only in the RPE. All animals including male and female were housed in ventilated microisolator cages under a 12/12 h of light and dark cycle. The temperature and humidity are controlled. All animal studies were conducted in accordance with the guide for the care and use of animals (National Academy Press) and were approved by the Institutional Animal Care and Use Committee of the University of Pittsburgh. Protocol number is 20108281.

### Induction of diabetes

Male mice around 2 months of age were intraperitoneally injected with streptozotocin (STZ, Sigma-Aldrich, S0130) suspended in citrate buffer at a concentration of 60 mg/kg for five consecutive days. Non-fasting blood glucose was measured at 10am on days 7–15 after the last STZ injection. The onset of diabetes was defined as blood glucose readings exceeding 275 mg/dL on three different days. The number of months of diabetes described in the text is defined as the age of the mice at the time of sacrifice minus the age when they were first confirmed to be diabetic. To assess the severity of hyperglycemia or diabetic status, HbA1c was measured using the Mouse HbA1c Assay Kit (Crystal Chem, 80310) and its control (Crystal Chem, 80313). Body weight was monitored within 2–3 weeks after the injection of STZ. To prevent weight loss while still allowing hyperglycemia, subcutaneous injection of insulin (0–0.2 units, Invitrogen, 12585014) was administered as needed.

### Western blotting

RPE and retina lysates were prepared using RIPA lysis buffer^80^. Briefly, the mouse was euthanized by CO_2_ asphyxiation, the fresh isolated RPE/Choroid/Sclera complexes and retina were placed into RIPA lysis buffer (Millipore, 20–188) separately. RPE cells were released into RIPA buffer from the RPE/Choroid/Sclera complexes by tapping for 30 s, Choroid/Sclera complexes were then removed by forceps. The human fetal RPE cells derived from healthy human RPE cells and are primary cells that retain many in vivo phenotypic characteristics. RPE-specific markers such as RPE65 and epithelial markers ZO1 are expressed in the human fetal RPE cells. The human fRPE cells cultured in 5 mM, 25 mM D-glucose, along with 25 mM mannitol (Sigma-Aldrich, M4125) as the osmotic control, were first washed with PBS, followed by cell lysate preparation with RIPA lysis buffer in the 6-well culture plates. fRPE cell lysates were then collected into 1.5 tubes. All samples were sonicated in RIPA lysis buffer with 1% phosphatase (Sigma-Aldrich, P0044,) and protease (Sigma-Aldrich, I3786) inhibitors, then centrifuged at 13,000 g for 20 min at 4 °C. The supernatants were collected, and protein concentrations were estimated by a BCA kit (Thermo Fisher, 23225). Samples were adjusted to the same concentration by adding lysis buffer and were then mixed with 4X protein sample buffer (NP0007, Life Technologies) with 5% 2-mercaptoethanol (Sigma-Aldrich, M3148) and heated at 96 °C for 10 min. Total protein (10 μg for RPE and human fRPE, 20 μg for retina) was loaded into 4–12% Bis-Tris Nu-PAGE gels (Thermo Fisher Scientific, NW04125BOX), proteins were transferred onto nitrocellulose membranes after electrophoreses were performed in MES buffer (Thermo Fisher Scientific, B0002), and the membrane was blocked with 5% milk (Bio-Rad, 170–6404) for an hour, followed by primary antibodies overnight. Unless otherwise stated, primary antibody was used at a dilution of 1:1000. Primary antibodies are as follows: iNOS (Cell Signaling Technology, 2982S), ICAM-1 (Proteintech, 10020-1-AP), Phospho-IκBα (Ser32) (1:500 dilution, Cell Signaling Technology, 2859T, Clone: 14D4), IκBα (Cell Signaling Technology, 4812S, Clone: 44D4), Phospho-Akt2 (Ser474) (Cell Signaling Technology, 8599S, Clone: D3H2), Akt2 (Cell Signaling Technology, 3063 S, Clone: D6G4), Phospho-Akt1 (Ser473) (Cell Signaling Technology, 9018S, Clone: D7F10), Akt1 (Cell Signaling Technology, 2938S, Clone: C73H10), Phospho-NF-κB p65 (Ser536) (Thermo Fisher Scientific, MA515160, Clone: T.849.2), NF-κB p65 (Thermo Fisher Scientific, 10745-1-AP), Phospho-GSK-3β (Ser9) (Cell Signaling Technology, 5558S, Clone: D85E12), GSK-3β (Thermo Fisher Scientific, 85-86173-11), PDK1 (Cell Signaling Technology, 3062), phospho-PDK1 (1:500 dilution, Cell Signaling Technology, 3438, Clone: C49H12), PI3K (Cell Signaling Technology, 4257, Clone: 19H8), phospho-PI3K (1:500 dilution, Cell Signaling Technology, 4228), GAPDH (Cell Signaling Technology, 5174S, Clone: D16H11), and Vinculin (Abcam, ab129002, Clone: EPR8185). RPE65 (Invitrogen, MA-532633, Clone: JM61-51) and rhodopsin (Abcam, ab98887, Clone: Rho 4D2) were used to validate potential RPE and retina contamination in the mouse samples. The membranes were washed three times with TBS (Fisher Scientific, 351-086-131) with 0.1% Tween (Sigma Aldrich, P7949) for 10 min per wash and incubated with appropriate peroxidase-labeled goat anti-rabbit secondary antibody (SeraCare, 5220-0336) at a dilution of 1:2000 for an hour at room temperature, followed by another three washes in TBS-Tween. The membranes were then developed using the ECL Western Blotting Detection Reagent (GE Healthcare, RPN2209) and the Azure c400 system.

### RPE flatmount and retinal section immunostaining

RPE-choroid flatmounts and retina sections were first permeabilized with 0.25% Triton X-100 in PBS for 10 min, followed by 2% donkey serum, 2% goat serum, 1% BSA, and 0.1% Triton X-100 in PBS (blocking buffer) for 30 min at room temperature. *Akt2*^fl/fl^ and *Akt2* cKO RPE flatmounts were incubated with Cre antibody (1:200, MilliporeSigma, MAB3120, Clone: 2D8), while WT and *Akt2* KI RPE flatmounts were incubated with Best1 antibody (1:200, Bioss Antibodies, bs11040R,) in blocking buffer at 4 °C overnight. Retinal sections were incubated with rhodopsin (1:200, Abcam, ab98887) and opsin (1:200, MilliporeSigma, AB5405) in blocking buffer at 4 °C overnight. RPE flatmounts and retinal sections were washed in 1X PBS for 3 times, 5 min per wash. The secondary antibody Donkey anti-Rabbit, Alexa Fluor 488 (1:200, Invitrogen, A21206), Donkey anti-mouse, Alexa Fluor 488 (1:200, Invitrogen, A21202), Goat anti-Rabbit, Alexa Fluor 568 (1:200, Invitrogen, A11011), 1 μg/mL DAPI (1:400, Thermo Fisher, D1306), and Alexa Fluor 594 conjugated ZO-1 (1:200, Invitrogen, 339194) antibody were applied to appropriate samples for 1 h incubation at room temperature. RPE flatmounts and retinal sections were cover slipped with DAKO mounting medium (Agilent, S3023). Images were acquired by Zeiss LSM 710 confocal microscopy.

### Leukostasis

The animals were anesthetized and the chest cavity was cut open to expose the heart. The mice were perfused with pre-warmed saline buffer for two min to remove free blood, followed by 10 ml of diluted concanavalin A-FITC solution (1:50, Vector Laboratories, FL-1001) for 1 min, followed by an additional 2 min of saline^58,81^. Then, the eyes were enucleated, retinas were isolated and flat-mounted on a slide, and the FITC-labeled leukocytes in blood vessels of the whole retina were counted using a Zeiss LSM 710 confocal microscope (Carl Zeiss Meditec, Jena, Germany).

### Superoxide and reactive oxygen species

Superoxide levels were assessed by lucigenin (bis-N-methylacridinium nitrate)^42^. Freshly isolated retinas were incubated in 200 μl Krebs-Hepes buffer with 5 mM or 25 mM glucose (Sigma-Aldrich, G8644) for 5 min at 37 °C in 5% CO_2_. Lucigenin (Sigma-Aldrich, M8010) was added to each sample to a final concentration of 0.54 mM and incubated for 5 min. The luminescence was measured by a Glomax 20/20 luminometer (Promega, USA) and reported in arbitrary units per mg of retinal protein.

Histochemical assays for ROS and superoxide levels were conducted by staining unfixed retinal cryosections with 2′,7′-Dichlorofluorescin diacetate (DCF, Sigma-Aldrich, D6883) and Dihydroethidium (DHE, Fisher Scientific D11347). Mouse eyes were freshly collected, embedded in OCT compound (Fisher Scientific, NC1029572 T), and frozen immediately on dry ice. Samples were cut into sections at a thickness of 12 μm and kept frozen during sectioning. Slides were immersed into ice-cold acetone for 10 min at −20 °C, warmed to room temperature for 20 min, and washed in PBS 3 times for 5 min per wash. Slides were then incubated with DCF 10 μM or DHE 0.625 μM for 60 min and 20 min, respectively, at room temperature in the dark. Sections were washed with PBS 3 times for 5 min, covered with coverslips using DAPI Fluoromount-G (SouthernBiotech, 0100-20) reagent, and photographed using confocal microscopy (Zeiss LSM 710). DCF green fluorescence intensity was measured at the photoreceptors’ inner and outer segments. DHE red fluorescence intensity was quantified by the nuclei of the ONL and INL layers because DHE stains DNA red in the presence of superoxide.

### Acellular capillaries

Mouse eyes were collected from diabetic (8 months of diabetes) and age-matched nondiabetic mice; samples were fixed in 4% buffered paraformaldehyde (Alfa Aesar, J61899-AP) for 2 days at room temperature. Retinas were isolated and washed overnight in water and incubated in elastase (EMD Millipore, 324682) solution 40 unit/ml at 37 °C for 2 h, followed by incubation in Tris buffer pH 8.5 overnight at room temperature. Retinal neurons were washed and brushed away from the vasculature. The carefully isolated vasculature was placed on a glass slide and dried for 24 h; samples were then stained with hematoxylin (Fisher Scientific, SH26-500D) and periodic acid Schiff reagent (Fisher Scientific, SS32-500). Acellular capillaries reported per square millimeter of retinal area were identified as capillary-sized vessel tubes with no nuclei along their length. Six field areas were counted per retina corresponding to the mid-retina^42^.

### Leakage of albumin into neural retina

Diabetes was induced at 2 months of age, and the mice were sacrificed after an 8 month duration of induction. The leakage of albumin into the neural retina was measured^23,82^. Sterile FITC-BSA (Sigma-Aldrich, A9771) was prepared in PBS at a concentration of 50 µg/µL, then injected into the mouse tail vein at 100 µg/g body weight and allowed to circulate for 20 min. Mice were then euthanized and blood samples collected and centrifuged at 2000 g for 10 min. 200μl of 1:1000 PBS-diluted plasma was transferred into 96 well plates, and the FITC-BSA concentration was measured using a Synergy microplate reader (BioTek), based on standard curves of FITC-BSA in normal mouse plasma with excitation at 433 nm and emission at 455 nm. Eyeballs were fixed in ice-cold 4% paraformaldehyde by immersion for 1 h. The eye from each mouse was incubated in a 5% to 20% sucrose gradient for at least 2 h each and then frozen in OCT compound (Fisher Scientific, NC1029572 T). When processing the frozen sections (11 nm thickness), the slides were thawed to room temperature, air-dried for 30 min under the hood, and rehydrated in PBS for 10 min. The mounting medium (SouthernBiotech, 0100-20) was added, and the slide was covered with a cover glass. The images were taken under green (FITC) light (2 noncontiguous cryosections/eye/animal) with ×40 magnification on both left and right sides of sections close to the optic nerve head region (300 μm distance from optic nerve head on each side). Average image pixel density in the neural retina was analyzed using ImageJ (version 1.51). Circles were drawn in the inner and outer plexiform layers, as well as in the INL of the neural retina (visible blood vessels were excluded) and a FITC-free area outside the retina for background subtraction. The retina mean fluorescence intensity was normalized to the relative FITC-BSA concentration of blood plasma from each animal. Vascular permeability was expressed as fold change of normalized fluorescence intensity in the neural retina relative to that in appropriate nondiabetic animals.

The retinal vascular permeability in mice was also assessed by tail vein injection of FITC-BSA at a dose of 200 µg/g body weight. After 2 h^83^, mice were euthanized and blood was drawn from the heart and centrifuged at 2000 × *g* for 10 min to collect the plasma. The animals were then perfused with warm saline through the heart for 2 min. The retinas were harvested and dried overnight to obtain dry weights. FITC-BSA was extracted from retinal tissues using 1% Triton X-100 in PBS. Blood and retina FITC-BSA concentrations were determined by a BioTek Synergy H1 Hybrid reader based on standard curves of FITC-BSA in normal mouse plasma. The retinal FITC-BSA value was normalized to the plasma FITC-BSA concentration, retinal dry weight and time of dye circulation.

### Ultrahigh-resolution spectral-domain optical coherence tomography (SD-OCT) imaging

Diabetic (8 month duration of diabetes) and age-matched control mice were anesthetized with ketamine (87.5 mg/kg) and xylazine (12.5 mg/kg). The pupils were dilated with 0.5% tropicamide (Medline, 17478-0101-12). Two images were acquired using SD-OCT (Bioptigen, USA) and averaged, and the thickness of the retinal ONL was measured at distances of 150, 300, and 450 μm from the optic nerve. The entire retina thickness was measured at distances of 300 μm from the optic nerve.

### Electroretinography (ERG)

ERGs were performed 4 months after the induction of diabetes (6 months of age). After overnight dark adaptation, mice were anesthetized with ketamine (87.5 mg/kg) and xylazine (12.5 mg/kg), the cornea was anesthetized with 0.5% proparacaine hydrochloride (Medline, 17478-0263-12), and the pupils were dilated with 2.5% phenylephrine hydrochloride (Medline, 17478-0201-15). Briefly, mice were placed on the temperature-regulated heated ERG instrument (Diagnosys, Lowell, MA), and the retinal responses recorded by placing electrode on each cornea; the subdermal reference was placed on the nose, and ground needle electrodes were placed at the base of the tail^56^. The full-field flash ERG responses from both eyes were recorded on mice dark-adapted overnight followed by light-adaptation (10 min of 30 cd/m2 light); the flash intensities were 0.01, 0.1, 0, 1, and 10 cd*s/m^2^ with a 2- to 5-s interstimulus interval. The amplitude of ERG a-wave was measured from the trough of the first corneal negative deflection to the pre-stimulus baseline. The amplitude of the b-wave was calculated from the a-wave amplitude to the peak of the b-wave. The c-wave was measured in response to 2.5 cd*s/m^2^ stimuli immediately before light adaptation. All ERG a-, b-, and c-wave amplitudes were automatically calculated by Espion V6 software (Diagnosys LLC).

### RPE explant culture

RPE explants from 2 months diabetic mice and age-matched nondiabetic *Akt2*^fl/fl^ and *Akt2* cKO mice were flat-mounted on polyvinylidene fluoride (PVDF) transfer membranes (Thermo Fisher, 22860) and cultured in Dulbecco’s modified Eagle’s medium (DMEM) medium (Thermo Fisher, 12320032) in 24-well plates for 48 h. RPE flatmounts from nondiabetic mice were cultured in DMEM medium containing 5.5 mM D-glucose (Sigma Aldrich, G8644), 2% FBS (Thermo Fisher, 10082147), while RPE flatmounts isolated from diabetic mice were cultured in the medium containing 25 mM D-glucose and 2% FBS. NAC (500 μM; dissolved in DMSO), and LY294002 (10 μM; dissolved in DMSO) were used in RPE explant treatment, in this case RPE flatmounts were cultured in medium containing 0.05% DMSO (vehicle control).

### ELISA

The Multi-Analyte ELISArray Kit (33616, QIAGEN) was used to measure the levels of mouse retinal inflammatory cytokines. The RPE with retina tissue was isolated from *Akt2*^fl/fl^ and *Akt2* cKO diabetic (2 months of diabetes) and nondiabetic control mice. Tissue was homogenized in 300 μl of complete extraction buffer (Abcam, ab193970). The supernatant was collected from the lysate by centrifuging it at 1000 × *g* for 10 min at 4 °C. The detailed procedure followed the manufacturer’s instructions. The absorbance was measured at 450 nm using a microplate reader. RPE-choroid was isolated from the same groups in the RPE flatmount culture experiment as described above. The RPE flatmounts were cultured in DMEM (Thermo Fisher Scientific, 12320032) with 2% FBS for 48 h. The culture medium with 5 mM D-glucose was used for nondiabetic RPE, and 25 mM D-glucose was used for diabetic mouse RPE flat-mount culture. An Akt1 inhibitor (Selleck Chemicals, S2670) was dissolved in sterile H_2_O at a stock concentration of 10 mM, and *Akt2* cKO diabetic RPE flatmounts were treated with the Akt1 inhibitor at a final concentration of 40 nM for 48 h. The flatmount culture medium was then collected, and the same Multi-Analyte ELISArray Kit mentioned above was used to measure the inflammatory cytokines, while RPE flatmount tissue was lysed for western blot analysis.

### Flow cytometry

For quantification of immune cells in the mouse RPE and retina, mouse retina and RPE-choroid were gently dissected and digested with 1.5 mg/ml collagenase A (Sigma-Aldrich, 10103578001) and 0.4 mg/ml DNase 1 (Sigma-Aldrich, 11284932001) at 37 °C for 1 h. Single-cell suspensions were generated by pipetting the tissue to release cells and passing cells through 70 μm filters. Cells were blocked with 1% mouse serum (Thermo Fisher Scientific, 10410) and 1 μg/μl Fc blocker (BD Biosciences, 553142), followed by antibody staining including BV650 Rat Anti-Mouse CD11b, (BD Biosciences, 563402, M1/70), PE-Cy7 Rat anti-Mouse CD45 (BD Biosciences, 561868), FITC Rat anti-Mouse CCR2 (SC203G11) (BioLegend, 150607), APC Rat Anti-Mouse Ly-6G (BD Biosciences, 560599, 1A8) and BV421 Rat Anti-Mouse Ly-6C (BD Biosciences, 562727, Al-21) in brilliant stain buffer (BD Biosciences, 563794,) at a concentration of 1 μg/mL for 30 min at room temperature. Flow cytometry was used to estimate the number of immune cells in the RPE/retina.

### siRNA transfection

The signal silence control siRNA (6568, Cell Signaling Technology) and *AKT2* siRNA (6396, Cell Signaling Technology) were commercially obtained. The siRNAs were transfected into RPE cells using Lipofectamine 3000 (L3000008, Thermo Fisher Scientific) following the manufacturer’s protocol. The silencing efficiency was detected by western blotting 48 h after transfection.

### Immunocytochemistry

The human fetal RPE cells derived from healthy human RPE cells, and were a gift from Dr. Ram Kannan. Informed consent was obtained for their use. Human fRPE cells were first seeded on cell culture cover glasses (Fisher Scientific, NC0620709) in a 24-well plate. On the second day, ASM inhibitor desipramine hydrochloride (2 μM, D3900, Sigma-Aldrich) was added and the cells were cultured in 5 mM, 25 mM D-glucose, and 25 mM mannitol medium for four additional days. Then, each well was washed with PBS followed by 2% PFA for 15 min at room temperature. The cells were then permeabilized with 0.1% Triton X-100 in PBS for 20 min, followed by 2% BSA, 2% donkey serum, and 0.05% Triton X-100 in PBS for 1 h at room temperature. Next, the samples were incubated at 4 °C overnight with an anti-ceramide antibody, (Sigma, C8140, 15B4) at a 1:200 dilution. After three washes with PBS, the cells were incubated with AF488 conjugated donkey anti-mouse secondary antibody at a 1:200 dilution and DAPI at a 1:600 dilution for 1 h at room temperature. Following incubation, the cover slides were removed from the 24-well plate, and the antifade medium was applied. The cells were then washed six times with PBS and placed on a slide. Finally, five images were acquired by Zeiss LSM 710 confocal microscopy at different fields of view for each sample to quantify the fluorescence intensity. The average fluorescence intensity of each cell was normalized to a control group where human fRPE were grown in a 5 mM glucose medium. The experiments were repeated four times.

### Quantitative real-time polymerase chain reaction

According to the manufacturer’s instructions, total RNA was extracted from human fRPE cells grown in 5 mM, 25 mM D-glucose, and 25 mM mannitol medium using an Isolate II RNA Mini Kit (BIO-52072, Bioline), and cDNA was generated using a SuperScript® VILO™ cDNA Synthesis Kit (11754-050, Invitrogen). Human ASM: Fwd: 5′-CAA CCT CGG GCT GAA GAA-3′; Human ASM: Rev: 5′-TCC ACC ATG TCA TCC TCA AAA-3′. Human Cyclophilin: Fwd: 5’-CAA GAC TGA GTG GTT GGA TGG-3′; and Human Cyclophilin: Rev: 5’-TGG TGA TCT TCT TGC TGG TCT-3′ ordered from Integrated DNA Technologies were mixed with Applied Biosystems SYBR Green Master Mix (A25742, Thermo Fisher Scientific). The mRNA expression of each target was evaluated using a QuantStudio 3 qPCR machine (A28131, Applied Biosystems by Thermo Fisher Scientific). Then, the gene expression levels were normalized relative to Cyclophilin mRNA and reported as fold change over controls using the Delta-Delta Ct method.

### Statistical analysis

Statistical analysis was performed using GraphPad 9.0 software (GraphPad Software, Inc., La Jolla, CA, USA) and Excel (Microsoft, version 16.54). All data were expressed as mean ± SD. Two-tailed, unpaired *t*-test was used to evaluate the mouse retina/RPE expression of inflammatory cytokines. To evaluate the significant differences for the rest of the data, One-way ANOVA followed by Tukey’s post hoc test was used for multiple-group comparisons.

### Reporting summary

Further information on research design is available in the Nature Research Reporting Summary linked to this article.

## Supplementary information

Supplementary Information

Reporting Summary

## Data Availability

This study includes no data deposited in external repositories. All data generated or analyzed during this study are included in the article and its Supplementary Information files. Generation and validation of RPE-specific *Akt2* cKO and *Akt2* KI mice, OCT, data for gain and loss of function of Akt1 in the RPE, clinical data of nondiabetic and diabetic mice, human data and basic characteristics of human RPE cadaver tissue donors are provided in the Supplementary Information files. Source data are provided with this paper.

## Source data

Source Data
