# Supplementary material for: Reducing Akt2 in retinal pigment epithelial cells causes a compensatory increase in Akt1 and attenuates diabetic retinopathy
Source: Nat Commun. 2022 Oct 13;13:6045. doi: 10.1038/s41467-022-33773-0 (PMC9561713; doi:10.1038/s41467-022-33773-0)
Supplement: Supplementary file 1 — Supplementary Information [file 41467_2022_33773_MOESM1_ESM.pdf]

Reducing Akt2 in retinal pigment epithelial cells causes a compensatory increase in Akt1 and attenuates diabetic retinopathy

Supplementary Information

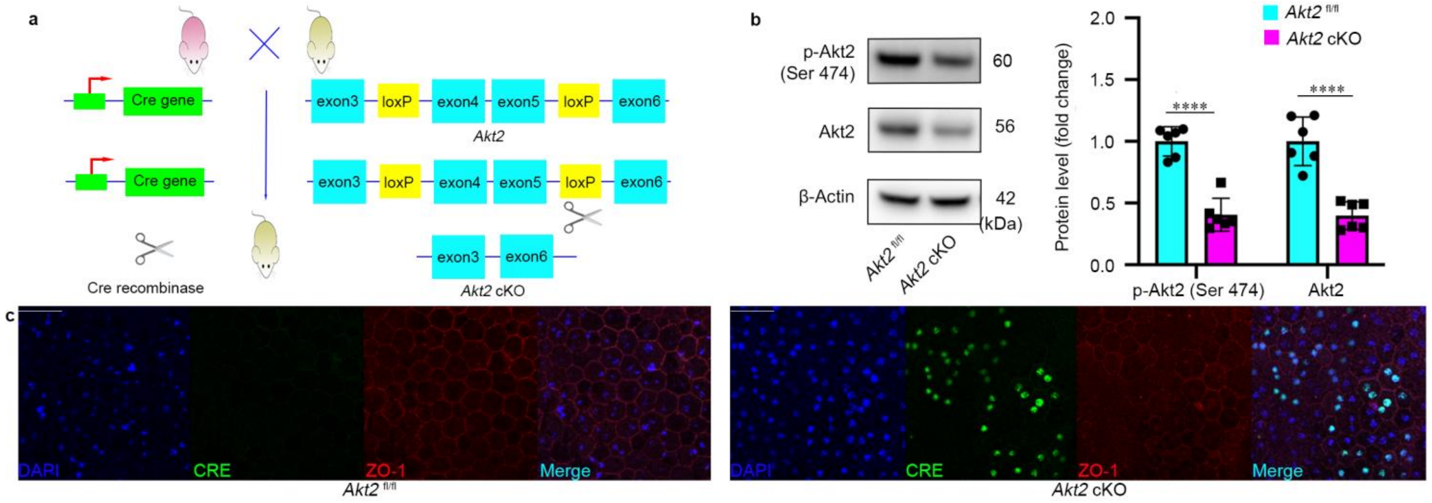

Supplementary Figure 1 - Generation and validation of RPE-specific *Akt2* cKO mice. (a) RPE-specific *Akt2* cKO mice were generated by mating *Akt2*$^{fl/fl}$ mice with *Best1*-Cre mice followed by cross-mating the progeny to generate the Cre-expressing mice homozygous for the floxed allele of *Akt2*. The *Akt2*$^{fl/fl}$ mice contain loxP sites flanking exons 4 and 5 of *Akt2*; the *Best1*-Cre mice express Cre recombinase under the control of the RPE-specific *Best1* promoter. Thus, mating these two strains results in progeny where exons 4 and 5 of *Akt2* are excised by Cre recombinase, making the resulting Akt2 protein dysfunctional, specifically in the RPE. (b) Representative immunoblot and densitometry graph show that the protein levels of phospho-Akt2 and Akt2 are decreased in *Akt2* cKO RPE compared to *Akt2*$^{fl/fl}$ RPE. (c) Representative RPE flat-mounts stained for Cre (green) show no Cre expression in *Akt2*$^{fl/fl}$ mice and mosaic expression of the Cre in *Akt2* cKO mice. ZO-1, red; DAPI, blue; merge, Cyan; Scale bar: 50 µm. In b, $n = 6$. Data are presented as mean ± SD. **** p <0.0001. Statistical test used in this study is two tailed, unpaired t-test. Exact p-values are: (b) p-Akt2: $p < 0.0001$, 95% CI -0.7563 to -0.4328, $R^2 = 0.8702$ (*Akt2* cKO vs. *Akt2* $^{fl/fl}$); Akt2: $p < 0.0001$, 95% CI -0.807 to -0.3971, $R^2 = 0.8108$ (*Akt2* cKO vs. *Akt2* $^{fl/fl}$). Source data are provided as a Source Data file.

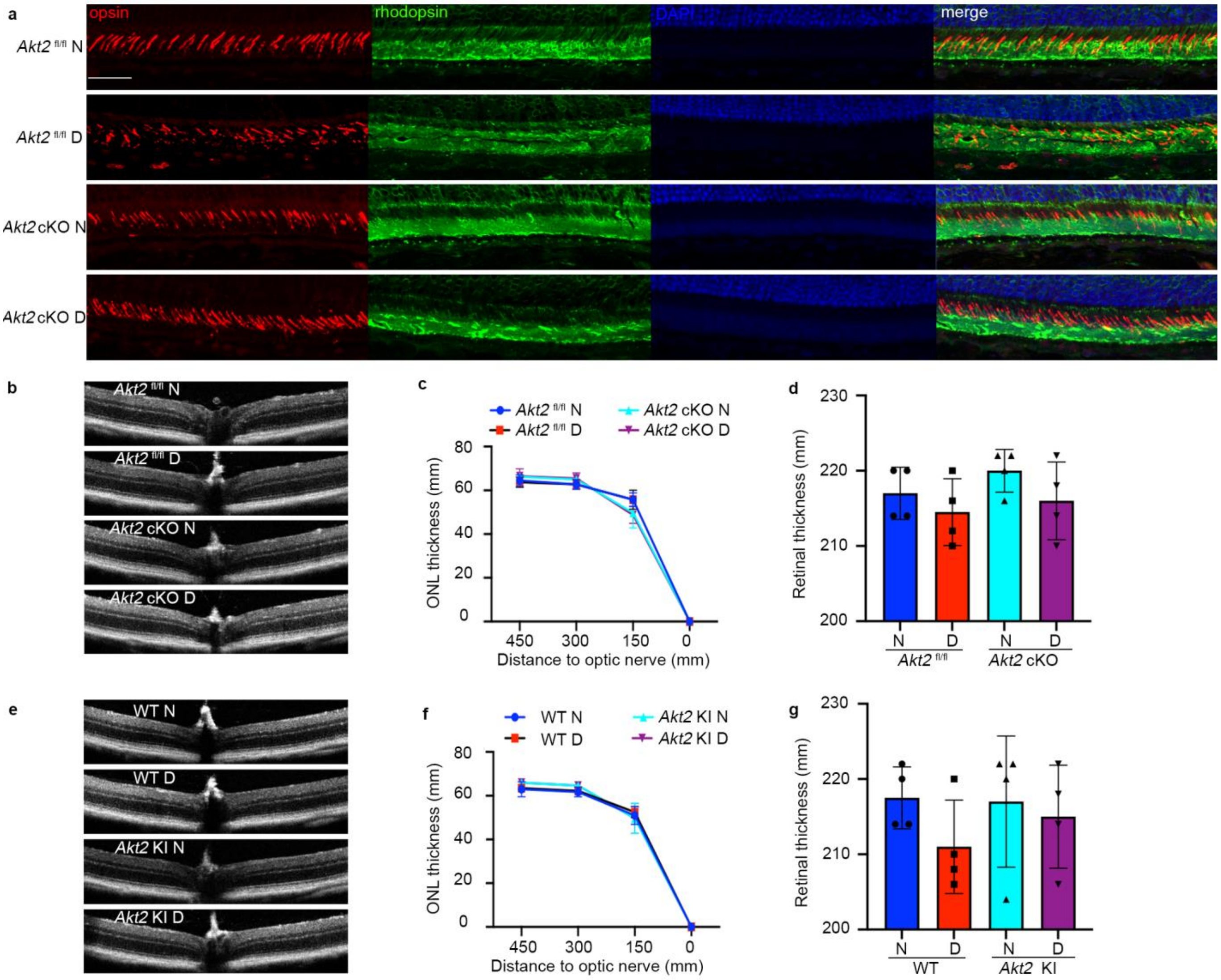

Supplementary Figure 2 – Retinal rod and cone morphology, OCT images and quantitation of the thickness of the whole retina and ONL. (a) Cones (stained with opsin antibody; red) are disorganized in *Akt2*$^{fl/fl}$ diabetic mice, while the *Akt2* cKO retina had normal parallel-arrangement of outer and inner segments; Rod photoreceptors (stained with rhodopsin; green) had similar morphology in *Akt2*$^{fl/fl}$ and *Akt2* cKO non-diabetic and diabetic mouse retinas (at 2 month duration of diabetes). DAPI, blue. Scale bar: 100 µm. (b) Representative OCT images and quantification of (c) ONL and (d) whole retina thickness in diabetic (8 months of diabetes) and age-matched non-diabetic *Akt2*$^{fl/fl}$ and *Akt2* cKO mice. Neither induction of diabetes nor *Akt2* cKO affected the thickness of the whole retina or ONL. *n* = 4 mice for each group. (e) Representative OCT images and quantification of (f) ONL and (g) whole retina thickness in diabetic (8 months of diabetes) and age-matched non-diabetic WT and *Akt2* KI mice. Neither induction of diabetes nor RPE-specific *Akt2* KI affected the thickness of the whole retina or ONL. *n* = 4 mice for each group. WT, wild type; KI, knock-in; cKO, conditional knock-out; N, non-diabetic; D, diabetic.

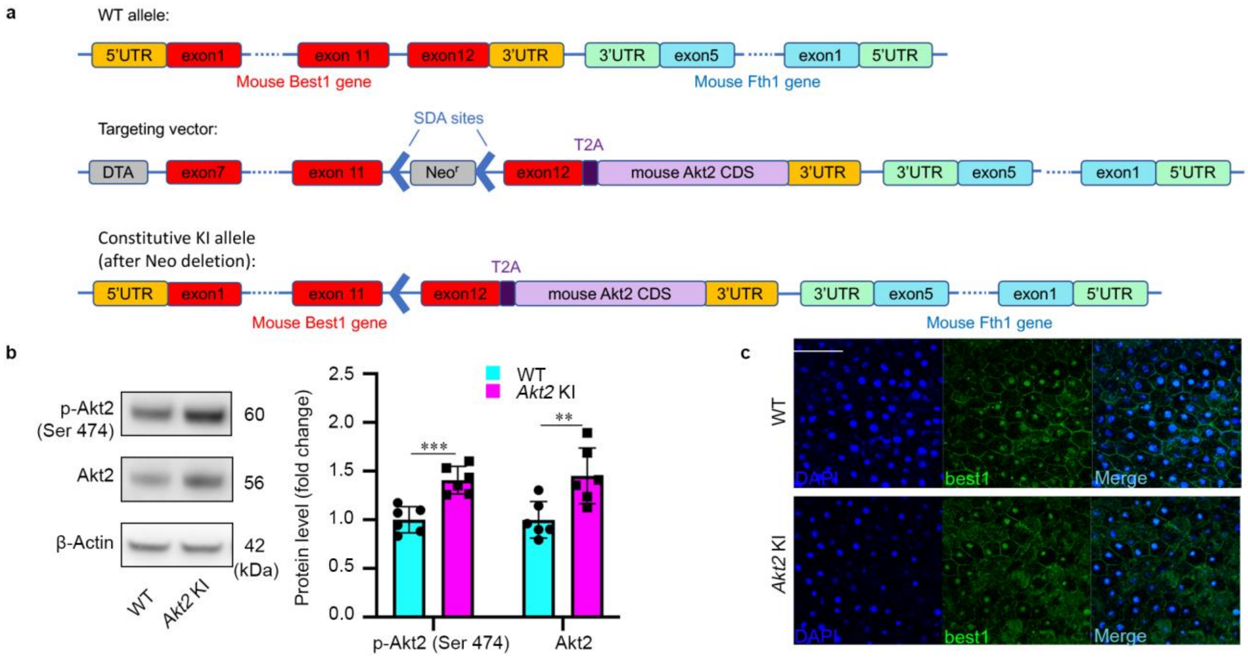

Supplementary Figure 3 - Generation and validation of RPE-specific *Akt2* KI mice. (a) RPE-specific *Akt2* KI mice were generated by Cyagen. Briefly, the T2A sequence followed by *Akt* 2 coding sequence (CDS) was inserted between the last exon and the 3' untranslated region (3'UTR) of the mouse *Best1* gene specifically expressed in the RPE. The Neo cassette flanked with self-deletion anchors (SDA) was inserted in the intron area between exons 11 and 12 of the mouse *Best1* for germ cell deletion of the gene. (b) Representative immunoblot and densitometry graph show that protein levels of phospho-Akt2 and Akt2 are higher in *Akt2* KI RPE compared to wild-type (WT) RPE. (c) A portion (60-70%) of RPE cells express Best1 (green) in the wild type and *Akt2* KI mice. In b, $n = 6$. Data are presented as mean ± SD. ** $p < 0.01$, and *** $p < 0.001$. Statistical test used in this study is two tailed, unpaired t-test. Exact p-values are: (b) p-Akt2: $p = 0.0005$, 95% CI 0.2295 to 0.5838, $R^2 = 0.7235$ (*Akt2* KI vs. WT); Akt2: $p = 0.0092$, 95% CI 0.1388 to 0.7634, $R^2 = 0.5088$ (*Akt2* KI vs. WT). Source data are provided as a Source Data file.

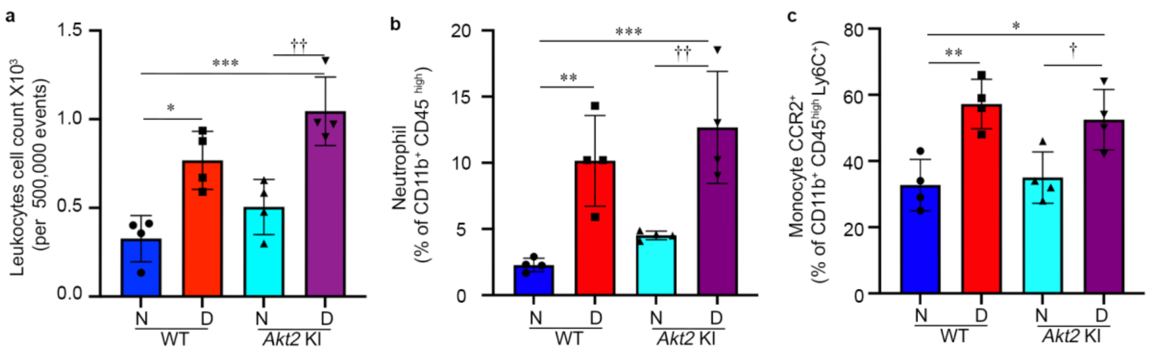

Supplementary Figure 4 – RPE-specific *Akt2* KI does not have a protective effect on diabetes-induced changes in the immune cell population in the retina (2 months of diabetes). (a) The absolute number of infiltrating leukocytes ($CD11b^+$ $CD45^{high}$ per 500,000 events) was increased in both WT and *Akt2* KI diabetic mice compared to non-diabetic animals; *Akt2* KI has no protective effects in inhibiting the diabetes-induced increase of infiltrating leukocytes. (b) The neutrophil population ($CD11b^+CD45^{high}Ly6C^{high}Ly6G^+$) and (c) monocyte population ($CD11b^+CD45^{high}Ly6C^{high}CCR2^+$) were increased in both diabetic WT and *Akt2* KI mice compared to appropriate non-diabetic controls. Data are shown as Mean ± SD. $n = 4$ animals per group, * $p < 0.05$, ** $p < 0.01$ and *** $p < 0.001$ represent changes versus non-diabetic WT control. † $p < 0.05$, and †† $p < 0.01$, denotes changes with respect to non-diabetic *Akt2* KI mice. Statistical tests used in this study are One-way ANOVA followed by a Tukey's post hoc test. Exact *p*-values are: (a) $p = 0.0107$ (WT D vs. WT N), $p = 0.0002$ (*Akt2* KI D vs. WT N), $p = 0.0025$ (*Akt2* KI D vs. *Akt2* KI N). (b) $p = 0.0074$ (WT D vs. WT N), $p = 0.0008$ (*Akt2* KI D vs. WT N), $p = 0.0057$ (*Akt2* KI D vs. *Akt2* KI N). (C) $p = 0.0049$ (WT D vs. WT N), $p = 0.0209$ (*Akt2* KI D vs. WT N), $p = 0.0418$ (*Akt2* KI D vs. *Akt2* KI N). WT, wild type; N, non-diabetic; D, diabetic; KI, knock-in.

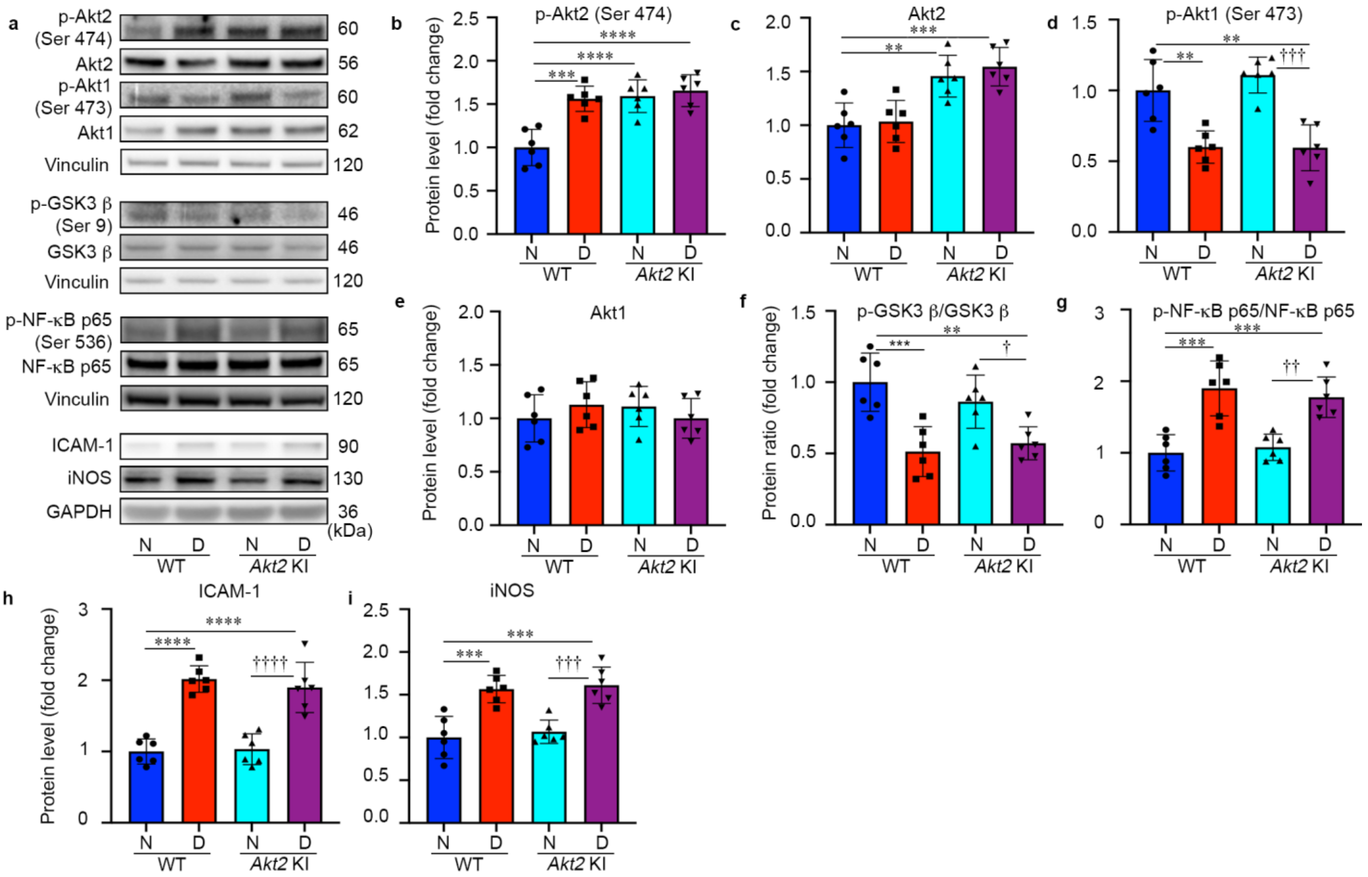

Supplemental Figure 5 - *Akt2* KI does not affect the Akt1/GSK3β/NF-κB signaling pathway (2 months of diabetes). (a) Representative immunoblots and quantification of (b) phospho- and (c) total Akt2, (d) phospho- and (e) total Akt1, (f) the ratio of phospho- and total GSK3β, (g) the ratio of phospho- and total NF-κB protein and inflammatory markers (h) ICAM-1, and (i) iNOS in RPE lysates. Vinculin and GAPDH served as internal controls. Induction of diabetes in WT mice resulted in increased p-Akt2 and decreased p-Akt1 without affecting the total protein levels of either Akt1 or Akt2. *Akt2* KI resulted in the increase of p-Akt2 in both diabetic and non-diabetic animals, compared to non-diabetic WT, but did not affect the levels of either p-Akt1 or total Akt1 compared to WT controls. In addition, *Akt2* KI did not alter the ratios of p-GSK3β/total GSK3β or p-NF-κB/total NF-κB, nor the levels of ICAM-1 or iNOS compared to the WT control groups. In (b-i), $n = 6$ mice for each group, data are shown as Mean ± SD. ** $p < 0.01$, *** $p < 0.001$ and **** $p < 0.0001$ represent changes versus non-diabetic WT control. † $p < 0.05$, †† $p < 0.01$, ††† $p < 0.001$ and †††† $p < 0.0001$ shows changes versus *Akt2* KI non-diabetic mice. Statistical tests used in this study are One-way ANOVA followed by a Tukey's post hoc test. Exact *p*-values are: (b) $p = 0.0002$ (WT D vs. WT N), $p < 0.0001$ for the rest. (c) $p = 0.0031$ (*Akt2* KI N vs. WT N), $p = 0.0005$ (*Akt2* KI D vs. WT N). (d) $p = 0.0018$ (WT D vs. WT N), $p = 0.0016$ (*Akt2* KI D vs. WT N), $p = 0.0001$ (*Akt2* KI D vs. *Akt2* KI N). (f) $p = 0.0029$ (WT D vs. WT N), $p = 0.0069$ (*Akt2* KI D vs. WT N), $p = 0.0399$ (*Akt2* KI D vs. *Akt2* KI N). (g) $p = 0.0005$ (WT D vs. WT N), $p = 0.002$ (*Akt2* KI D vs. WT N), $p = 0.0399$ (*Akt2* KI D vs. *Akt2*

KI N). (h) $p = 0.0001$ (WT D vs. WT N), $p = 0.0007$ (*Akt2* KI D vs. WT N), $p = 0.0021$ (*Akt2* KI D vs. *Akt2* KI N). (j) $p < 0.0001$ for all comparisons. (k) $p = 0.0003$ (WT D vs. WT N), $p = 0.0001$ (*Akt2* KI D vs. WT N), $p = 0.0005$ (*Akt2* KI D vs. *Akt2* KI N). Source data are provided as a Source Data file. WT, wild type; N, non-diabetic; D, diabetic; KI, knock-in.

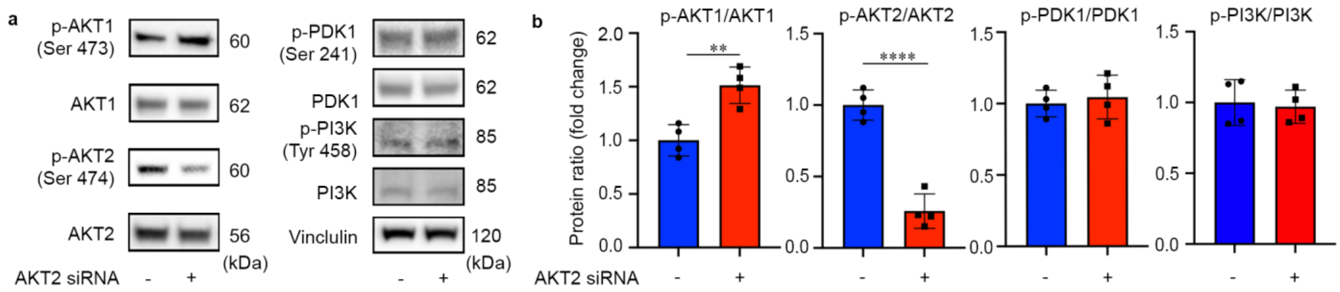

Supplementary Figure 6 - *AKT2* siRNA decreased the ratio of phospho-AKT2/total AKT2 and increased the ratio of phospho-AKT1/AKT1 in human fRPE cells cultured under high glucose conditions. (a) Representative immunoblots of phospho- and total forms of AKT1, AKT2, PDK1, and PI3K after human fRPE cells were cultured in high glucose medium with and without *AKT2* siRNA. (b) *AKT2* siRNA decreases the ratio of phospho-AKT2/AKT2 and increases the ratio of phospho-AKT1/AKT1 in high glucose conditions. There were no significant differences in the ratios of phospho-PDK1/PDK1 and phospho-PI3K/PI3K between *AKT2* siRNA treated and control groups. Values are the mean of 4 replicates ± SD. ** $p < 0.01$ and **** $p < 0.0001$ versus *AKT2* siRNA control treatment. Statistical test used in this study is two tailed, unpaired *t*-test. Exact *p*-values are: (b) p-AKT1/AKT1: $p = 0.0038$, 95% CI 0.2389 to 0.7861, $R^2 = 0.7778$ (*AKT2* siRNA vs. control); p-AKT2/AKT2: $p < 0.0001$, 95% CI -0.9389 to -0.5461, $R^2 = 0.9345$ (siRNA vs. control). Source data are provided as a Source Data file.

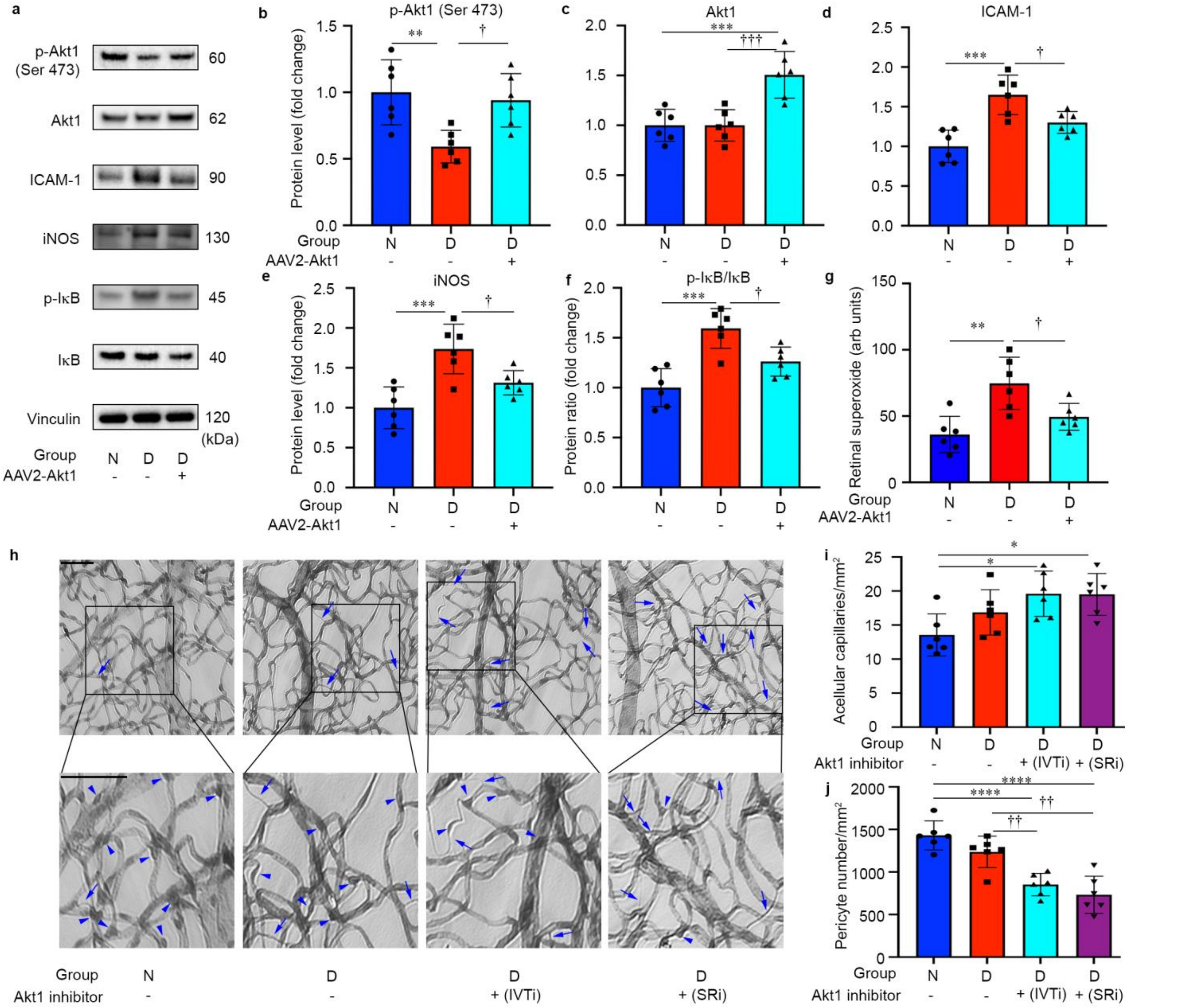

Supplementary Figure 7 - Gain and loss of function of Akt1 in the RPE affect the pathogenesis of DR. (a) Representative immunoblots of phospho-Akt1, Akt1 and inflammatory markers in the RPE of diabetic mice overexpressing Akt1 after subretinal injection of AAV2-hRPE-GFP-P2A-mAkt1. Subretinal injection of AAV2-hRPE-GFP-P2A-mAkt1 increased (b) phospho- and (c) total Akt1 in the RPE of diabetic mice compared to control diabetic mice treated with AAV2-hRPE-EGFP. Diabetes-induced increases of (d) ICAM-1, (e) iNOS, and (f) the ratio of pIκB/IκB were significantly lowered in AAV2-hRPE-GFP-P2A-mAkt1 treated diabetic mice. (g) Increased retinal production of superoxide in diabetic mice (control) was also attenuated in AAV2-hRPE-GFP-P2A-mAkt1 treated diabetic mice, (h) Representative micrographs of retinal vessels from diabetic mice (4 months of diabetes) with and without Akt1 inhibitor treatment (IVTi and SRi) and age-matched non-diabetic mice. Scale bar: 100 µm. Arrows indicate degenerated capillaries and arrowheads indicate capillary pericytes. A 4 month duration of diabetes did not significantly increase the number of (i)

degenerated capillaries or (g) decrease the number of retinal capillary pericytes in diabetic mice compared to non-diabetic animals. Administration of an Akt1 inhibitor either by IVTi or SRi in diabetic mice significantly increased (i) acellular capillaries and (g) decreased pericytes in the retina at 4 months of diabetes compared to age matched non-diabetic mice. Scale bar: 100 µm. In (b-g, i-j), n = 6 mice for each group, the data are expressed as mean ± SD. * $p < 0.05$, ** $p < 0.01$, *** $p < 0.001$, and **** $p < 0.0001$ versus non-diabetic control (N). † $p < 0.05$, †† $p < 0.01$, and ††† $p < 0.001$ versus diabetic mice (D) without treatment. Statistical tests used in this study are One-way ANOVA followed by a Tukey's post hoc test. Exact *p*-values are: (b) $p = 0.0068$ (WT D vs. WT N), $p = 0.0196$ (WT D + AAV2-Akt1 vs. WT N). (c) $p = 0.0008$ (WT D + AAV2-Akt1 vs. WT N) and (WT D + AAV2-Akt1 vs. WT D). (d) $p = 0.0002$ (WT D vs. WT N), $p = 0.0238$ (WT D + AAV2-Akt1 vs. WT D). (e) $p = 0.0004$ (WT D vs. WT N), $p = 0.0264$ (WT D + AAV2-Akt1 vs. WT D). (f) $p < 0.0001$ (WT D vs. WT N), $p = 0.0141$ (WT D + AAV2-Akt1 vs. WT D). (i) $p = 0.0185$ (WT D +Akt1 inhibitor/IVTi vs. WT N), $p = 0.0208$ (WT D + Akt1 inhibitor/SRi vs. WT N). (j) $p = 0.007$ (WT D +Akt1 inhibitor/IVTi vs. WT D), $p = 0.0005$ (WT D + Akt1 inhibitor/SRi vs. WT D), p < 0.0001 for the rest. Source data are provided as a Source Data file. IVTi, intravitreal injection; SRi, subretinal injection.

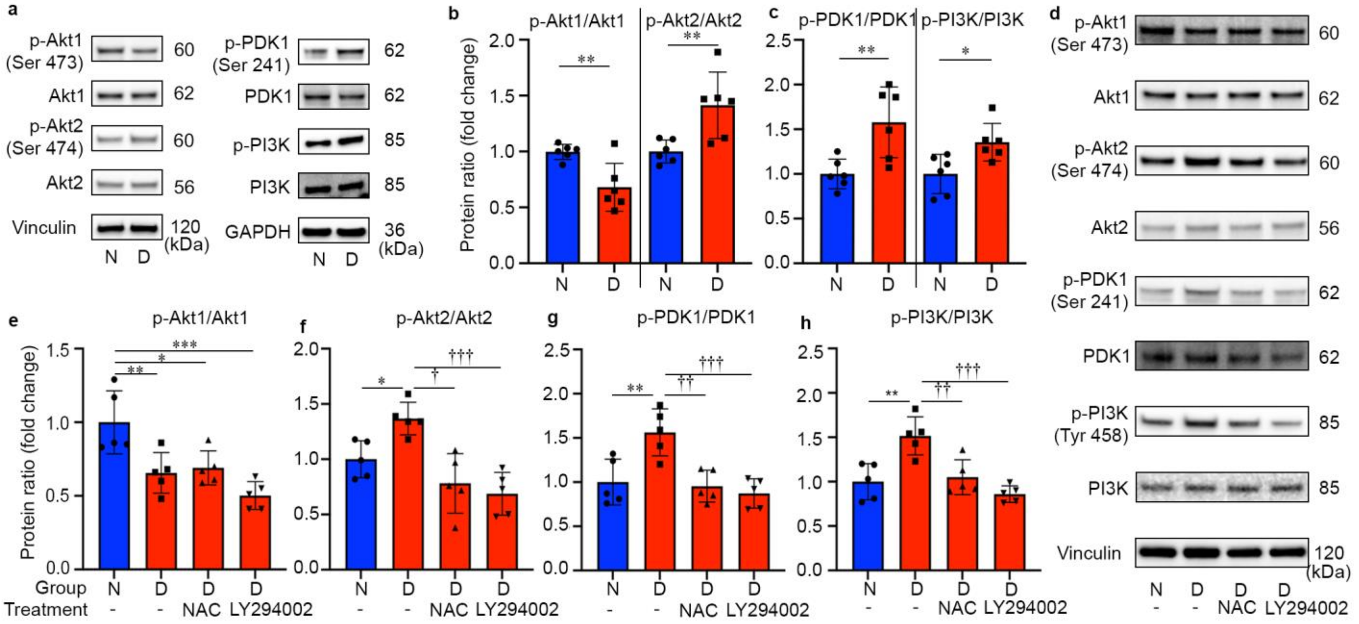

Supplementary Figure 8 - Diabetes increase Akt2 activity in RPE possibly through ROS mediated PI3K/PDK1 signaling; decreased Akt1 activity is not mediated by ROS and is independent of PI3K/PDK1 signaling. Representative (a) immunoblots and (b) densitometry graphs demonstrating that diabetes decreased the phospho-Akt1/Akt1 ratio and increased the phospho-Akt2/Akt2 ratio, (c) diabetes increased phospho-PDK1/ PDK1 and phospho-PI3K/PI3K ratios in RPE compared to wild type non-diabetic mice. (d) Representative immunoblots for phospho- and total form of Akt1, Akt2, PDK1 and PI3K from RPE explants isolated from diabetic (D) mice (treated with and without antioxidant (NAC) or PI3K inhibitor (LY294002) in 25mM glucose medium) and non-diabetic (N) mice (cultured in 5mM glucose medium). (e) Diabetes reduced the ratio of phospho-Akt1/Akt1 in RPE compared to non-diabetic groups; Neither NAC nor LY294002 had effect on phospho- or total Akt1 levels in diabetic RPE explants, compared to RPE explants isolated from diabetic mice without any treatment. NAC and LY294002 treatment both show lower ratios of (f) p-Akt2/Akt2, (g) p-PDK1/ PDK1, and (h) p-PI3K/PI3K in comparison to the RPE explants isolated from diabetic mice without any treatment. In b-c (n = 6), e-h (n = 5), data are shown as Mean ± SD. * $p < 0.05$, ** $p < 0.01$ and *** $p < 0.001$ versus the non-diabetic mice. † $p < 0.05$, †† $p < 0.01$ and ††† $p < 0.001$ versus RPE choroid isolated from diabetic mice without treatment. Statistical test used in b and c is two tailed, unpaired t-test, in e-h is One-way ANOVA followed by a Tukey's post hoc test. Exact p-values are: (b) p-Akt1/Akt1: $p = 0.0063$, 95% CI -0.5214 to -0.1119, $R^2 = 0.5429$ (WT D vs. WT N); p-Akt2/Akt2: $p = 0.0091$, 95% CI 0.128 to 0.6987, $R^2 = 0.5103$ (WT D vs. WT N). (c) p-PDK1/PDK1: $p = 0.0079$, 95% CI 0.1886 to 0.968, $R^2 = 0.5223$ (WT D vs. WT N); p-PI3K/PI3K: $p = 0.0168$, 95% CI 0.07893 to 0.6311, $R^2 = 0.4508$ (WT D vs. WT N). (e) $p = 0.0098$ (WT D vs. WT N), $p = 0.0204$ (WT D + NAC vs. WT N), $p = 0.0004$ (WT D +

LY294002 vs. WT N). (f) $p = 0.0453$ (WT D vs. WT N), $p = 0.0014$ (WT D + NAC vs. WT D), $p = 0.0003$ (WT D + LY294002 vs. WT D). (g) $p = 0.0053$ (WT D vs. WT N), $p = 0.0027$ (WT D + NAC vs. WT D), $p = 0.0008$ (WT D + LY294002 vs. WT D). (h) $p = 0.0021$ (WT D vs. WT N), $p = 0.005$ (WT D + NAC vs. WT D), $p = 0.0002$ (WT D + LY294002 vs. WT D). Source data are provided as a Source Data file.

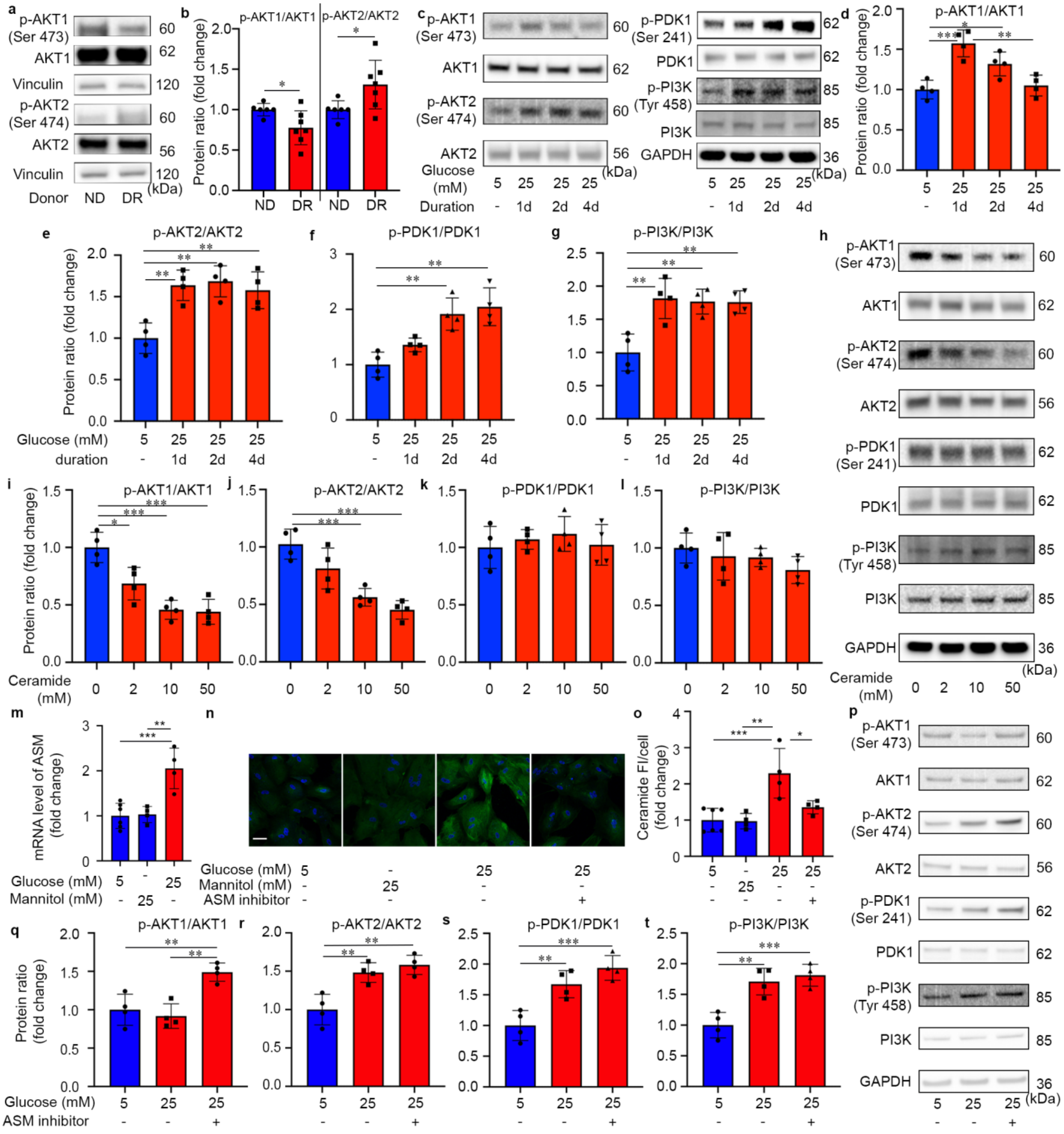

Supplementary Figure 9 - High glucose increased RPE Akt2 activity through PI3K/PDK1; ceramide lowered RPE Akt1 activity independent of the PI3K/PDK1 signaling pathway. Representative immunoblots from (a) human RPE tissue show that (b) the ratio of p-AKT1/AKT1 was decreased and the ratio of p-AKT2/AKT2 was increased in DR patients compared to non-diabetic patients. *n* = 6 for Non-diabetic group, *n* = 7 for diabetic retinopathy group (c) Representative immunoblots for phospho- and total AKT1, AKT2, PDK1 and PI3K from human fetal RPE cells cultured in low glucose (5 mM) and high glucose (25 mM). (d) High glucose increased the ratio of p-AKT1/AKT1 in fRPE cells

after 1 day and 2 days of culture compared to control fRPE cells cultured in a low glucose environment. The ratio of p-AKT1/AKT1 in high glucose decreased after 2 days, and was near control levels after 4 days. (e) High glucose treatment for 2 and 4 days increased the ratio of p-AKT2/AKT2, alongside elevated (f) phospho-PDK1/ PDK1, and (g) phospho-PI3K/PI3K. (h) Representative immunoblots for phospho- and total AKT1, AKT2, PDK1 and PI3K from human fRPE cells cultured in high glucose with or without ceramide (dissolved in DMSO) for 4 days. Ceramide decreased the (i) p-AKT1/AKT1 ratio at the dose of 2 μM and decreased (j) the p-AKT2/AKT2 ratio starting at the dose of 10 μM, compared to control fRPE cells cultured in presence of 0.05% DMSO (vehicle control). Ceramide treatment did not alter (k) the ratios of p-PDK1/ PDK1, or (l) p-PI3K/PI3K. (m) High glucose treatment for 4 days increased the mRNA level of ASM in human fRPE cells compared to the low glucose and osmotic control (25mM mannitol). (n) Representative images of low glucose, osmotic control, high glucose, and high glucose with ASM inhibitor treatment for 4 days showing ceramide (green) and nuclear staining (blue). Scale bar = 50 μm. (o) Quantitation of ceramide-staining fluorescence intensity from panel (n) show more ceramide is accumulated in high glucose treated human fRPE cells compared to low glucose and osmotic controls. (p) Representative immunoblots from human RPE cells show that (q) the ratio of p-AKT1/AKT1 was close to normal (as seen in d) after 4 days incubation in high glucose medium. and that the ASM inhibitor significantly increases AKT1 activity (similar to cells treated with high glucose for 1 day in d). The ratios of p-AKT2/AKT2 (r), p-PDK1/ PDK1(s), and p-PI3K/PI3K(t) were significantly increased in high glucose-treated RPE cells without any significant difference between ASM inhibitor treated or non-treated groups. In c-l, q-t, n = 4 replicates; m-o, n = 6 replicates for 5mM glucose group, 4 replicates for the rest. Data are shown as Mean ± SD. * $p < 0.05$, ** $p < 0.01$ and *** $p < 0.001$. Statistical test used in b is two tailed, unpaired t-test, in d-g, i-m, o-t is One-way ANOVA followed by a Tukey's post hoc test. Exact p-values are: (b) p-AKT1/AKT1: $p = 0.0315$, 95% CI -0.4247 to -0.0239, $R^2 = 0.3556$ (DR vs. ND); p-Akt2/Akt2: $p = 0.0365$, 95% CI 0.0233 to 0.5967, $R^2 = 0.3399$ (WT D vs. WT N). (d) $p = 0.0005$ (25mM/1day vs. 5mM), $p = 0.0353$ (25mM/2day vs. 5mM), $p = 0.0011$ (25mM/4day vs. 5mM). (e) $p = 0.0029$ (25mM/1day vs. 5mM), $p = 0.0016$ (25mM/2day vs. 5mM), $p = 0.006$ (25mM/4day vs. 5mM). (f) $p = 0.0014$ (25mM/2day vs. 5mM), $p = 0.0004$ (25mM/4day vs. 5mM). (g) $p = 0.0022$ (25mM/1day vs. 5mM), $p = 0.0036$ (25mM/2day vs. 5mM), $p = 0.0038$ (25mM/4day vs. 5mM). (i) $p = 0.0126$ (Ceramide/2μM vs. control), $p = 0.0002$ (Ceramide/10μM vs. control), $p = 0.0001$ (Ceramide/50μM vs. control). (j) $p = 0.0009$ (Ceramide/10μM vs. control), $p = 0.0001$ (Ceramide/50μM vs. control). (m) $p = 0.0008$ (25mM glucose vs. 5mM glucose), $p = 0.0021$ (25mM glucose vs. 25mM Mannitol). (o) $p = 0.0008$ (25mM glucose vs. 5mM glucose), $p = 0.0016$ (25mM glucose vs. 25mM Mannitol), $p = 0.0211$ (25mM glucose + ASM inhibitor

vs. 25mM glucose). (q) p = 0.0057 (25mM glucose + ASM inhibitor vs. 5mM glucose), p = 0.0021 (25mM glucose + ASM inhibitor vs. 25mM glucose). (r) p = 0.0044 (25mM glucose vs. 5mM glucose), p = 0.0013 (25mM glucose + ASM inhibitor vs. 5mM glucose). (s) p = 0.0054 (25mM glucose vs. 5mM glucose), p = 0.0006 (25mM glucose + ASM inhibitor vs. 5mM glucose). (t) p = 0.0019 (Ceramide/2μM vs. control), p = 0.0008 (Ceramide/10μM vs. control). Source data are provided as a Source Data file. Basic characteristics of control and diabetic human tissue are reported in Supplementary Table 2.

Supplementary Table 1 - Clinical data of non-diabetic (N) and diabetic mice (D) in short-term and long-term studies

	Group	Non-fasting blood glucose (mg/dL)	Body weight (g)	HbA_{1c}	
				%	mmol/mol
Short-term studies (2-3 month duration of diabetes)	WT-N	144 ± 14	32 ± 2	3.1 ± 0.1	11 ± 1.1
	WT-D	497 ± 45*	25 ± 2*	8.3 ± 0.2*	67 ± 2.0*
	Akt2 KI-N	139 ± 11	33 ± 3	3.1 ± 0.1	10 ± 0.8
	Akt2 KI-D	505 ± 50*	27 ± 2*	8.1 ± 0.4*	65 ± 3.7*
	$Akt2^{fl/fl}$-N	141 ± 13	33 ± 2	3.1 ± 0.1	10 ± 1.0
	$Akt2^{fl/fl}$-D	505 ± 43*	25 ± 3*	8.2 ± 0.3*	66 ± 2.7*
	Akt2 cKO-N	133 ± 11	33 ± 3	3.1 ± 0.1	10 ± 0.8
	Akt2 cKO-D	490 ± 53*	26 ± 3*	7.9 ± 0.4*	62 ± 4.0*
Long-term studies (8 month duration of diabetes)	WT-N	142 ± 14	42 ± 5	3.1 ± 0.1	10 ± 0.8
	WT-D	505 ± 30*	30 ± 1*	8.3 ± 0.3*	67 ± 2.9*
	Akt2 KI-N	139 ± 16	48 ± 5	3.0 ± 0.1	9.4 ± 0.5
	Akt2 KI-D	507 ± 41*	30 ± 1*	8.0 ± 0.1*	64 ± 1.4*
	$Akt2^{fl/fl}$ -N	157 ± 13	41 ± 4	3.0 ± 0.1	9.4 ± 0.5
	$Akt2^{fl/fl}$ -D	489 ± 22*	31 ± 2*	8.0 ± 0.2*	6 4 ± 2.6*
	Akt2 cKO-N	140 ± 17	43 ± 5	3.1 ± 0.1	9.5 ± 0.6
	Akt2 cKO-D	505 ± 28*	30 ± 2*	8.1 ± 0.3*	65 ± 3.2*
AAV2-hRPE-GFP-mAkt1 treatment	WT-N	135 ± 17	31 ± 2	3.1 ± 0.1	9.7 ± 0.8
	WT-D	483 ± 97*	23 ± 2*	8.0 ± 0.4*	64 ± 4.7*
	WT-D+AAV2	473 ± 73*	23 ± 3*	8.1 ± 0.2*	65 ± 1.5*
Akt1 inhibitor treatment	WT-N	138 ± 13	35 ± 4	3.1 ± 0.1	10 ± 0.6
	WT-D	486 ± 52*	24 ± 2*	8.1 ± 0.2*	65 ± 2.0*
	WT-D + IVTi	435 ± 145*	23 ± 2*	8.1 ± 0.1*	65 ± 1.0*
	WT-D + SRi	446 ± 84*	23 ± 3*	8.2 ± 0.1*	65 ± 1.4*

Data are mean ± SD. In short term studies, n = 31for WT-N, n = 32 for WT-D, *Akt2* KI-N and *Akt2* KI-D, n = 39 for $Akt2^{fl/fl}$-N, n = 40 for $Akt2^{fl/fl}$-D and *Akt2* cKO-D, n = 42 *Akt2* cKO-N. n = 10 mice in each group for long term studies, n = 6 mice per group for AAV2-hRPE-GFP-mAkt1 treatment study. n = 6 mice per group for Akt1 inhibitor study. $^*p < 0.0001$ compared with appropriate N from each group. Statistical test used in this study is Two-tailed, Mann-Whitney *U*-test. N, Non-diabetic; D, Diabetic; WT, Wildtype; KI, knock-in; cKO, conditional knock-out; IVTi, intravitreal injection; SRi, subretinal injection.

Supplementary Table 2 – Basic characteristics of human RPE cadaver tissue donors

Group	Age (years) Average	Male (Number)	Female (Number)	Duration of Diabetes (Years)	HbA1c (%)	Race
ND	70 ± 8	4	2	-	-	Caucasian
DR	73 ± 10	5	2	11 ± 6.9	7.1 + 1.5	Caucasian

Data are mean ± SD. *n* = 6 for non-diabetic group, *n* = 7 for diabetic retinopathy group, Duration of Diabetes and HbA1c data is not accessible for one female case in DR group. ND, Non-diabetic; DR, Diabetic retinopathy.
