# Supplementary material for: Reducing Akt2 in retinal pigment epithelial cells causes a compensatory increase in Akt1 and attenuates diabetic retinopathy
Source: Nat Commun. 2022 Oct 13;13:6045. doi: 10.1038/s41467-022-33773-0 (PMC9561713; doi:10.1038/s41467-022-33773-0)
Supplement: Supplementary file 2 — Reporting Summary [file 41467_2022_33773_MOESM2_ESM.pdf]

nature research

Corresponding author(s): Debasish Sinha

Last updated by author(s): Aug 9, 2022

Reporting Summary

Nature Research wishes to improve the reproducibility of the work that we publish. This form provides structure for consistency and transparency in reporting. For further information on Nature Research policies, see our Editorial Policies and the Editorial Policy Checklist.

Statistics

For all statistical analyses, confirm that the following items are present in the figure legend, table legend, main text, or Methods section.

n/a	Confirmed	
☐	☒	The exact sample size (*n*) for each experimental group/condition, given as a discrete number and unit of measurement
☐	☒	A statement on whether measurements were taken from distinct samples or whether the same sample was measured repeatedly
☐	☒	The statistical test(s) used AND whether they are one- or two-sided<br>*Only common tests should be described solely by name; describe more complex techniques in the Methods section.*
☒	☐	A description of all covariates tested
☐	☒	A description of any assumptions or corrections, such as tests of normality and adjustment for multiple comparisons
☐	☒	A full description of the statistical parameters including central tendency (e.g. means) or other basic estimates (e.g. regression coefficient) AND variation (e.g. standard deviation) or associated estimates of uncertainty (e.g. confidence intervals)
☐	☒	For null hypothesis testing, the test statistic (e.g. *F*, *t*, *r*) with confidence intervals, effect sizes, degrees of freedom and *P* value noted<br>*Give P values as exact values whenever suitable.*
☒	☐	For Bayesian analysis, information on the choice of priors and Markov chain Monte Carlo settings
☒	☐	For hierarchical and complex designs, identification of the appropriate level for tests and full reporting of outcomes
☒	☐	Estimates of effect sizes (e.g. Cohen's *d*, Pearson's *r*), indicating how they were calculated

Our web collection on statistics for biologists contains articles on many of the points above.

Software and code

Policy information about availability of computer code

Data collection	Zen software (Zeiss) version 2.3, Azure Biosystems c200 imaging system (Azure Biosystems). Flow Cytometry (LSRII)
Data analysis	Excel (Microsoft), GraphPad Prism v9, NIS-Elements AR 5.21.01 (Nikon). Flowjo v9. ImageJ (version 1.51).

For manuscripts utilizing custom algorithms or software that are central to the research but not yet described in published literature, software must be made available to editors and reviewers. We strongly encourage code deposition in a community repository (e.g. GitHub). See the Nature Research guidelines for submitting code & software for further information.

Data

Policy information about availability of data

All manuscripts must include a data availability statement. This statement should provide the following information, where applicable:

- Accession codes, unique identifiers, or web links for publicly available datasets
- A list of figures that have associated raw data
- A description of any restrictions on data availability

This study includes no data deposited in external repositories. All data generated or analyzed during this study are included in the article and its Supplementary Information files. Generation and validation of RPE-specific Akt2 cKO and Akt2 KI mice; OCT; Data for gain and loss of function of Akt1 in the RPE; Clinical data of non-diabetic and diabetic mice; Human data and basic characteristics of human RPE cadaver tissue donors are provided in the Supplementary Information files. The ratio of phospho-Akt1/Akt1, phospho-Akt2/Akt2 for all figures, alternative retinal vascular permeability data, validation of potential cross contamination between RPE and retinal tissues, original uncropped western blot images are provided in the Source Data file. Raw data for Figure 1-9 and Supplementary Figures 1-9 are provided in the Raw Data file.

Field-specific reporting

Please select the one below that is the best fit for your research. If you are not sure, read the appropriate sections before making your selection.

[x] Life sciences [ ] Behavioural & social sciences [ ] Ecological, evolutionary & environmental sciences

For a reference copy of the document with all sections, see nature.com/documents/nr-reporting-summary-flat.pdf

Life sciences study design

All studies must disclose on these points even when the disclosure is negative.

Sample size	Sample sizes were determined based on power calculation, showing a difference of 25% and up to 20% SD, alpha=0.05 and beta=0.2
Data exclusions	No data were excluded, but some samples (<1%) were excluded from the experiments due to technical challenges during tissue collection
Replication	At least 3 independent experiments were performed to reliably reproduce the results of the experiments.
Randomization	Animals (from the respective genotypes) were randomly allocated to each group
Blinding	To eliminate bias, individuals handing core-facility instruments or performing analysis on specific experiments were blinded to mouse genotype identity as well as the identity of the experimental groups.

Reporting for specific materials, systems and methods

We require information from authors about some types of materials, experimental systems and methods used in many studies. Here, indicate whether each material, system or method listed is relevant to your study. If you are not sure if a list item applies to your research, read the appropriate section before selecting a response.

Materials & experimental systems

n/a	Involved in the study	
[ ]	[x]	Antibodies
[ ]	[x]	Eukaryotic cell lines
[x]	[ ]	Palaeontology and archaeology
[ ]	[x]	Animals and other organisms
[x]	[ ]	Human research participants
[x]	[ ]	Clinical data
[x]	[ ]	Dual use research of concern

Methods

n/a	Involved in the study	
[x]	[ ]	ChIP-seq
[ ]	[x]	Flow cytometry
[x]	[ ]	MRI-based neuroimaging

Antibodies

Antibodies used	Unless otherwise stated, primary antibody was used at a dilution of 1:1000. iNOS (Cell Signaling Technology,2982S), ICAM-1 (Proteintech,10020-1-AP), Phospho-IκBα (Ser32)(Cell Signaling Technology,2859T, clone: 14D4), IκBα (Cell Signaling Technology,4812S, clone: 44D4), Phospho-Akt2 (Ser474) (Cell Signaling Technology,8599S, clone:D3H2), Akt2(Cell Signaling Technology, 3063S, clone: D3H2), Phospho-Akt1 (Ser473), (Cell Signaling Technology, 9018S, clone:D7F10), Akt1 (Cell Signaling Technology, 2938S, clone: D7F10), Phospho-NF-κB p65 (Ser536) (Thermo Fisher Scientific, MA515160, clone: T.849.2), NF-κB p65 (Thermo Fisher Scientific, 10745-1-AP), Phospho-GSK-3β (Ser9) (Cell Signaling Technology, 5558S, clone: D85E12), GSK-3β (85-86173-11, Thermo Fisher Scientific), GAPDH (5174S, Cell Signaling Technology), and Vinculin (ab129002, Abcam). RPE65 (Invitrogen, MA-532633),rhodopsin (Abcam, ab98887), Alexa Fluor 594 conjugated ZO-1 (Invitrogen, 339194), Donkey anti-Rabbit-Alexa Fluor 488 (Invitrogen, A21206), Donkey anti-mouse-Alexa Fluor 488 (Invitrogen, A21202), Goat anti-Rabbit-Alexa Fluor 568, (Invitrogen, A11011), opsin (MilliporeSigma, AB5405), Cre antibody (MilliporeSigma, MAB3120), Best1 antibody (Bioss Antibodies, bs11040R), PDK1 (Cell Signaling Technology, 3062), phospho-PDK1 (1:500 dilution, Cell Signaling Technology,3438, clone: C49H12), PI3K (Cell Signaling Technology, 4257, Clone: 19H8), phospho-PI3K (1:500 dilution, Cell Signaling Technology, 4228), GAPDH (Cell Signaling Technology, 5174S, Clone: D16H11), Vinculin (Abcam, ab129002, Clone: EPR8185). RPE65 (Invitrogen, MA-532633, Clone: JM61-51) and rhodopsin (Abcam, ab98887, Clone: Rho 4D2), Monoclonal Anti-Ceramide antibody (Sigma-Aldrich, C8104, clone: 15B4).BV650 Rat Anti-Mouse CD11b (BD Biosciences, 563402, clone: M1/70); PE-Cy7 Rat anti-Mouse CD45 (BD Biosciences, 561868); FITC Rat anti-Mouse CCR2 (BioLegend, 561868, SC203G11); APC Rat Anti-Mouse Ly-6G (BD Biosciences, 560599, clone: 1A8), BV421 Rat Anti-Mouse Ly-6C (BD Biosciences, 562727, clone: Al-21).
Validation	The validation information for each antibody is:<br>iNOS polyclonal (2982S) from Cell Signaling Technology where they validate their antibodies from Western blot analysis of extracts from Raw264.7 cells, untreated or LPS-treated (1 µg/ml for 6 h), using iNOS Antibody (Mouse Specific): https://www.cellsignal.com/products/primary-antibodies/inos-antibody-mouse-specific/2982);<br>ICAM-1 polyclonal (10020-1-AP) from Proteintech, where they validate their antibodies from the mouse brain tissue at dilution of 1:2000 incubated at room temperature for 1.5 hours: https://www.ptglab.com/products/Icam1-Antibody-10020-1-AP.htm#product-

information);
Phospho-IκBα monoclonal (Ser32) (clone: 14D4, 2859T) from Cell Signaling Technology, where they validate their antibodies by Western blot analysis of extracts from HeLa and NIH/3T3 cells: https://www.cellsignal.com/products/primary-antibodies/phospho-ikba-ser32-14d4-rabbit-mab/2859);
IκBα monoclonal from Cell Signaling Technology where they validated their antibodies by Western blot analysis of extracts from control HeLa cells or HeLa cells. https://www.cellsignal.com/products/primary-antibodies/ikba-44d4-rabbit-mab/4812);
Phospho-Akt2 (Ser474) monoclonal (D3H) Rabbit mAb from Cell Signaling Technology, where they validated their antibodies by Western blot analysis of extracts from Akt1 (-/-) mouse embryonic fibroblasts (MEF) or Akt2 (-/-) MEF, untreated (-) or treated with Human Platelet-Derived Growth Factor AA (hPDGF-AA), https://www.cellsignal.com/products/primary-antibodies/phospho-akt2-ser474-d3h2-rabbit-mab-akt2-specific/8599?site-search-type=Products&N=4294956287&Ntt=phospho-akt2+%28ser474%29+%288599s&fromPage=plp&_requestid=2589303);
Akt2 monoclonal (D6G4) from Cell Signaling Technology, where they validated their antibodies by Western blot, https://www.cellsignal.com/products/primary-antibodies/akt2-d6g4-rabbit-mab/3063?site-search type=Products&N=4294956287&Ntt=akt2+%283063s&fromPage=plp&_requestid=2589373);
Phospho-Akt1 (Ser473) monoclonal (D7F10) from Cell Signaling Technology 9018S, where they validated their antibodies by Western blot analysis of extracts from LNCaP cells, https://www.cellsignal.com/products/primary-antibodies/phospho-akt1-ser473-d7f10-xp-rabbit-mab-akt1-specific/9018);
Akt1 monoclonal (C73H10) from Cell Signaling Technology,2938S, where they validated their antibodiesby Western blot analysis of extracts from various cell types https://www.cellsignal.com/products/primary-antibodies/akt1-c73h10-rabbit-mab/2938?site-search-type=Products&N=4294956287&Ntt=2938s%2C&fromPage=plp&_requestid=2589826);
Phospho-NF-κB p65 (Ser536) monoclonal(T.849.2) from Thermo Fisher Scientific, MA515160, host: rabbit, Reactivity: Dog, Hamster, Human, Mouse, Non-human primate, Pig, Ra, where they validated their antibodies validated in HeLa cells, https://www.thermofisher.com/antibody/product/Phospho-NFkB-p65-Ser536-Antibody-clone-T-849-2-Monoclonal/MA5-15160);
NF-κB p65 poluclonal from Thermo Fisher Scientific, 10745-1-AP, where they validated their antibodies using HepG2 cells: https://www.ptglab.com/products/p65-Antibody-10745-1-AP.htm#product-information);
Phospho-GSK-3β monoclonal (D85E12) (Ser9) from Cell Signaling Technology,5558S, (host: rabbit, Reactivity: Human, Mouse, Rat) where they validated their antibodies by Western blot analysis of extracts from PC-3 cells: https://www.cellsignal.com/products/primary-antibodies/phospho-gsk-3b-ser9-d85e12-xp-rabbit-mab/5558),
GAPDH (D16H11) monoclonal from Cell Signaling Technology, 5174S, host: rabbit, Reactivity: Human, Mouse, Rat, where they validated their antibodies by Western blot analysis of extracts from various cell lines: https://www.cellsignal.com/products/primary-antibodies/gapdh-d16h11-xp-rabbit-mab/5174;
Vinculin monoclonal (EPR8185) ab129002, from Abcam, host: rabbit, Reactivity: Human, Mouse, Rat where they validated their antibodies from the house: https://www.abcam.com/vinculin-antibody-epr8185-ab129002.html).
RPE65 monoclonal (JM61-51) (MA-532633, Invitrogen, host: rabbit, Reactivity: Human, Mouse, Rat) was routinely validated by Western blot analysis of RPE65 in mouse eyeball cell lysate: https://www.thermofisher.com/antibody/product/RPE65-Antibody-clone-JM61-51-Recombinant-Monoclonal/MA5-32633;
rhodopsin monoclonal (Rho 4D2) from Abcam, ab98887, host: mouse, Reactivity: Mouse, Rat, Cow, Human, Pig, Xenopus laevis was routinely validated from house: https://www.abcam.com/rhodopsin-antibody-rho-4d2-ab98887.html;
Alexa Fluor 594 conjugated ZO-1 (1:200, Invitrogen, 339194, host: mouse, Reactivity: Chicken, Human, Mouse) was validated by Immunofluorescent staining of ZO-1 in Human Caco-2 cells: https://www.thermofisher.com/antibody/product/ZO-1-Antibody-clone-ZO1-1A12-Monoclonal/339194)
Donkey anti-Rabbit-Alexa Fluor 488 polyclonal (1:200, Invitrogen, A21206, host: Donkey, routinely validated from house, https://www.thermofisher.com/antibody/product/Donkey-anti-Rabbit-IgG-H-L-Highly-Cross-Adsorbed-Secondary-Antibody-Polyclonal/A-21206)
Donkey anti-mouse-Alexa Fluor 488 polyclonal (1:200, Invitrogen, A21202, host: Donkey, was validated: https://www.thermofisher.com/antibody/product/Donkey-anti-Mouse-IgG-H-L-Highly-Cross-Adsorbed-Secondary-Antibody-Polyclonal/A-21202)
Goat anti-Rabbit-Alexa Fluor 568, polyclonal (1:200, Invitrogen, A11011, host: Goat, routinely validated from house, https://www.thermofisher.com/antibody/product/Goat-anti-Rabbit-IgG-H-L-Cross-Adsorbed-Secondary-Antibody-Polyclonal/A-11011)
opsin polyclonal (1:200, AB5405, MilliporeSigma, host: rabbit, Reactivity: monkey, human, mouse, was validated from house, https://www.sigmaaldrich.com/US/en/product/mm/ab5405)
Cre antibody monoclonal (1:200, clone: 2D8, MilliporeSigma, MAB3120, host: mouse, Reactivity: All, was validated from house, https://www.sigmaaldrich.com/US/en/product/mm/mab3120)
Best1 antibody polyclonal (Bioss Antibodies, bs11040Rhost: rabbit, Reactivity: Mouse, Rat, Dog, Cow, Pig, Horse, Rabbit, was validated from house, https://www.biossusa.com/products/bs-11040r)
PDK1 polyclonal (Cell Signaling Technology, 3062, host: rabbit, Reactivity: Human, Mouse, Rat) was validated by Western blot analysis of extracts from SW-13, NIH/3T3, Jurkat and PC12 cells: https://www.cellsignal.com/products/primary-antibodies/pdk1-antibody/3062;
phospho-PDK1 monoclonal (C49H12) (1:500 dilution, 3438, from Cell Signaling Technology, host: rabbit, Reactivity: Human, Mouse, Rat, where they validated their antibodies validated by Western blot analysis of extracts from PC3 cells: https://www.cellsignal.com/products/primary-antibodies/phospho-pdk1-ser241-c49h2-rabbit-mab/3438;
PI3K monoclonal (19H8) from Cell Signaling Technology, 4257, host: rabbit, Reactivity: Human, Mouse, Rat, where they validated their antibodies by Western blot analysis of extracts from Jurkat and NIH/3T3 cells: https://www.cellsignal.com/products/primary-antibodies/pi3-kinase-p85-19h8-rabbit-mab/4257;
phospho-PI3K polyclonal 1:500 dilution, from Cell Signaling Technology 4228, host: rabbit, Reactivity: Human, Mouse, where they validated their antibodies by Western blot analysis of extracts from NIH/3T3-Src cells, C2C12 cells: https://www.cellsignal.com/products/primary-antibodies/phospho-pi3-kinase-p85-tyr458-p55-tyr199-antibody/4228?site-search-type=Products&N=4294956287&Ntt=phospho-pi3+kinase&fromPage=plp;
Monoclonal Anti-Ceramide antibody (15B4), 1:200, from Sigma-Aldrich, C8104, host: mouse, where they validated their antibodies from house: https://www.sigmaaldrich.com/US/en/product/sigma/c8104;
BV650 Rat Anti-Mouse CD11b monoclonal (M1/70) from BD Biosciences, 563402, Reactivity:Mouse (QC Testing), Human (Tested in Development) Isotype:Rat DA, also known as DA/HA IgG2b, κ, was routinely validated by Flow cytometric using mouse bone marrow cells: https://www.bdbiosciences.com/en-us/products/reagents/flow-cytometry-reagents/research-reagents/single-color-antibodies-

ruo/bv650-rat-anti-cd11b.563402;
PE-Cy7 Rat anti-Mouse CD45 from BD Biosciences, 561868, Reactivity: Mouse (QC Testing)Isotype: Rat LOU, also known as Louvain, LOU/C, LOU/M IgG2b, κ, where they validated their antibodies by Flow cytometry using mouse splenocytes: https://www.bdbiosciences.com/en-us/products/reagents/flow-cytometry-reagents/research-reagents/single-color-antibodies-ruo/pe-cy-7-rat-anti-mouse-cd45.552848;
FITC Rat anti-Mouse CCR2 (SC203G11) monoclonal (SA203G11) from BioLegend, 150607, Reactivity: Mouse, where they validated their antibodies by C57BL/6 bone marrow: https://www.biolegend.com/nl-be/products/fitc-anti-mouse-cd192-ccr2-antibody-13354;
APC Rat Anti-Mouse Ly-6G monoclonal, 1A8 (BD Biosciences, 560599) , Reactivity: Mouse (QC Testing) Isotype: Rat LEW, also known as Lewis IgG2a, κ, was validated using mouse bone marrow: https://www.bdbiosciences.com/en-us/products/reagents/flow-cytometry-reagents/research-reagents/single-color-antibodies-ruo/apc-rat-anti-mouse-ly-6g.560599.
BV421 Rat Anti-Mouse Ly-6C monoclonal, (Al-21) from BD Biosciences, 562727, Reactivity: Mouse (QC Testing)Isotype: Rat IgM, κ, where they validated their antibodies using mouse splenocytes: https://www.bdbiosciences.com/en-eu/products/reagents/flow-cytometry-reagents/research-reagents/single-color-antibodies-ruo/bv421-rat-anti-mouse-ly-6c.562727.

Eukaryotic cell lines

Policy information about cell lines

Cell line source(s)	Human fetal RPE cell was a gift from Dr. Ram Kannan, Macular Research Lab
Authentication	The human fetal RPE cells derived from healthy human RPE cells, is primary cells that retains many in vivo phenotypic characteristics. PRE-specific markers such as RPE65 and epithelial markers ZO1 are expressed in the human fetal retinal pigmented epithelial cells.
Mycoplasma contamination	No mycoplasma contamination was found during human fetal RPE culture
Commonly misidentified lines (See ICLAC register)	N/A

Animals and other organisms

Policy information about studies involving animals; ARRIVE guidelines recommended for reporting animal research

Laboratory animals	RPE specific Akt2 cKO and Akt2 KI mice and control Akt2 fl/fl, Best1/Cre and wild type, male and female mice used in this study were 1-9 months of age. Housing was overseen by the DLAR, UPMC Children's Hospital in individually-ventilated polysulfone rodent cages, supplied ventilation via a HEPA-filtered supply, wall-mounted unit. Exhaust is connected to the building's exhaust fans with a flexible hose assembly. The temperature and humidity are controlled and there is a 12 hour light and dark cycle.
Wild animals	No wild animals were used in the study.
Field-collected samples	No field collected samples were used in the study.
Ethics oversight	All animal studies were conducted in accordance with the guide for the care and use of animals (National Academy Press) and were approved by the Institutional Animal Care and Use Committee of the University of Pittsburgh. Protocol number is 20108281.

Note that full information on the approval of the study protocol must also be provided in the manuscript.

Flow Cytometry

Plots

Confirm that:

- [x] The axis labels state the marker and fluorochrome used (e.g. CD4-FITC).
- [x] The axis scales are clearly visible. Include numbers along axes only for bottom left plot of group (a 'group' is an analysis of identical markers).
- [x] All plots are contour plots with outliers or pseudocolor plots.
- [x] A numerical value for number of cells or percentage (with statistics) is provided.

Methodology

Sample preparation	Mouse retina and RPE-choroid were gently dissected and digested with 1.5 mg/ml collagenase A (10103578001, Sigma-Aldrich) and 0.4 mg/ml DNase 1 (11284932001, Sigma-Aldrich) at 37°C for 1 hour. Single-cell suspensions were generated by pipetting the tissue to release cells and passing cells through 70 um filters. Cells were blocked with 1% mouse serum (10410, Thermo Fisher Scientific) and Fc blocker (553142, BD Biosciences), followed by antibody staining including BV650 Rat Anti-Mouse CD11b (563402, BD Biosciences), PE-Cy7 Rat anti-Mouse CD45 (561868, BD Biosciences), APC Rat Anti-Mouse Ly-6G (560599, BD Biosciences), FITC Rat anti-Mouse CCR2 (150607, BioLegend), and BV421 Rat Anti-Mouse Ly-6C (562727, BD Biosciences) in brilliant stain buffer (563794, BD Biosciences) at a concentration of 1 ug/mL for 30 min at room temperature.
Instrument	LSRII
Software	BD LSR analyzer

Cell population abundance	Final cell population abundance is marked on Figure 3 and Supplementary Figure 4
Gating strategy	Debris, dead cells and cell doublets were excluded by side scatter. Wild type non-diabetic mouse retina/RPE samples were used as a negative control for gating cd11b, cd45, Ly6G, Ly6C and CCR2 positive cells.

☒ Tick this box to confirm that a figure exemplifying the gating strategy is provided in the Supplementary Information.
